# Neurophotonic tools for microscopic measurements and manipulation: status report

**DOI:** 10.1117/1.NPh.9.S1.013001

**Published:** 2022-04-27

**Authors:** Ahmed S. Abdelfattah, Sapna Ahuja, Taner Akkin, Srinivasa Rao Allu, Joshua Brake, David A. Boas, Erin M. Buckley, Robert E. Campbell, Anderson I. Chen, Xiaojun Cheng, Tomáš Čižmár, Irene Costantini, Massimo De Vittorio, Anna Devor, Patrick R. Doran, Mirna El Khatib, Valentina Emiliani, Natalie Fomin-Thunemann, Yeshaiahu Fainman, Tomas Fernandez-Alfonso, Christopher G. L. Ferri, Ariel Gilad, Xue Han, Andrew Harris, Elizabeth M. C. Hillman, Ute Hochgeschwender, Matthew G. Holt, Na Ji, Kıvılcım Kılıç, Evelyn M. R. Lake, Lei Li, Tianqi Li, Philipp Mächler, Evan W. Miller, Rickson C. Mesquita, K. M. Naga Srinivas Nadella, U. Valentin Nägerl, Yusuke Nasu, Axel Nimmerjahn, Petra Ondráčková, Francesco S. Pavone, Citlali Perez Campos, Darcy S. Peterka, Filippo Pisano, Ferruccio Pisanello, Francesca Puppo, Bernardo L. Sabatini, Sanaz Sadegh, Sava Sakadzic, Shy Shoham, Sanaya N. Shroff, R. Angus Silver, Ruth R. Sims, Spencer L. Smith, Vivek J. Srinivasan, Martin Thunemann, Lei Tian, Lin Tian, Thomas Troxler, Antoine Valera, Alipasha Vaziri, Sergei A. Vinogradov, Flavia Vitale, Lihong V. Wang, Hana Uhlířová, Chris Xu, Changhuei Yang, Mu-Han Yang, Gary Yellen, Ofer Yizhar, Yongxin Zhao

**Affiliations:** a Brown University, Department of Neuroscience, Providence, Rhode Island, United States; b University of Pennsylvania, Perelman School of Medicine, Department of Biochemistry and Biophysics, Philadelphia, Pennsylvania, United States; c University of Pennsylvania, School of Arts and Sciences, Department of Chemistry, Philadelphia, Pennsylvania, United States; d University of Minnesota, Department of Biomedical Engineering, Minneapolis, Minnesota, United States; e Harvey Mudd College, Department of Engineering, Claremont, California, United States; f Boston University, Department of Biomedical Engineering, Boston, Massachusetts, United States; g Georgia Institute of Technology and Emory University, Wallace H. Coulter Department of Biomedical Engineering, Atlanta, Georgia, United States; h Emory University, Department of Pediatrics, Atlanta, Georgia, United States; i University of Tokyo, Department of Chemistry, Tokyo, Japan; j University of Alberta, Department of Chemistry, Edmonton, Alberta, Canada; k Institute of Scientific Instruments of the Czech Academy of Sciences, Brno, Czech Republic; l University of Florence, European Laboratory for Non-Linear Spectroscopy, Department of Biology, Florence, Italy; m National Institute of Optics, National Research Council, Rome, Italy; n Istituto Italiano di Tecnologia, Center for Biomolecular Nanotechnologies, Arnesano, Italy; o Massachusetts General Hospital, Harvard Medical School, Athinoula A. Martinos Center for Biomedical Imaging, Charlestown, Massachusetts, United States; p Sorbonne University, INSERM, CNRS, Institut de la Vision, Paris, France; q University of California San Diego, Department of Electrical and Computer Engineering, La Jolla, California, United States; r University College London, Department of Neuroscience, Physiology and Pharmacology, London, United Kingdom; s University of California San Diego, Departments of Neurosciences, La Jolla, California, United States; t The Hebrew University of Jerusalem, Institute for Medical Research Israel–Canada, Department of Medical Neurobiology, Faculty of Medicine, Jerusalem, Israel; u Weizmann Institute of Science, Department of Brain Sciences, Rehovot, Israel; v Columbia University, Zuckerman Mind Brain Behavior Institute, New York, United States; w Central Michigan University, Department of Neuroscience, Mount Pleasant, Michigan, United States; x University of Porto, Instituto de Investigação e Inovação em Saúde (i3S), Porto, Portugal; y University of California Berkeley, Department of Physics, Berkeley, California, United States; z Yale School of Medicine, Department of Radiology and Biomedical Imaging, New Haven, Connecticut, United States; aa California Institute of Technology, Andrew and Peggy Cherng Department of Medical Engineering, Department of Electrical Engineering, Pasadena, California, United States; ab University of California Berkeley, Departments of Chemistry and Molecular & Cell Biology and Helen Wills Neuroscience Institute, Berkeley, California, United States; ac University of Campinas, Institute of Physics, Campinas, São Paulo, Brazil; ad Interdisciplinary Institute for Neuroscience University of Bordeaux & CNRS, Bordeaux, France; ae Salk Institute for Biological Studies, Waitt Advanced Biophotonics Center, La Jolla, California, United States; af University of Florence, European Laboratory for Non-Linear Spectroscopy, Department of Physics, Florence, Italy; ag Harvard Medical School, Howard Hughes Medical Institute, Department of Neurobiology, Boston, Massachusetts, United States; ah New York University Grossman School of Medicine, Tech4Health and Neuroscience Institutes, New York, New York, United States; ai Sorbonne University, INSERM, CNRS, Institut de la Vision, Paris, France; aj University of California Santa Barbara, Department of Electrical and Computer Engineering, Santa Barbara, California, United States; ak New York University Langone Health, Departments of Ophthalmology and Radiology, New York, New York, United States; al Boston University, Departments of Electrical Engineering and Biomedical Engineering, Boston, Massachusetts, United States; am University of California Davis, Department of Biochemistry and Molecular Medicine, Davis, California, United States; an Rockefeller University, Laboratory of Neurotechnology and Biophysics, New York, New York, United States; ao The Rockefeller University, The Kavli Neural Systems Institute, New York, New York, United States; ap Center for Neuroengineering and Therapeutics, Departments of Neurology, Bioengineering, Physical Medicine and Rehabilitation, Philadelphia, Pennsylvania, United States; aq Cornell University, School of Applied and Engineering Physics, Ithaca, New York, United States; ar California Institute of Technology, Departments of Electrical Engineering, Bioengineering and Medical Engineering, Pasadena, California, United States; as Harvard Medical School, Department of Neurobiology, Boston, Massachusetts, United States; at Carnegie Mellon University, Department of Biological Sciences, Pittsburgh, Pennsylvania, United States

**Keywords:** optical imaging, molecular sensors, optogenetics, fluorescence, label free, blood flow, multimodal

## Abstract

*Neurophotonics* was launched in 2014 coinciding with the launch of the BRAIN Initiative focused on development of technologies for advancement of neuroscience. For the last seven years, *Neurophotonics*’ agenda has been well aligned with this focus on neurotechnologies featuring new optical methods and tools applicable to brain studies. While the BRAIN Initiative 2.0 is pivoting towards applications of these novel tools in the quest to understand the brain, this status report reviews an extensive and diverse toolkit of novel methods to explore brain function that have emerged from the BRAIN Initiative and related large-scale efforts for measurement and manipulation of brain structure and function. Here, we focus on neurophotonic tools mostly applicable to animal studies. A companion report, scheduled to appear later this year, will cover diffuse optical imaging methods applicable to noninvasive human studies. For each domain, we outline the current state-of-the-art of the respective technologies, identify the areas where innovation is needed, and provide an outlook for the future directions.

## 1 Introduction

The Neuron Doctrine formulated by Ramón y Cajal at the turn of the 19th century is an embodiment of neuroscience discovery empowered by optics. Cajal used Camillo Golgi’s “black reaction” to sparsely impregnate neurons with light-absorbing silver nitrate in histological brain sections. Inspecting these sections with a light microscope, Cajal concluded that information was flowing in one specific direction—from dendrites to axons—within a network of discrete nerve cells connected by synapses. This conceptual advance, enabled through utilization of a light-absorbing probe in combination with widefield microscopy, laid the foundation for modern neuroscience. Fast-forward to the present day, and optical tools for measurement and manipulation of neuronal activity are so ubiquitous and versatile that “neurophotonics” has become a common word in the scientific vocabulary: we talk about neurophotonic technologies and research centers, we have the *Neurophotonics* journal, and recognize neurophotonics as its own field of research.

While optical methods have occupied center stage in neuroscience from the times of Cajal and Golgi, the last decade has seen a rapid advance of neurophotonic technologies, in large part thanks to the BRAIN Initiative^1^^,^^2^ as well as other large-scale neuroscience projects in the US and around the world.^3^ At the dawn of the BRAIN Initiative, the research community was deeply engaged in discussion about what kinds of tools were needed to accelerate neuroscience discovery. Questions were asked about the technological goals to maximize the progress in understanding the brain: Should we measure each spike in every neuron? Should we prioritize one model species over another? Should we pick one specific neuronal circuit and summon all forces to understand it as completely as we can? After intense debates in the media, townhall meetings and deliberations at the National Institutes of Health and beyond, the final answer was: We need it all! We need to understand the brain across scales, from its molecular composition to large-scale architecture, from single-cell gene expression to whole-brain activity patterns, and for each aspect of the brain’s organization, we need to use those model species that make the most sense. Thus, we also need a variety of tools with which to tackle these different biological parameters and phenomena. Halfway through the BRAIN Initiative, we can safely say that this inclusive scope is paying off. We now have a large array of diverse experimental and computational tools to study the brain across species, scales, levels of description, in animals and humans. Notably, the lion’s share of these technologies falls under the general umbrella of neurophotonics.

Here, we review the current state-of-the-art tools that are, in general, applicable to animal models and usually considered under the umbrella of microscopy. We start with an overview of high-resolution structural imaging tools that have emerged and/or matured within the last decade. Then, we highlight several newly developed optical sensors and modulators of brain activity followed by discussion of a suite of one-photon (1P) and multiphoton fluorescence microscopy methods aimed at large-scale imaging of brain activity with high temporal resolution. We include a number of technologies for imaging cerebral blood flow and metabolism and feature several imaging approaches that integrate optical tools with other measurement modalities providing complementary information and/or helping to overcome fundamental limitations of purely optical methods. Finally, we highlight the importance of computational tools that complement optical technologies extending their capabilities.

Given the vast and rapidly expanding landscape of neurophotonic tools, the current scope is not exhaustive but rather biased on the collective scientific interest of this group of authors. Most often, these tools entail some level of invasiveness and in general are not applicable to live brain imaging in humans. A companion paper is focused on noninvasive (diffuse) optical imaging technologies targeted for humans.

## 2 Imaging of Microscopic Brain Structure

A singular advance that underlies the modern high-resolution, high-throughput imaging of labeled brain structures in animal species is the development of a diverse palette of bright, photostable fluorescent proteins.^4^ These proteins are genetically encoded, which allows targeting of fluorescent labeling to specific cells, subcellular compartments, and biochemical events (e.g., gene transcription). In parallel, advancement of new biochemical methods of tissue index matching, building upon the original CLARITY protocol,^5^ have enabled volumetric acquisition of samples as thick as the whole mouse brain. On the imaging side, light-sheet microscopy^6^ has emerged as a method of choice for high-throughput acquisition of cleared brain samples.^7^ Beyond the diffraction-limited resolution of light microscopy, a suite of super-resolution technologies has leveraged the principle of manipulating the ‘on’ and ‘off’ state of the fluorescent labels.^8^^,^^9^ Expansion microscopy^10^ provides an alternative solution for overcoming the diffraction limit by physically expanding the sample. Expansion microscopy can be used to view small structures, such as synaptic vesicles, with no need for complex super-resolution instrumentation, but, in contrast to the “classical” super-resolution methods, is not compatible with live imaging.

Complementary to fluorescent labeling, label-free imaging relies on intrinsic properties of brain tissue.^11^^–^^16^ High-resolution label-free structural imaging is of particular relevance for human brain tissue, where expression of fluorescent proteins does not apply for neuroethics reasons. Label-free imaging techniques leverage optical phenomena of interaction of light with live and fixed brain tissue including autofluorescence, reflectance, birefringence (polarization and direction dependence of refractive index) that yields retardance and changes in light polarization and scattering.

### 2.1 Fluorescent Proteins

Naturally occurring fluorescent proteins (FPs) from various marine organisms, including the archetypical *Aequorea victoria* green FP (avGFP),^17^ provide the foundation for all genetically encoded molecular probes for fluorescence imaging of brain structure and function used today. The gene for an FP is introduced into the tissues of a model organism and then transcribed and translated by the normal cellular machinery. Expression in specific neuronal cell types or brain regions can be achieved with appropriate combinations of gene enhancers and promoters, adeno-associated virus (AAV) serotypes and injection site, and recombinase-dependent expression strategies. Having inspired countless genetic manipulation strategies, numerous advances in microscopy, and the development of powerful functional imaging probes, FPs are the substrate upon which a vast ecosystem of neurophotonic technologies now flourish.

**Fig. 1:**
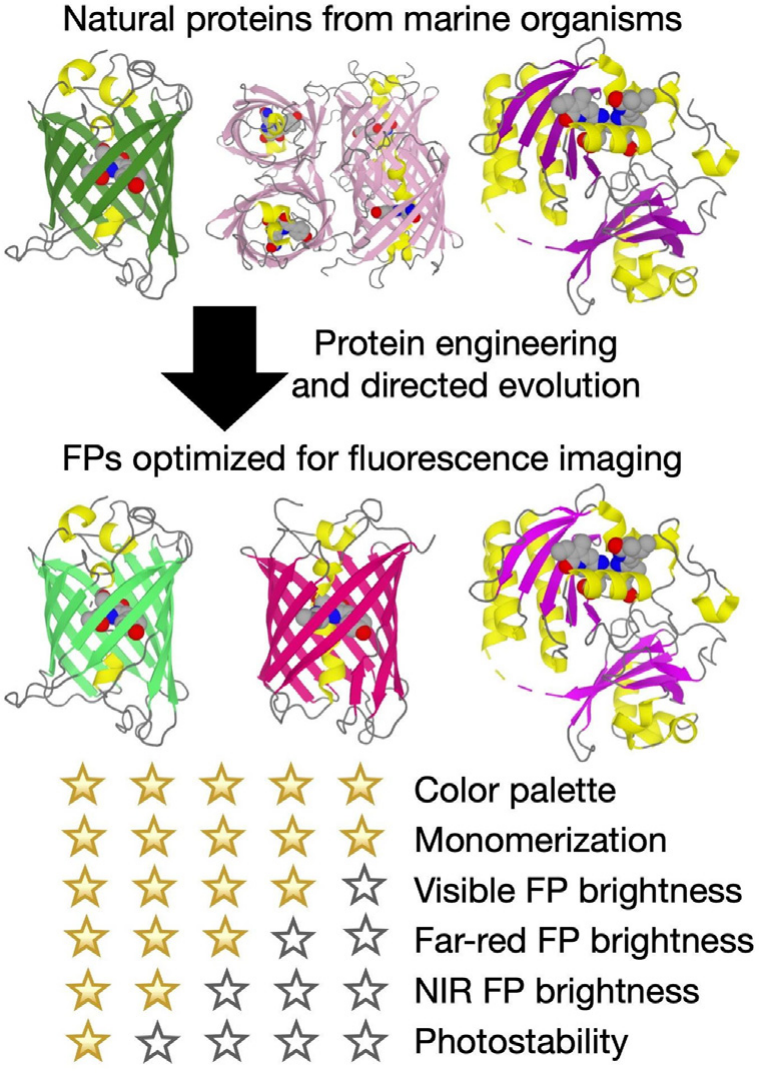
Fluorescent protein engineering.

Essentially all FPs in use today have been engineered using their wild-type progenitors as a starting point (Fig. 1). Nature and natural evolution have played the biggest role in providing researchers with a diversity of FP-based tools, and undoubtedly there are many secrets yet to be revealed.^18^ However, for adapting natural FPs to the unnatural demands of neurophotonic applications, laboratory engineering and directed evolution play essential roles. Such approaches have been overwhelmingly successful at providing a color palette of bright FPs for multi-color, multi-parameter imaging,^19^ and monomeric FPs for use as minimally-perturbing fusion protein labels.^20^ However, these efforts have been only partially successful at providing high-performance far-red FPs,^21^ brightly fluorescent near-infrared FPs,^22^ or substantial improvements in photostability.

Looking to the future, new protein engineering methods, or more intensive application of established methods, will be critical for overcoming these and other limitations. It is impossible to predict exactly which approaches are most likely to provide the next generation of FPs but, based on precedent, progress is certain to be achieved in a piecemeal fashion. For example, complementary approaches of particular promise include innovative library screening schemes^23^ and rational computational design.^24^ For some applications, alternative approaches such as semi-synthetic fluorogenic proteins,^25^^,^^26^ and bright luciferase plus luciferin pairs,^27^^,^^28^ may supplant FPs. In one form or another, FPs, or their functional equivalents, will remain at the forefront of neurophotonic research for the foreseeable future.

### 2.2 Tissue Clearing for High-Throughput, High-Resolution Imaging of Macrobiomolecules

Classic histological protocols for labeling of fixed tissue sections often include a step of dehydration followed by “clearance.” In the process of dehydration, the water is removed and replaced by the alcohol (ethanol). In the process of clearance, a clearing agent (often methyl salicylate) is used to displace the ethanol while also removing fat molecules rendering the tissue optically transparent. In neuroscience, these protocols have been widely used in combination with bright field and epi-fluorescence microscopy for morphological analyses, i.e., for reconstruction of dendritic and axonal arbors across different neuronal cell types.^29^ While useful, these methods were limited to relatively thin brain sections to allow the chemicals to penetrate throughout. In addition, uneven shrinkage of serial sections prevented stitching sections into volumes. More recently, transition to organic solvents has been proposed as a way to increase sample thickness enabling volumetric reconstruction.^30^ However, the problem of uneven shrinkage remained. In addition, these solvents produced bleaching of endogenous fluorescence, and their toxic and corrosive nature required specialized equipment and handling. As an alternative, aqueous solutions with high refractive index were employed preventing bleaching but not achieving sufficient transparency for thick samples (reviewed in Ref. 31).

**Fig. 2:**
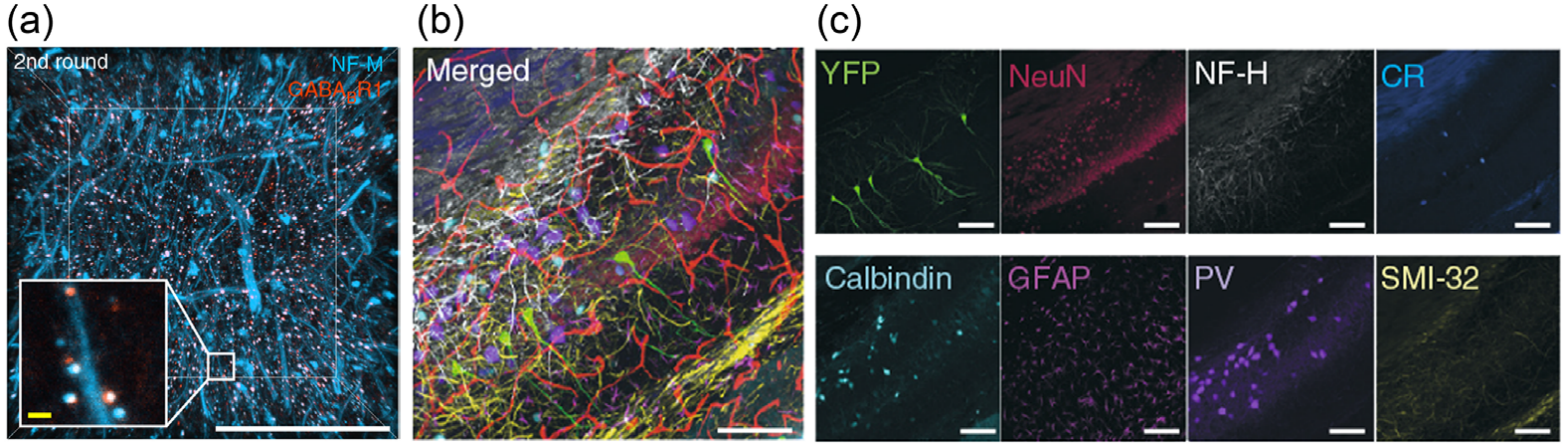
Visualization of biomolecules in cleared tissue. (a) Imaging of neurofilament medium unit (NF-M) and GABAB receptor subunit-1 antibodies in cleared, expanded tissue. Scale bars, 50  μm. (b)–(c) Overlay of multi-round immunostained images. Scale bar, 100  μm. Panel (a) is adapted from Ref. 32; panels (b) and (c) are adapted from Ref. 33.

In 2013, development of CLARITY,^5^ a method of making large volumes of tissue optically transparent and macromolecule-permeable using a hydrogel built from within, revolutionized brain histology. This method has broken ground for the development of a growing family of tissue clearing tools with varying hydrogel composition, including those that can repeatedly expand and shrink brain tissue^32^ while preserving target macrobiomolecules.^33^ In combination with light-sheet microscopy (Sec. 4.3), these methods allow high-throughput analysis of large-scale FP expression and also support multiplexed immunostaining with many rounds of fluorescent antibody labeling and stripping^34^ (Fig. 2).

### 2.3 Super-Resolution Microscopy

**Fig. 3:**
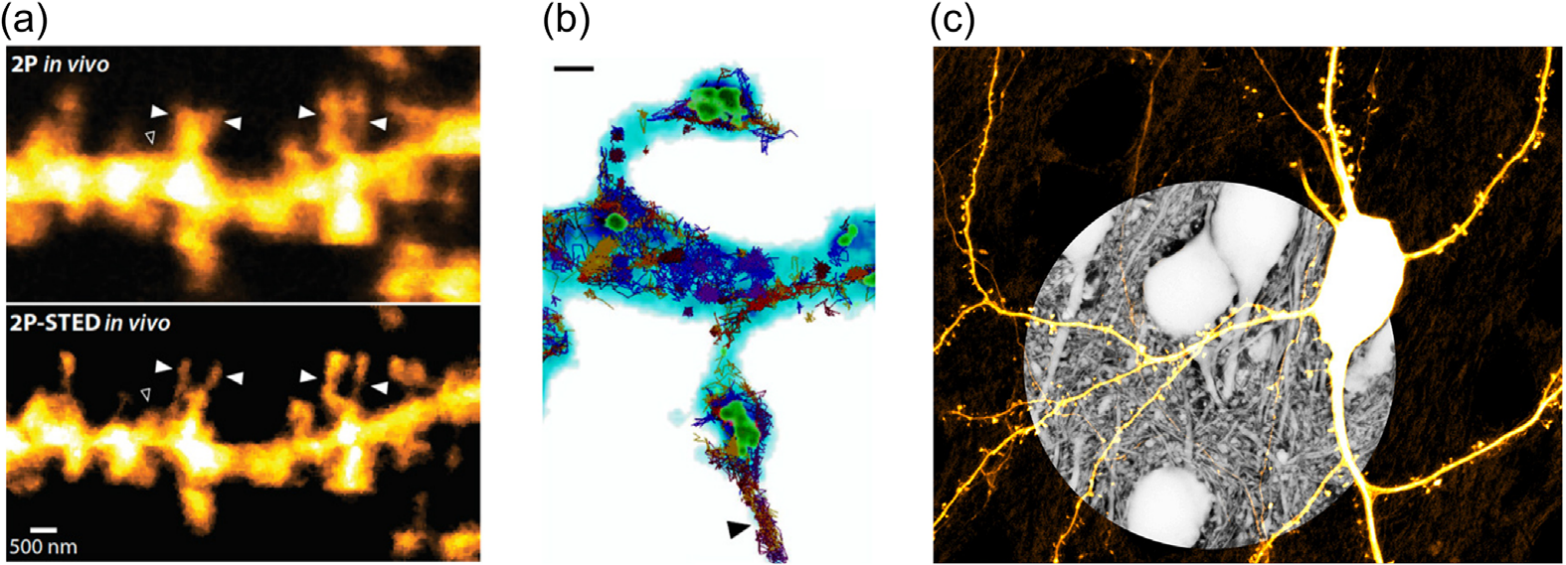
Super-resolution imaging of fine neuronal structures. (a) Comparison of 2P *versus* 2P-STED image of dendritic spines imaged in the mouse hippocampus *in vivo* (adapted from Ref. 36). (b) Correlative STED and SMLM image of dendritic spine morphology and dynamic spatial arrangement of synaptic proteins (blue, neuronal morphology; green, PSD-95 scaffold protein; colored tracks, GluA1 receptor subunit), scale bar 500 nm (adapted from Ref. 37). (c) Super-resolution shadow imaging (SUSHI) of brain tissue, revealing anatomical context of a YFP-labeled neuron (adapted from Ref. 38).

Super-resolution microscopy refers to a new class of fluorescence imaging techniques that have shattered the diffraction barrier of light microscopy, offering spatial resolutions down to the single-digit nanometer range close to the ultimate limit, i.e. the size of the fluorescent molecules. Since their invention some 20 years ago (recognized by the Nobel Prize in 2014), a diversifying family of super-resolution methods has been developed and deployed in neuroscience research, including STED, RESOLFT, PALM, STORM, u-PAINT, and SIM, encompassing laser-scanning and widefield imaging modalities^35^ (Fig. 3). While these techniques are quite distinct in design and use (concerning hardware, labeling, image analysis), they are all based on the principle of manipulating the ‘on’ and ‘off’ state of the fluorescent labels, whose spatial distribution can be read out in a diffraction-unlimited way.

In STED microscopy,^39^ dye molecules are switched off by stimulated emission in a donut-shaped region around the excitation spot that scans the sample, shrinking its size. In PALM, STORM and u-PAINT, collectively referred to as SMLM (single-molecule localization microscopy), the fluorophores are imaged onto a camera, but not all at the same time. They are turned on so sparsely that each of them can be precisely localized. By acquiring thousands of images of different sets of activated fluorophores, a super-resolved image is constructed.^40^

These ground-breaking methods have enabled unprecedented optical access to subcellular organelles and protein arrangements in living brain cells and tissue preparations, unlocking a wealth of new and exciting biological information. For instance, they have revealed the ring-like organization of the cytoskeleton in axons,^41^ the columnar arrangement of pre- and postsynaptic signaling proteins,^42^ the structural plasticity of dendritic spines in the hippocampus,^36^ as well as basic physical properties of the extracellular space of brain tissue.^43^

In the future, this technology will still develop rapidly in many key respects, allowing for more sophisticated experiments on more complex and biologically relevant samples, while also becoming less cumbersome and expensive. An important task is to make *in vivo* super-resolution imaging more of a reality, which faces special challenges in terms of depth penetration and brain motion. We can expect that recent technological advances, including those in adaptive optics and computational tools, will substantially potentiate the power of super-resolution microscopy and the biological insights that it will unearth.

### 2.4 Expansion Microscopy

The fundamental optical diffraction limit precludes the use of conventional light microscopy for imaging of nanoscopic structures such as the post-synaptic density. Expansion Microscopy (ExM)^44^ overcomes this limitation by physically magnifying tissues while reserving or highlighting biomolecules such as proteins, nucleic acids, lipids, etc.^45^^,^^46^

The general workflow of ExM involves the following steps: (1) infusion and synthesis of a water-swellable hydrogel throughout the specimen; (2) chemically linking biomolecules of interest to the hydrogel; (3) homogenization of the specimen enzymatically or via heat denaturation, and (4) physical expansion of the specimen in pure water.^47^ The workflow is designed to facilitate isotropic expansion.^10^^,^^44^^,^^48^^–^^51^ Most ExM methods expand the specimen by up to ∼4.5× in the linear dimension. Using a conventional light microscope, such expansion provides ∼60  nm image resolution for an objective lens with 270 nm diffraction limit (270/∼4.5=∼60  nm). If protocols for 10× expansion^52^ or more^53^ are used, the effective imaging resolution can be further improved to ∼25−30  nm.

**Fig. 4:**
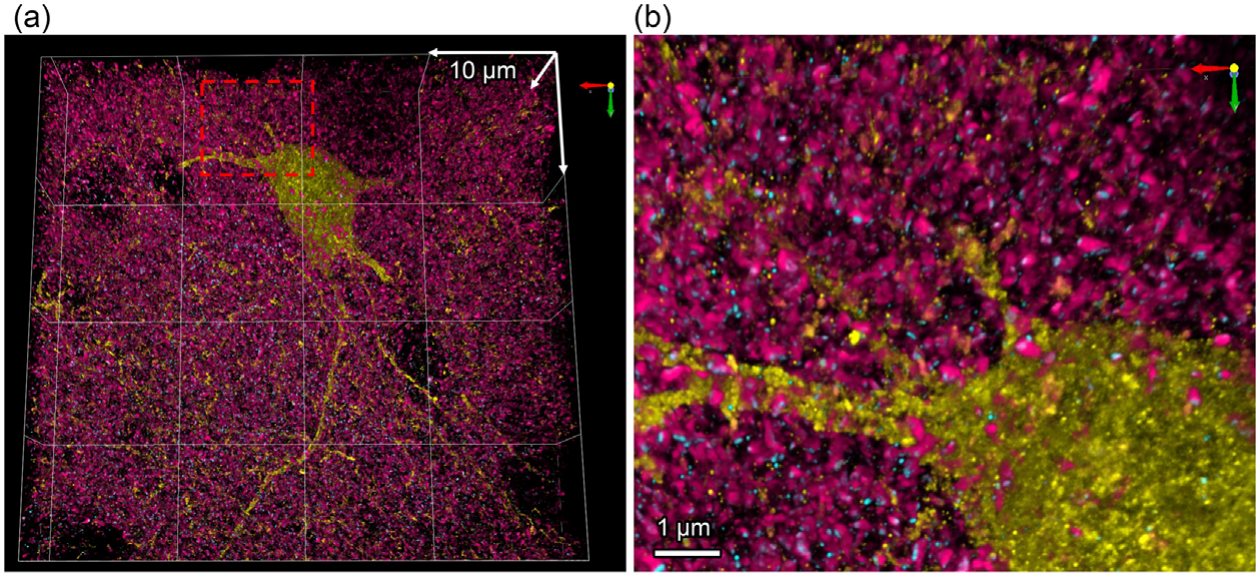
Expansion microscopy imaging of synaptic proteins. (a) Volumetric image of an inhibitory neuron genetically labelled with EYFP in a 4× expanded mouse brain section stained with antibodies against EYFP (yellow), synaptophysin (magenta) and PSD95 (cyan). (b) Zoom-in view from (a), as indicated by the dashed red box.

This technology has been used to (1) study synapses in a number of model organisms including mouse brains^44^^,^^51^ (Fig. 4), larval and adult *Drosophila* brains,^54^^,^^55^ *C. elegans*,^56^ planarian glia,^57^ and larval zebrafish, revealing heterogeneity in synapse structure^58^ and age-associated changes in active zones;^54^ (2) trace neurons in large volume;^32^^,^^59^ and (3) interrogate neurological diseases, such as Parkinson’s disease,^60^ schizophrenia,^61^ and dementia,^62^ discovering disease-related subtle structural changes^61^^,^^63^ and protein mislocalization.^62^

In the future, we expect ExM will further advance (1) labeling strategies to overcome the bottleneck in the signal strength that is diluted by expansion itself; (2) new gel chemistry that lead to larger expansion and better resolution; (3) multiplexing strategies to address the limitation of the 4–5 colors due to spectral overlap of fluorophores; (4) development of optical objectives and microscopes and the associated imaging software specifically for imaging expanded brains or other tissues. In the next decade, we envision researchers will be able to routinely acquire large volume and highly multiplexed nanoscale images of expanded neuronal tissue to quantify changes in synaptic components and other nanoscale structure in the brain, potentially providing new insights into the molecular mechanisms of neuronal function and disease.

### 2.5 Label-Free Structural Imaging Based on Interaction of Light with Tissue

**Fig. 5:**
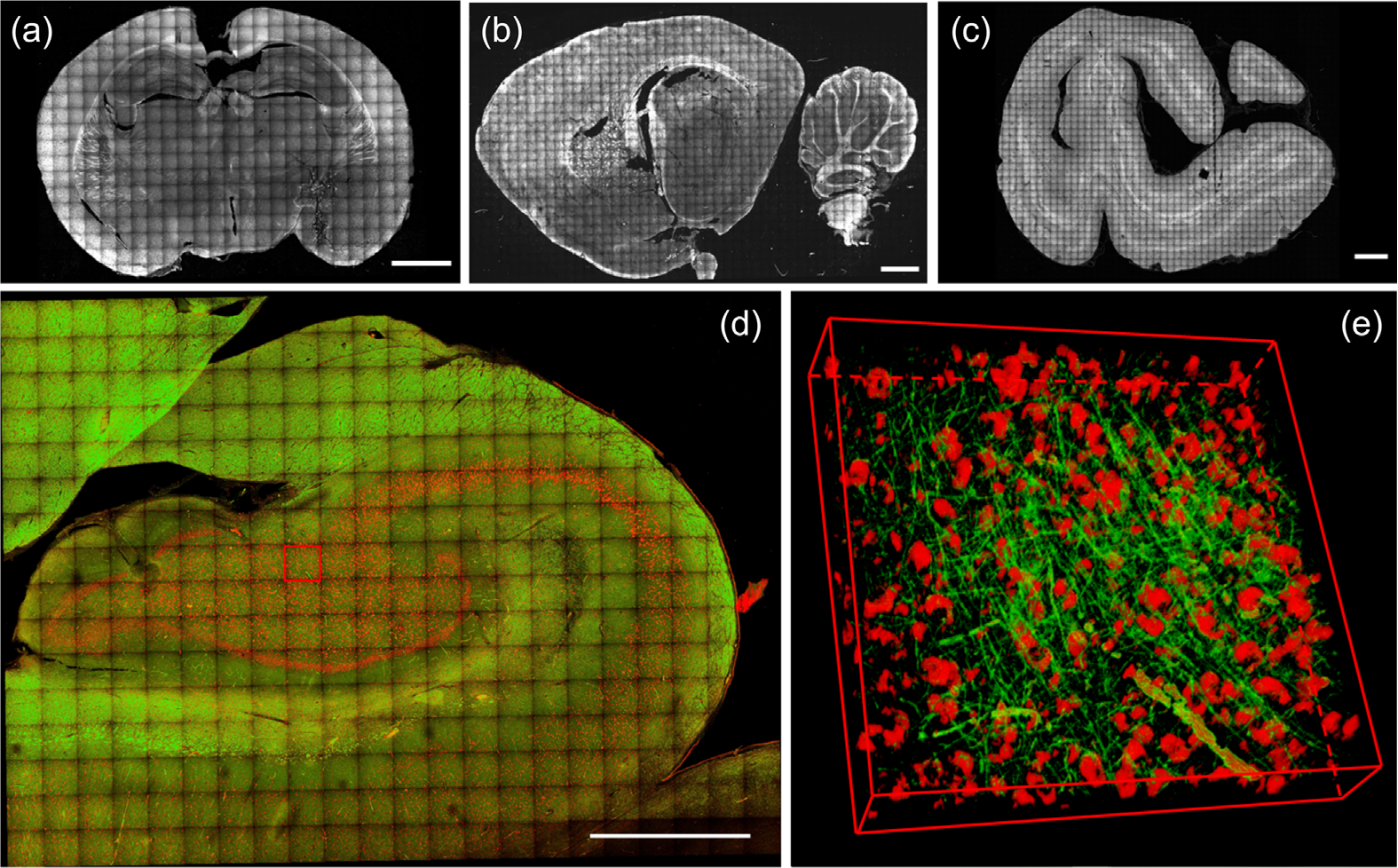
Reconstruction of brain sections treated with MAGIC: (a) mouse, (b) rat, (c) vervet monkey, (d) and human. Scale bar = 1 mm. (e) 3D rendering ($450\times 450\times 60\;\mu m^{3}$) of the stack indicated by the red box in d; green and red channels show the myelinated fibers enhanced by MAGIC and the autofluorescence of the cell bodies produced by lipofuscin pigments. Adapted from Costantini et al.^11^

While fluorescent proteins offer selective genetic targeting and multiplexing, these approaches are limited to animal studies where genetic manipulations and editing can be performed. Label-free imaging relies on intrinsic properties of brain tissue and applies to human cerebral tissue, freshly excised or fixed. Indeed, the biological structure autofluorescence produced by red blood cells retained inside the vessels, and the lipofuscin pigments present inside the neuronal bodies, can be detected by fluorescence microscopy techniques.^64^ In addition, it is possible to modify the sample with specific preparations in order to increase the autofluorescence signals of particular structures. Among them, a recent protocol called MAGIC (Myelin Autofluorescence imaging by Glycerol Induced Contrast enhancement)^11^ enables to perform label-free fluorescence imaging of myelinated fibers in various mammalian brains, including humans, with a glycerol-based tissue treatment (Fig. 5) allowing three-dimensional (3D) connectomics analysis of the brain.

Furthermore, optical methods based on different physical phenomena are extensively used to investigate both *in vivo* and *ex vivo* tissues. Raman scattering and its derivation as Coherent anti-Stokes Raman scattering (CARS)^65^ permits to determine the molecular composition of every tissue. Second-harmonic generation (SHG) allows to detect well organized structures like collagen, myosin, or polysaccharides^66^^,^^67^ while third-harmonic generation (THG) distinguishes the interface between different compartments like skin, neuron’s myelin, and adipocyte.^68^ Another label free technique that enables the high-resolution analysis of myelinated fibers is 3D-PLI (Polarized Light Imaging),^69^ based on the detection of the birefringence of the myelin sheaths surrounding axons. In particular, this technique allows the reconstruction of the 3D organization of the whole brain fiber’s architecture in thin slices.^70^ Finally, reflectance microscopy^12^^,^^14^ can be used to measure myelin contents and capillary blood flow in live preparations.

All these methodologies are usually linked together to obtain a multimodal mapping of the different tissue components.^65^^,^^71^ We believe that in future some of the label-free techniques will be combined with specific tissue preparation and labeling, such as clearing methods,^31^ as already done with CARS on mouse brain,^72^ coupling the morpho-chemical label free analysis with labeled 3D volumetric imaging on large samples.^73^^,^^74^ The combination of label-free methods with specific labeling will allow a more comprehensive analysis of tissue composition offering the possibility of molecular phenotyping at the cellular and subcellular level.

### 2.6 Optical Coherence Tomography

Optical coherence tomography (OCT)^75^ is another label-free imaging modality that uses intrinsic contrast to generate cross-sectional or volumetric images of tissues at micrometer resolution. OCT differs from the above-mentioned label-free tools in that it employs low-coherence interferometry to provide depth-resolved images, for which light backscattered and reflected from a sample is mixed with a reference light. Not only the intensity, but also the phase and polarization of the detected light carry physical information. Various OCT-based techniques have been developed for different fields, such as ophthalmology, most notably, but also in cardiology, and dermatology, to visualize tissue structure and function.

**Fig. 6:**
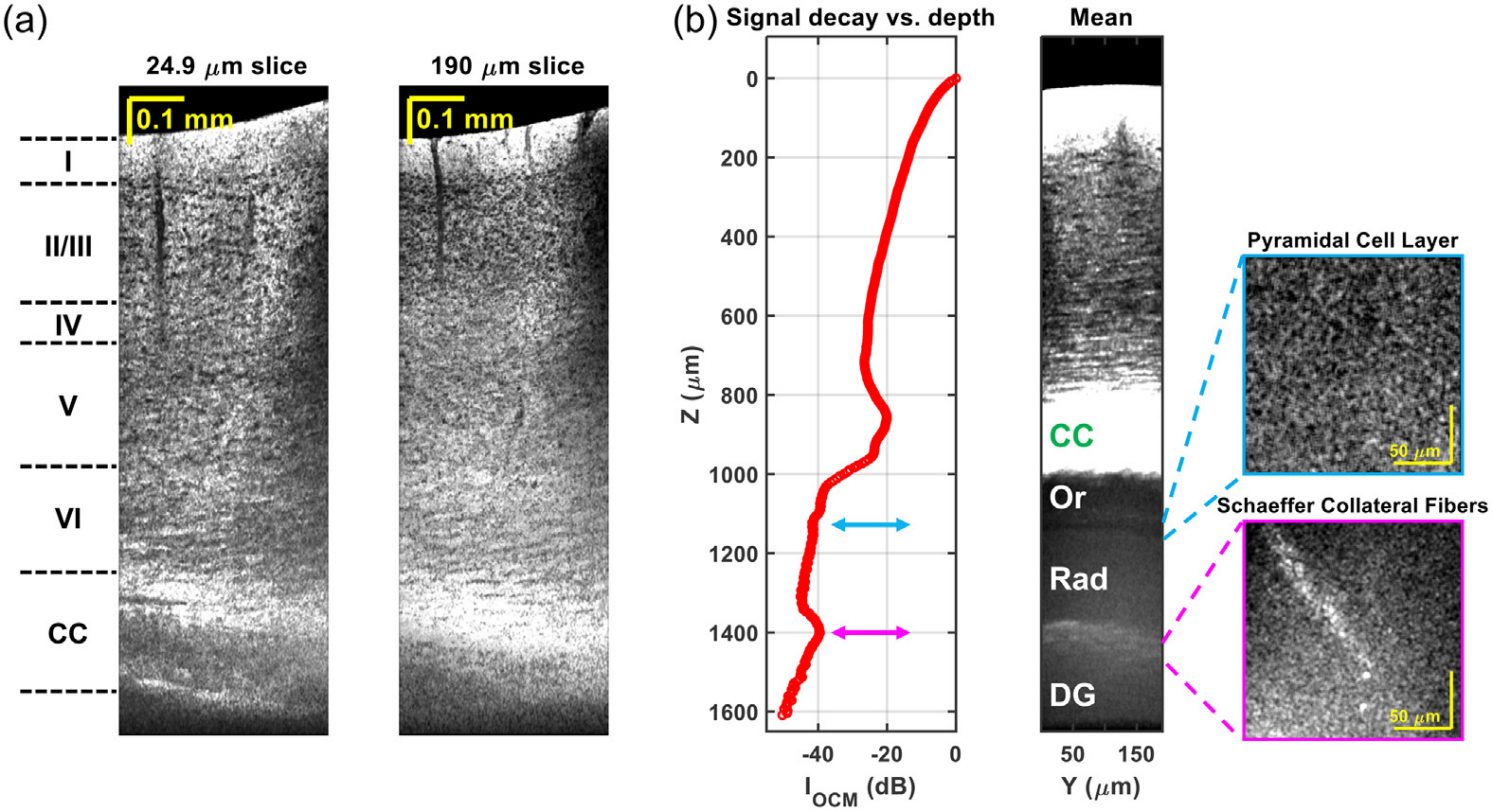
*In vivo* 1700-nm optical coherence microscopy (OCM) of the mouse brain through a thinned skull preparation. (a) Minimum intensity projection sagittal images, or “slices,” with different projection thicknesses in the coronal direction, show cortical cytoarchitecture and the corpus callosum (CC), without physical tissue slicing. (b) OCM signal decay (left panel) and averaged coronal image (middle panel) show sub-cortical layers. CC, corpus callosum; Or, stratum oriens; Rad, stratum radiatum; DG, dentate gyrus. Adapted from Zhu et al.^83^

OCT applications in the brain have been emerging in recent years.^76^ Like the retina, many regions of the brain possess an internal organization which gives rise to natural differences in optical properties. The simplest example of this principle is the fact that white matter has a higher scattering coefficient than gray matter. On a finer scale, cellular composition, myelination, and potentially, axonal/dendritic content can also modulate scattering coefficients of individual layers in the cortical column.^77^ Within gray matter itself, high resolution Optical Coherence Microscopy (OCM) imaging has revealed individual myelinated axons^78^ and neuronal cell bodies,^77^ and potentially cell nuclei.^79^ Morphological changes can accompany cell death^80^^,^^81^ and therefore inform about neuronal viability, while Aβ plaques are distinguished as being highly backscattering.^82^ Finally, OCT is unique in enabling optical microscopic imaging more than 1 mm deep *in vivo* in scattering tissue, through minimally invasive preparations (Fig. 6), and can capture deep pathology that is missed by more superficial imaging techniques.^83^ Functional and hemodynamic extensions of OCT are discussed in Sec. 6.1.

**Fig. 7:**
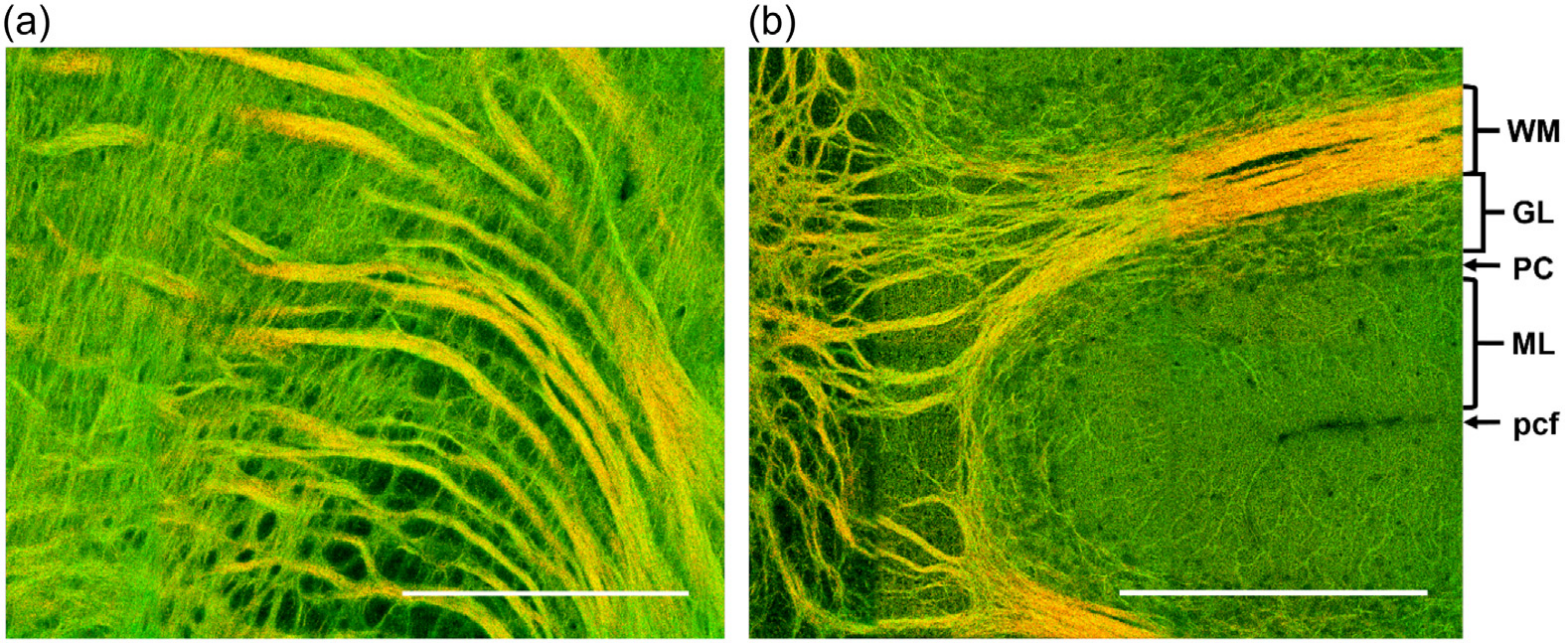
*Ex vivo* serial optical coherence scanning (SOCS) of the mouse brain. (a)–(b) Composite images with reflectivity (green) and retardance (red) contrasts for selected regions in mouse brainstem (a) and cerebellum (b). WM, white matter; GL, granular layer; ML, molecular layer; pcf, preculminate fissure. PC indicates a layer of Purkinje cell bodies whose dendrites reside in ML. Scale bar: 320  μm (length of a square tile).

Interestingly, much of the intrinsic contrast of *in vivo* neuronal tissue is also apparent *ex vivo* in fresh^84^ and to some extent, even fixed^85^^,^^86^ tissues. This has enabled large-scale reconstructions of animal and human brains by serial optical coherence scanning (SOCS) that combines an OCT and a tissue slicer.^15^ The SOCS is a new tool for fundamental research into the anatomical and connectional architecture of the brain without using exogenous contrast agents or expression of foreign genes. It relies on conventional or polarization-sensitive OCT of a volume within the field of view, translating the sample laterally to image neighboring volumes, removing a layer of tissue using the slicer to expose deeper regions for imaging, and repeating these steps until the entire block is imaged. Consequently, the SOCS provides large datasets for brain imaging and mapping, and the data size is proportional to the sample volume and inversely proportional to the volumetric spatial resolution. The latter can be enhanced by utilizing OCM. The technology has been used to distinguish the gray matter and white matter,^87^ compared with Nissl staining^86^ and diffusion MRI,^88^^,^^89^ and applied on the rat brain,^15^ mouse brain,^13^^,^^90^^–^^92^ and human medulla oblongata.^88^ Figure 7 shows the power and promise of the *ex vivo* SOCS method, as it shows axon fibers of mouse brainstem and cerebellum and delineates the cerebellar layers without histological processing.

While *ex vivo* OCT provides intrinsic contrast, it is most powerful when complemented by other, more specific histological or labelled imaging techniques. In the future, we can expect the use of SOCS to complement other serial imaging modalities in creating unique and undistorted datasets from rodent, nonhuman primate, and human brains. Development of image processing and analysis tools will allow creation of new brain atlases and better understanding of the connectome. These could facilitate studying atrophies in brain diseases^93^ and finding new targets for neuromodulation treatments. The necessity of serial imaging and analysis of big data would require implementation of automated protocols to realize these highly significant goals.

## 3 Molecular Sensors and Actuators for Imaging and Manipulation of Brain Activity

Recent advances in molecular engineering have led to several families of high-quality optical sensors. We use the terms “sensor,” “indicator,” and “probe,” interchangeably, to broadly describe molecules that provide a measurable signal in response to changes in brain activity. Following the traditional definition of the term in the analytical sciences, we use “biosensor” to refer to only those sensors in which a protein component is detecting the biological change of interest. Combined with advanced microscopy, calcium ion (${\mathrm{Ca}}^{2+}$) imaging using GCaMPs^94^ is now routinely employed to enable large-scale, single- or multiphoton imaging for readout of neuronal circuit activity in awake, behaving animals.^95^^,^^96^ In parallel, voltage sensors have been significantly improved in recent years in terms of intrinsic properties such as membrane localization, brightness, sensitivity and kinetics. These voltage sensors can be divided into three general categories: synthetic sensors,^97^ hybrid biosensors that use a combination of synthetic dyes and genetically encoded proteins,^98^ and fully genetically encoded biosensors.^99^^–^^101^ Synthetic voltage sensors have a long history in neuroscience including the latest red-shifted photoinduced electron transfer (PeT) highlighted below.^102^ Hybrid voltage biosensors combine molecular specificity of genetically encoded proteins with unsurpassed photophysical properties of synthetic fluorophores. Genetically encoded voltage biosensors are based on either opsins or voltage sensing domains; the most recent of them approach the performance of their synthetic counterparts.^103^ The choice of voltage sensor is driven by applications, and no single solution fits all.^104^

Beyond ${\mathrm{Ca}}^{2+}$ and voltage, more recent protein engineering efforts have now extended the concept of single-FP based biosensors to the design of genetically encoded biosensors for neurotransmitters and neuromodulators, including glutamate, GABA, dopamine, norepinephrine, serotonin and acetylcholine, based on either G-protein coupled receptors (GPCR) or bacterial periplasmic binding proteins (PBP).^105^ These biosensors enable measurements of neurotransmitter release with high spatiotemporal resolution and molecular specificity across the full course of behavioral paradigms.^106^^–^^117^

With growing appreciation for the concepts of the neurovascular unit^118^ and tripartite synapse,^119^ there has been a concerted effort for development of fluorescent biosensors for cellular metabolism,^120^^,^^121^ and targeting these and other biosensors to not only neurons but also non-neuronal cells including astrocytes (as well as immune and vascular mural cells).^122^

The sensors mentioned above, whether genetically encoded, hybrid or synthetic, employ fluorescence to report activity. Another notable form of luminescence is phosphorescence that has a longer lifetime and thus occurs on a slower time scale. Phosphorescence has been traditionally employed in sensing $O_{2}$, a physiological parameter of key importance in the brain and elsewhere.^123^ Development of new 2P-excitable, red-shifted probes has recently enabled deep intravascular and tissue imaging of the partial pressure of $O_{2}$ (${\mathrm{pO}}_{2}$).^124^^,^^125^

Fluorescence and phosphorescence require that photons are delivered to the chromophore/phosphor molecule to induce emission. In bioluminescence, in contrast, emission of light occurs in the process of a chemical reaction where an enzyme oxidizes a substrate.^126^ Bioluminescence has been recently harnessed to generate biosensors though functional reconstitution in the presence of target molecules.^127^ It also has been explored for actuation by using emitted light to stimulate light-sensing proteins.^128^ Although the concept of bioluminescent actuators is very new, this work already has yielded promising results.^129^ Bioluminescent actuation joins optogenetics, a widely used tool for stimulation and inhibition of neuronal activity that has revolutionized neuroscience since its invention in 2005.^130^^,^^131^

### 3.1 Calcium Sensors

${\mathrm{Ca}}^{2+}$ measurements are central in neuroscience due to the importance of ${\mathrm{Ca}}^{2+}$ in cellular excitability and intracellular signaling pathways.^132^ A correlation of ${\mathrm{Ca}}^{2+}$ signals with electrophysiological measurements of membrane potentials across neuronal compartments (soma, proximal dendrites, distal dendrites) has been demonstrated using simultaneous intracellular electrophysiological recordings.^133^^,^^134^ This is due to abundant expression of numerous types of voltage-gated ${\mathrm{Ca}}^{2+}$ channels.^135^^–^^137^

Imaging of neuronal activity using ${\mathrm{Ca}}^{2+}$ sensors was originally enabled by Roger Y. Tsien and colleagues who developed the fura-2 synthetic ${\mathrm{Ca}}^{2+}$ indicator dye and the means to deliver this dye to tissues in the form of an acetoxymethyl (AM) ester.^138^^,^^139^ Following these initial breakthroughs, examples of ${\mathrm{Ca}}^{2+}$ imaging to record neuronal activity soon started to appear.^140^^,^^141^ Ever since, the development of improved ${\mathrm{Ca}}^{2+}$ sensors has been a steadily evolving area in neuroscience due to arrival of new indicators with improved signal to noise ratio (SNR) properties, low toxicity, and optimized delivery strategies. Of particular note was the development of an exceptionally good ${\mathrm{Ca}}^{2+}$ indicator Oregon Green BAPTA-1 (OGB-1), and a clever scheme of delivering its AM ester form into thick tissues.^142^ Thanks to large 2P absorption cross-section of OGB-1 and other synthetic ${\mathrm{Ca}}^{2+}$ sensors, *in vivo* 2P ${\mathrm{Ca}}^{2+}$ imaging has been widely adopted within the neuroscience community and used for studying of neuronal circuits in healthy cerebral^143^^–^^147^ and cerebellar cortex,^148^ spinal cord,^149^ olfactory bulb,^150^^,^^151^ in glial cells,^152^^–^^154^ and in diseased brain.^155^^,^^156^

**Fig. 8:**
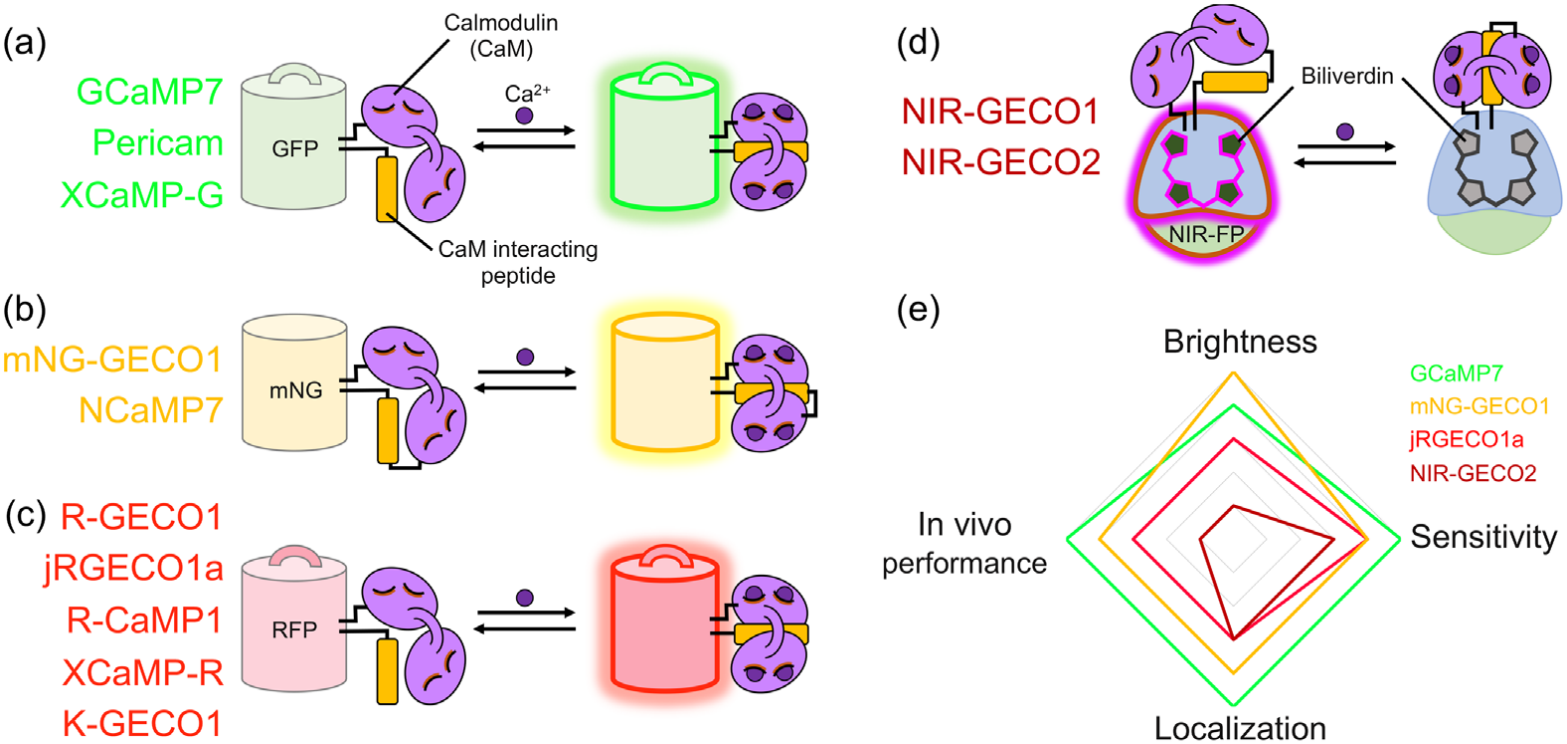
Overview of available intensiometric single FP-based GECIs. (a) Representative GFP-based GECIs.^159^^,^^163^^,^^164^ (b) Representative mNeonGreen-based GECIs.^165^^,^^166^ (c) Representative RFP-based GECIs.^164^^,^^167^^–^^169^ (d) Representative near infrared FP-based GECIs.^170^^,^^171^ (e) Qualitative assessment of various GECIs in terms of brightness, sensitivity, localization and *in vivo* performance.

Relative to synthetic indicator dyes, genetically encoded ${\mathrm{Ca}}^{2+}$ indicators (GECIs; a.k.a. ${\mathrm{Ca}}^{2+}$ biosensors) offer a number of advantages including genetic targeting to specific cell types, virus-mediated delivery, and the possibility of creating transgenic organisms that stably express the GECI. Just as with the field of synthetic ${\mathrm{Ca}}^{2+}$ indicator dyes, the field of GECIs was enabled by the work of Roger Y. Tsien and colleagues. Specifically, they reported the Förster resonance energy transfer (FRET)-based cameleon ${\mathrm{Ca}}^{2+}$ biosensor in 1997^157^ and the single GFP-based camgaroo ${\mathrm{Ca}}^{2+}$ biosensor in 1999.^158^ These were soon followed by the improved single FP-based pericam^159^ and GCaMP^160^ biosensors which were composed of a circularly permuted GFP fused to calmodulin (CaM) and a CaM-interacting peptide.^161^ The first GECI that was widely used to image neuronal activity in defined cell populations was GCaMP3,^162^ and seventh generation GCaMP variants have been reported.^163^ As the toolbox of genetically encoded ${\mathrm{Ca}}^{2+}$ biosensors continues to grow (Fig. 8), it is desirable to engineer biosensors with non-overlapping spectra to simultaneously monitor multiple circuit elements. Towards this goal, there are now a number of red FP-based^167^^–^^169^ and near-infrared FP-based^170^ ${\mathrm{Ca}}^{2+}$ biosensors available, though they do not offer the same outstanding levels of performance achievable with the latest generation of the highly optimized GCaMPs. However, these red-shifted probes offer the advantage of decreased scattering and absorption effects in tissue, thus allowing for deeper and more efficient imaging *in vivo*.

GECIs remain the workhorse of circuits-to-behavior neuroscience studies. The development and application of brighter and colorful ${\mathrm{Ca}}^{2+}$ biosensors along with other molecular tools will push the boundaries of exploration in neuroscience beyond the current limits.

### 3.2 Synthetic Voltage Sensors

Traditionally, neuronal electrical properties have been evaluated using intracellular electrophysiological recordings that are highly accurate but also terminal, invasive, low-throughput and labor intensive, and particularly difficult to perform in the brains of behaving mammals.

Synthetic sensors (dyes) provided an early impetus for probing transmembrane neuronal potential with optical imaging as an alternative to electrophysiological recordings. Nearly 50 years ago, experiments in squid axons revealed changes to the optical properties of dyes in response to membrane potential changes.^172^ Since that time, efforts to design fluorescent dyes that respond to biological membrane potential broadly fall into two different camps: slow-response dyes that display voltage-dependent partitioning within the membrane, and fast-response dyes whose optical properties are modulated by changing electric fields.^173^ Voltage-sensitive dyes are especially useful in model systems that lack ready tools for genetic manipulation and have been used extensively for mapping brain function in mammals and non-human primates.^174^

**Fig. 9:**
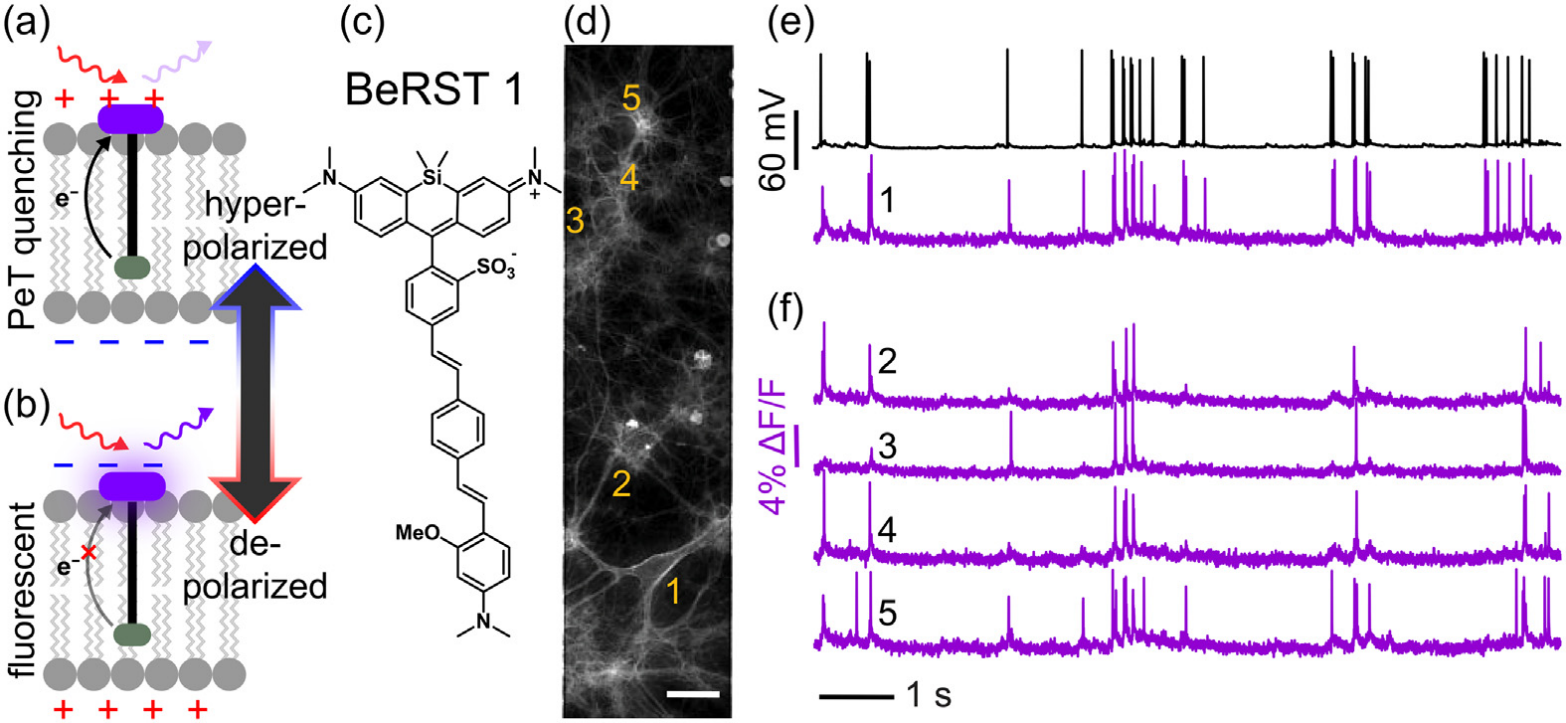
Voltage imaging with PeT sensors. (a)–(b) Mechanism of voltage sensing in PeT-based voltage-sensitive fluorophores. (c) Structure of a prototypical voltage-sensitive fluorophore, Berkeley Red Sensor of Transmembrane potential, BeRST 1. (d) Fluorescence image of cultured hippocampal neurons loaded with BeRST 1. Scale bar is 20  μm. (e)–(f) Example traces from simultaneous electrophysiological (black) and optical recording (magenta) of spontaneous voltage fluctuations from neuron 1 (e) and spontaneous activity from neurons 2 to 5 (f); the corresponding neurons are labeled in (d). Adapted from Walker et al.^178^ and Liu and Miller,^179^ © 2020 American Chemical Society.

More recently, voltage-sensitive fluorophores based on voltage-sensitive photoinduced electron transfer (PeT)^175^^,^^176^ have emerged as another avenue for synthetic voltage sensors^177^ (Fig. 9). Changes in membrane potential alter the rate of PeT within the donor-acceptor framework of the dyes. At hyperpolarized potentials, PeT from an electron-rich aniline donor is accelerated by the hyperpolarized potential, preventing emission of a photon from the fluorophore excited state, and quenching fluorescence [Fig. 9(a)]. At depolarized potentials, the inverted membrane potential inhibits PeT and the dye brightens [Fig. 9(b)].^179^ The newest PeT-based indicators compare favorably to genetically encoded indicators, in terms of speed, sensitivity, and brightness.^180^ Additionally, the positive-going, turn-on response to action potentials, coupled with compatibility with multiphoton excitation,^102^^,^^181^^,^^182^ make PeT-based synthetic voltage indicators an attractive method for imaging rapid membrane potential changes in intact brains. However, an outstanding problem is delivery of fluorescent dyes to specific cells.

In the future, a number of innovations could improve the utility of chemically-synthesized voltage indicators: improved or universal methods of delivery into intact brains; enhanced photostability for long-term imaging; larger multiphoton excitation cross-sections for use with 2P and 3P imaging; chemical-only cell targeting, without the requirement for genetic encoding, for example, by targeting native receptors;^183^^,^^184^ longer wavelength indicators for *in vivo* imaging;^185^^,^^186^ and, possibly, indicators that harness modalities other than light for noninvasive imaging.

### 3.3 Hybrid Voltage Sensors

Hybrid voltage sensors address the challenge of targeting fluorescent dyes to specific cell types. Multiple hybrid strategies have been explored. One attempt was using a “pro-drug” strategy in which a genetically encoded enzyme on the cell surface uncages a modified fluorescent voltage indicator.^187^^,^^188^ Fluorescein-based voltage-sensitive fluorophores can be adapted to this strategy,^189^^,^^190^ but the method has yet to be applied beyond cultured neurons. Alternatively, voltage-sensitive fluorophores can be tethered to neuronal surfaces with genetically-encoded, self-labeling enzymes to enable fast voltage imaging from defined cells and sub-cellular structures as was demonstrated in cultured hippocampal neurons^191^^–^^193^ or brain slice.^193^ Voltage indicators based on rhodamines can also be targeted in a similar fashion, allowing single-trial detection of action potentials in mouse brain slices.^193^

**Fig. 10:**
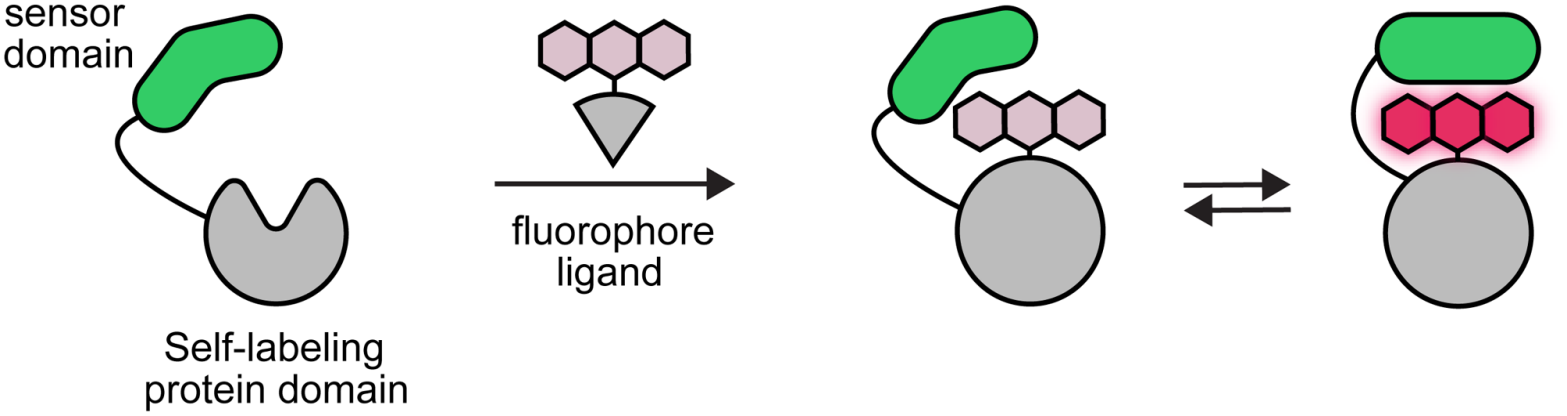
Schematic of chemigenetic probes. A biosensor domain is attached to a self-labeling domain that irreversibly binds a fluorophore ligand. A change in the biosensor domain alters the bound fluorophore properties allowing functional recording of biochemical signals.

Recent efforts have introduced another solution to the problem of cell-type-specific targeting through engineering “chemigenetic” probes that couple simple synthetic fluorophores acting as the reporter molecule with a sensor protein domain partner. In chemigenetic probes, a sensor protein domain is appended to a self-labeling protein domain^98^^,^^194^^,^^195^ such as HaloTag^196^^,^^197^ that irreversibly binds a synthetic fluorophore ligand (Fig. 10). This allows genetic encoding of the protein portion and uses simple and cell-permeable small-molecules, such as Janelia Fluor (JF) dyes,^25^^,^^198^ as reporter fluorophores.

Chemigenetic probes are engineered so that a change in the sensor protein domain alters the local environment around the dye and changes its fluorescence properties (Fig. 10). This approach yielded bright and photostable voltage and ${\mathrm{Ca}}^{2+}$ probes with highly tunable photophysical and chemical properties.^98^^,^^194^^,^^195^ Chemigenetic voltage probe Voltron^98^ increased photon yield from voltage imaging experiments to enable recording from many cells simultaneously. HaloCaMP and HASAP^195^ are bright far-red ${\mathrm{Ca}}^{2+}$ and voltage probes that push the useful imaging spectrum to longer wavelengths. These recent developments provide a template for the design of new fluorescent probes for different analytes and biochemical processes.

Further advances in synthesizing tunable far-red dyes, alongside innovative protein engineering for building protein scaffolds able to modulate dye fluorescence, promise to enable functional imaging deeper in tissue. Moreover, improved bioavailability of dyes will allow routine imaging in live animals. Another equally exciting avenue is to develop orthogonal self-labeling proteins like HaloTag that work well *in vivo* with corresponding bioavailable ligands for multicolor imaging.

### 3.4 Genetically Encoded Voltage Biosensors

**Fig. 11:**
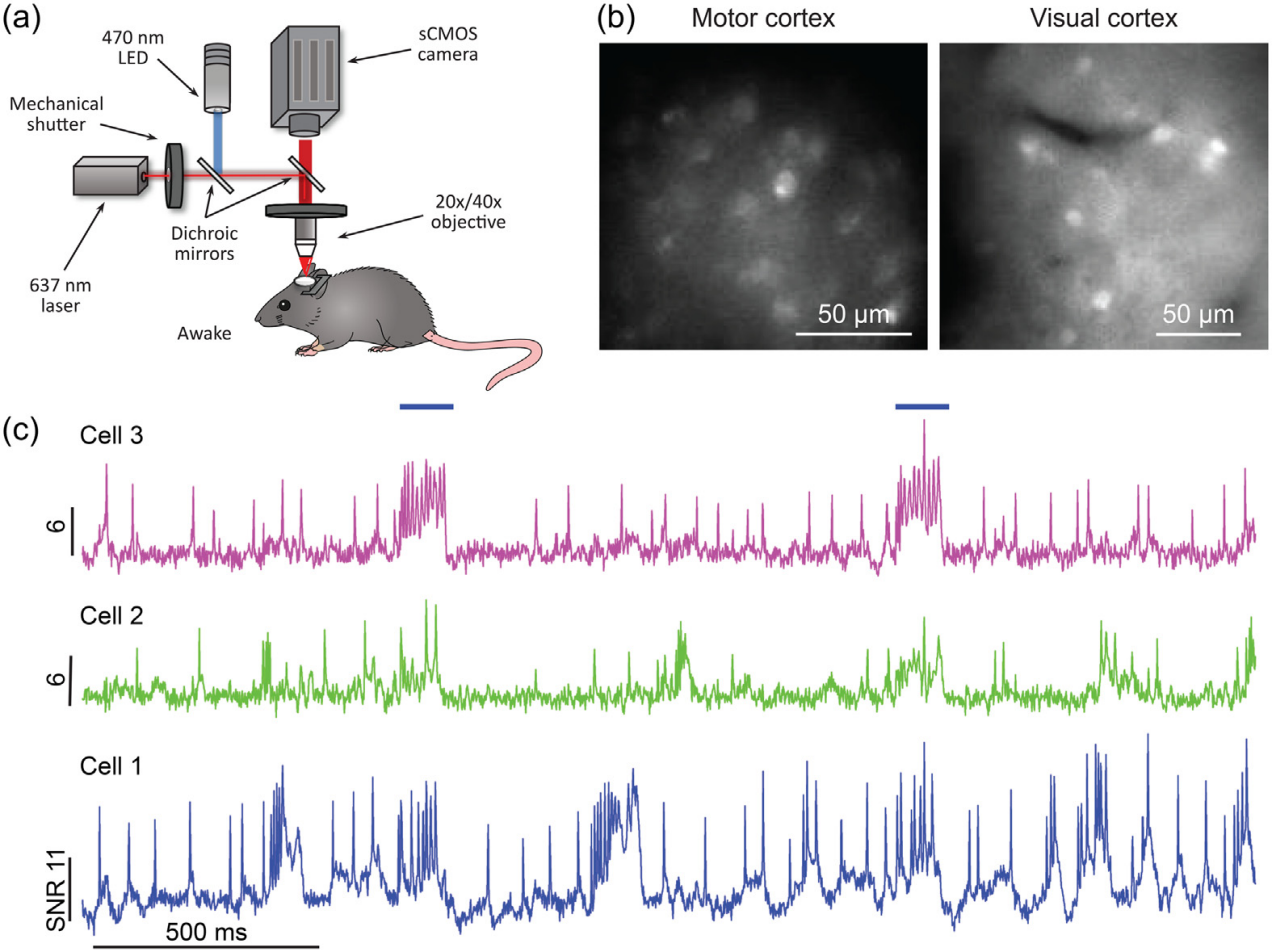
Single cell voltage imaging in awake mice using a wide-field imaging setup. (a) Experimental setup: awake mice were head-fixed under a wide-field microscope. (b) Representative SomArchon-expressing neurons visualized via EGFP fluorescence in cerebral cortex. (c) Voltage imaging time-courses from three representative neurons in hippocampus; horizontal bars denote optogenetic stimulation. Reproduced with permission from Piatkevich et al.^99^

Recent advances in the development of genetically encoded voltage indicators (GEVIs, a.k.a. voltage biosensors) have led to significant improvement in their optical properties offering recording of transmembrane voltage via fluorescence imaging without the need to deliver extrinsic (synthetic) sensors or fluorophores. In particular, a few recent GEVIs, including SomArchon, QuasAr3, ASAP3 and Ace2N have achieved sufficient sensitivity to capture individual action potentials in behaving mice.^99^^,^^100^^,^^199^^–^^201^ Figure 11 shows an example of voltage imaging of multiple cells simultaneously using SomArchon, which allows for the detection of individual action potentials and subthreshold membrane voltage fluctuations in behaving mice.^99^ Additionally, since SomArchon operates at the near-infrared wavelength, it can be used in conjunction with blue-light-activated optogenetic actuators for simultaneous voltage imaging and optogenetic control.

Several different types of GEVIs exist (for a recent comprehensive review, see Ref. 202). SomArchon and QuasAr3 are opsin-type GEVIs that use the voltage-dependent protonation of opsins as an optical readout. Indicators like Ace2N use an opsin to sense transmembrane potential, and changes in the protonation state of the opsin change the efficiency of energy transfer to a bright fluorescent protein (FP). ASAP3 is an FP fusion with a voltage sensitive protein domain which undergo conformational transitions upon transmembrane voltage fluctuations. These conformational changes are translated to the FP and change the fluorescence intensity of the FP.^203^^–^^209^ GEVIs have been used to tackle intriguing scientific questions, such as how spiking activity of specific neuron types is modulated during different behavioral processes and relate to subthreshold membrane voltage fluctuations and extracellularly measured local field potential dynamics.

With continued improvement in both voltage sensors’ molecular designs and optical instrumentation in the coming years, we expect that voltage imaging will be increasingly accessible to neuroscientists for high-speed kilohertz analysis of many individual neurons during behavior. Such capability will enable time-resolved analysis of transmembrane voltage, both action potentials and subthreshold membrane fluctuations, of distinct cell types and cell compartments in behavior and pathology. Finally, combining voltage imaging with other precision analysis technologies, such as cellular ${\mathrm{Ca}}^{2+}$ imaging and cell-specific proteomic and transcriptomic analysis, neuroscientists will be able to link the voltage dynamics of specific neurons and brain circuits to their cellular and biochemical states and tissue environment.

### 3.5 Genetically Encoded Biosensors for Neurotransmitters

**Fig. 12:**
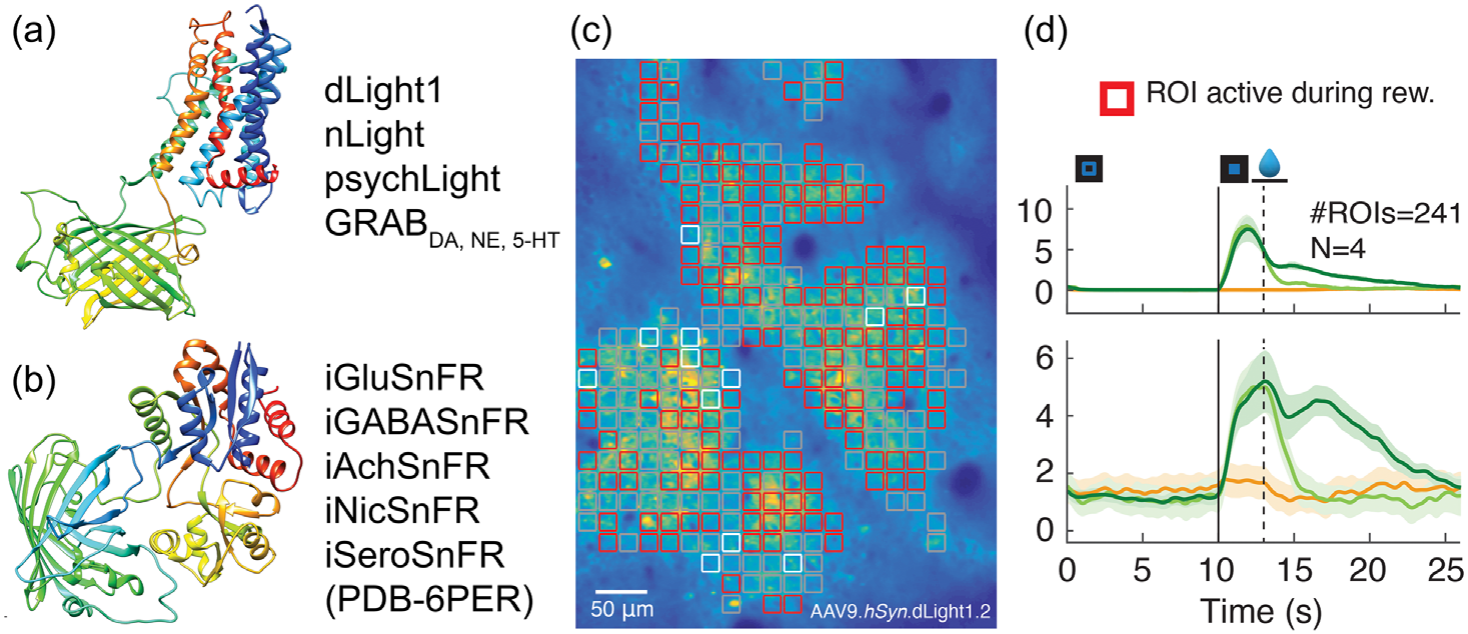
Genetically encoded sensors for neurotransmitters. (a)–(b) Design scaffolds for sensors based on cognate GPCR (a) or *E. coli* PBP (b). (c) 2P imaging of dopamine dynamics in the mouse cortex using dLight1.2 when mouse performs a visual-motor learning task. Heat map of dLight1.2 expression pattern in layer 2/3 of M1 cortex is overlaid with computationally defined regions of interest colored by the type of responses observed during the task. (d) Average task-induced dLight1.2 transients (bottom) and mouse running velocity (top) aligned to trial onset (0 s). For details see Ref. 210.

The development and refinement of genetically encoded ${\mathrm{Ca}}^{2+}$ and voltage indicators have paved the way for the development of genetically encoded biosensors for neurotransmitters, neuromodulators, and neuropeptides. Such biosensors are categorized by two scaffolds: microbial periplasmic binding protein (PBP) and G-protein coupled receptors (GPCR) [Figs. 12(a)–12(b)].

Microbial PBPs form a large protein superfamily that bind numerous classes of small molecules and peptides. Ligand binding in PBPs induces a large Venus flytrap-like conformational change, which is highly conserved [Fig. 12(a)]. These unique features have been used to develop a toolkit of highly sensitive sensors for other neurochemicals, including GABA (iGABASnFr), ATP (iATPSnFR), acetylcholine (iAchSnFR) and nicotine (iNicSnFr).^211^^–^^214^ However, there are several analytes for which bacterial PBPs do not exist. Recently, a PBP-based biosensor iSeroSnFr for serotonin has been engineered using machine-learning-guided evolution of an existing PBP-based biosensor, iAchSnFr,^211^ to redesign its binding pocket to report serotonin release at physiological concentrations.^215^

As an alternative to PBP-based biosensors, biosensors for neuromodulators have been developed by fusing GPCRs with fluorescent proteins, leveraging on ligand specificity, affinity, and binding kinetics that has evolved in the GPCRs. The first generation of GPCR-sensors were FRET-based.^216^^–^^219^ However, use of these biosensors *in vivo* has been limited due to low dynamic range and sensitivity. GPCRs have seven transmembrane (TM) alpha helices, where the largest conformational change upon activation is thought to occur for TM domains 5 to 7.^220^ Thus far, circularly permuted FPs (cpFPs) have been inserted in the intracellular loop 3 (IL3) domain of GPCRs, which bridges TM5 and TM6, to detect this conformational change upon ligand binding [Fig. 12(b)]. Using this versatile strategy, the Light and GRAB sensor family, consisting of dopamine, norepinephrine, and serotonin biosensors, has been developed and applied for *in vivo* recording of neuromodulator dynamics^210^^,^^221^^–^^226^ [Fig. 12(c)]. In addition, red-shifted color variants of dLight1 was engineered for multiplexed neurochemical detection (Patriarchi et al., 2020). GRAB biosensors have also been expanded to acetylcholine, adenosine and more recently endocannabinoid.^224^^–^^226^

The intrinsic properties of these neurotransmitter biosensors demand further iterative optimization to be broadly applied to study spatial and temporal patterns of activity in synaptically connected neuronal circuits. Computational modeling would provide theoretic guidance for future optimization. In addition, a high-throughput screening system using fluorescence-activating cell sorting (FACS) combined with image-based screening or the well-known CRISPR/Cas9 system is a pressing need to speed up the process of optimization. Extending the sensitivity, specificity, and color palette of these biosensors will continue to create rich opportunities for studies aimed at understanding the role of specific neurotransmitters in calculations performed by neuronal networks.

### 3.6 Molecular Probes for Cell Metabolism

Brain metabolism is highly dynamic, fluctuating with the local level of neuronal activity and with consequent energy demands. Substantial amounts of ATP are required to support the ion gradients that serve as the batteries for electrical activity, and energy is also needed to support neurotransmitter release and recycling. This energy is provided mainly by the metabolism of glucose, although other fuel molecules can also contribute. Individual brain cells also can differ substantially in their metabolic demands and responses: not only may there be differences between different classes of neurons, but various glial cells—astrocytes (the focus of Sec. 3.7) and oligodendrocytes—may play specific roles in energy metabolism.

**Fig. 13:**
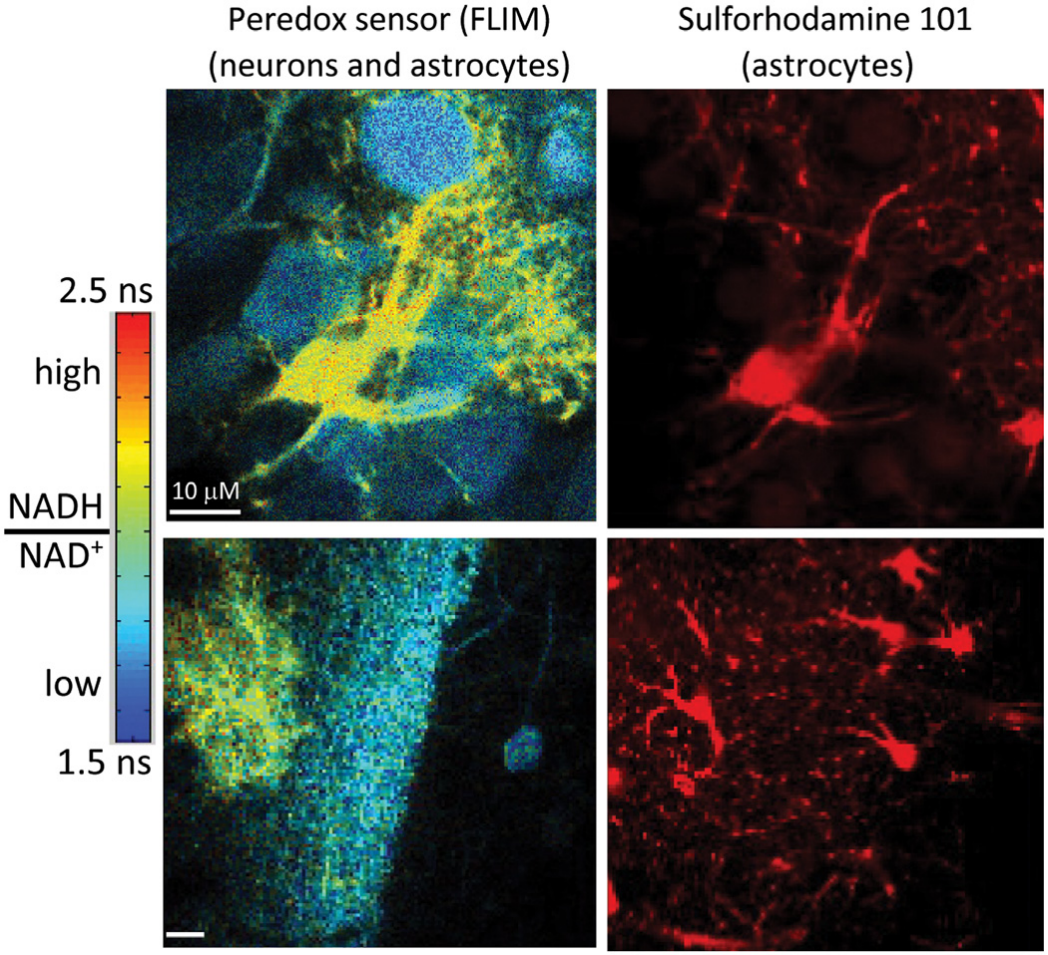
Metabolic imaging of the Peredox biosensor of $\mathrm{NADH}/{\mathrm{NAD}}^{+}$ redox, expressed in neurons and astrocytes of acute hippocampal slice. Left: pseudocolor images of the biosensor, which reports high $\mathrm{NADH}/{\mathrm{NAD}}^{+}$ ratio as increased fluorescence lifetime. Right: counterstain with sulforhodamine 101 marks astrocytes specifically and shows that they are the cells with elevated $\mathrm{NADH}/{\mathrm{NAD}}^{+}$ levels in the left-hand images, while neurons have lower levels. Not all cells in the slice express the genetically encoded biosensor, which was introduced with a viral vector. See further discussion of astrocytes in Sec. 3.7.

Because the metabolic responses are rapid (a few seconds to a few minutes) and can be localized to individual cells and subcellular compartments, imaging of fluorescent biosensors can be the ideal approach to investigating how cellular brain metabolism works. Genetically encoded biosensors have been developed for glucose^227^^–^^230^ and ATP;^231^^–^^233^ for the key electron-carrying cofactors $\mathrm{NADH}/{\mathrm{NAD}}^{+}$ (Fig. 13) and NADPH/NADP^234^^–^^237^ and for the metabolic intermediates pyruvate^238^ and lactate.^239^ These have been imaged both *in vitro* and *in vivo* to elucidate metabolic responses in neurons and astrocytes,^240^^,^^241^ though some features remain quite controversial.

A particular challenge for using metabolic biosensors is that quantitative readouts are essential for interpretation; unlike the ubiquitous ${\mathrm{Ca}}^{2+}$ biosensors used to monitor neuronal activity, for which the temporal pattern itself is informative, metabolite levels must be compared between different cells and different conditions, even when biosensor expression levels vary. Ratiometric imaging can be used to normalize for expression levels, but ratio imaging requires more spectral bandwidth and reduces the opportunity for multiplexing several biosensors; excitation-ratiometric measurements can be challenging for scanning microscopes; and single-FP ratiometric biosensors also tend to be quite pH sensitive.^120^ A valuable alternative is to use fluorescence lifetime imaging: the lifetime readout is independent of biosensor concentration, and rapid lifetime imaging is increasingly practical.^120^^,^^241^^,^^242^ Lifetime-based biosensors are slightly harder to develop, but the ability to multiplex and to get instrument-independent quantitation makes them a valuable target for biosensor development in the coming years.

### 3.7 Sensors for Astrocytes

Astrocytes are one of the major non-neuronal cell types in the mammalian central nervous system (CNS), comprising up to 20% of the human brain.^243^ Astrocytes are found throughout the CNS in non-overlapping domains.^244^ These cells possess a characteristic morphology, with a central cell body radiating numerous membranous processes, which extend out into the neuropil interacting with neurons, other glia, and blood vessels.^243^ Astrocytes are electrically silent, which meant that for many years they were dismissed as being merely parenchymal support cells – or “brain glue.” The development of new optical imaging tools and their application to astrocytes has forced a dramatic rethink of this preconception.^122^ Indeed, *in situ* imaging experiments, performed largely in acute tissue slices from rodents, have provided compelling evidence that astrocytes are key components and regulators of neuronal circuits.

Use of small organic ion-sensitive dyes and genetically encoded biosensors has demonstrated that astrocytes contribute to local synaptic homeostasis, through active buffering of ${\mathrm{Na}}^{+}$^245^ and $K^{+}$.^246^ Meanwhile, use of metabolic biosensors suggests that astrocytes also act as “power plants,” supplying lactate (a key energy substrate) to neurons.^240^ Astrocyte-specific expression of genetically encoded neurotransmitter biosensors has shown that these cells are well placed to sense local neuronal activity and neurotransmitter release;^247^ crucially, structural imaging of membrane-associated fluorescent proteins,^248^ and tagged glutamate transporters,^249^ suggests this activity can also trigger dynamic changes in astrocyte processes, which reciprocally modulate synaptic transmission. Such modifications may well be linked to transient increases in cytosolic ${\mathrm{Ca}}^{2+}$, which are also evoked by local neuronal activity, and which, under certain circumstances, are thought to trigger the release of neuroactive substances (gliotransmitters) from astrocytes that act to modify neuronal activity.^250^

**Fig. 14:**
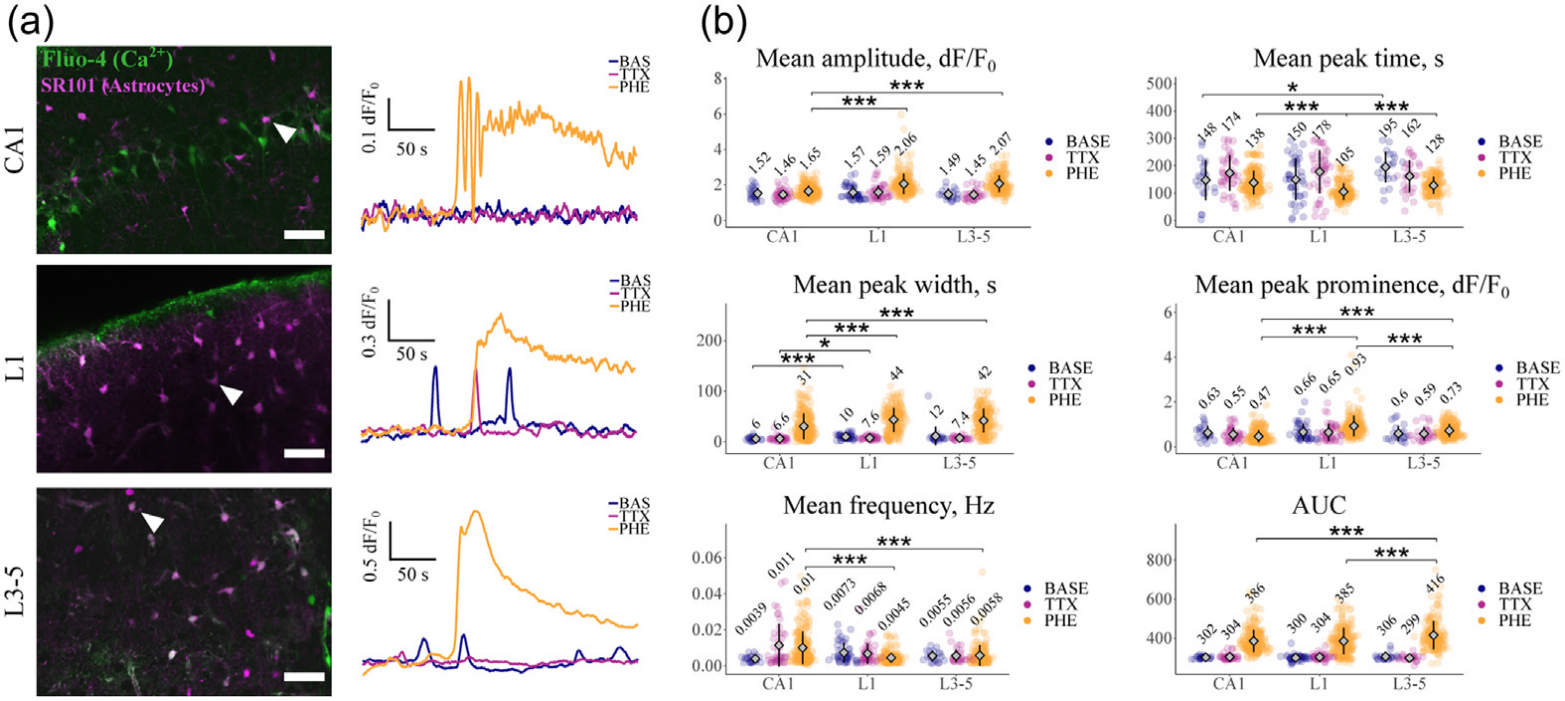
Astrocyte subpopulations in the mouse CNS show significant differences in ${\mathrm{Ca}}^{2+}$ signaling. ${\mathrm{Ca}}^{2+}$ transients in sulforhodamine101 (SR101)-labeled astrocytes were detected using Fluo-4. Measurements were made in mouse acute tissue slices containing cortical layer 1 (L1), cortical layers 3-5 (L3-5) and the hippocampal CA1 region (CA1). ${\mathrm{Ca}}^{2+}$ transients (expressed as fluorescence changes relative to baseline: $\mathrm{dF}/F_{0}$) were initially recorded under conditions of baseline activity (BASE) and after sequential application of tetrodotoxin (TTX) (to isolate astrocytes from neuronal activity) and tetrodotoxin plus the $\alpha_{1}$-adrenergic receptor agonist phenylephrine (PHE). (a) Representative astrocytes (arrowheads) from three brain areas and the ${\mathrm{Ca}}^{2+}$ transients recorded from these cells under the various experimental conditions. Scale bar, 50  μm. (b) Analysis of various transient parameters, recorded under identical experimental conditions. Numerical values are the calculated means for each condition. AUC: area under curve. Modified from Batiuk et al.^254^ *p ≤ 0.05, **p ≤ 0.01, ***p ≤ 0.001.

This multifaceted modulation of neurons and synapses by astrocytes suggests these cells provide an additional layer of information processing to that occurring in neurons, one which appears crucial to the modulation of animal behavior.^251^ However, the role of astrocytes is complicated by the recent discovery of astrocyte subtypes, which exist both between and within brain regions.^252^ These subtypes show differences at the molecular, anatomical and physiological levels, and are likely specialized to match and regulate local neuronal circuits^253^ (and see Fig. 14).

Future work will benefit greatly from genetic tools (e.g. new Cre- mouse lines or viral vector systems) that allow precise targeting of these astrocyte subtypes,^255^ including in gene knockout models. These will allow the contribution of astrocyte subtypes to local circuit function to be studied in great depth. Such work will also benefit from more specific methods for the acute and chronic manipulation of astrocyte activity,^256^^–^^258^ as well as an expanded/comprehensive palette of sensors, allowing the mechanistic basis of astrocyte function to be elucidated. Such a toolbox will, of necessity, include sensors for ions, receptor activation (particularly G protein-coupled receptors), metabolic functions and key intracellular signaling pathways (including aspects of structural plasticity and mRNA/protein trafficking).^259^^,^^260^

Ideally, such probes will allow high levels of multiplexing (single wavelength probes) and be optimized for *in vivo* use (good signal-to-noise ratio, sensitivity, dynamic range, stability in the cellular environment, resistance to photobleaching, lack of toxicity, etc.), as recent work has demonstrated that astrocyte function is strongly influenced by behavioral state.^261^ Probe development will go hand-in-hand with improvements in head-mounted camera technology for use in awake behaving animals (e.g., UCLA miniscope project) and silicon probe design (for the simultaneous measurement of neuronal activity).^262^

In summary, genetic manipulation of regional and subregional astrocyte genes, in combination with (*in vivo*) functional studies of astrocytes and their interactions with local neurons, will bring new insights into diversified astrocyte function and the consequences for control of local neuronal circuits and subsequent animal behavior. Such information may well provide novel insights into CNS diseases. It is likely studies of other CNS cell types, such as oligodendrocytes, microglia and pericytes will benefit from adopting similar strategies.

### 3.8 Probes for Oxygen Measurements by Phosphorescence Quenching

Tissue oxygen levels can be measured by the phosphorescence quenching method^263^^,^^264^ using molecular probes with controllable quenching parameters and defined bio-distributions. This approach relies on the ability of oxygen ($O_{2}$) to quench emission originating from the triplet excited states of probe molecules. Oxygen levels are derived from phosphorescence decay times, which are independent of the optical properties of the medium and local probe concentrations. “Protected” oxygen probes for truly quantitative imaging of oxygen *in vivo* consist of platinum (Pt) or palladium (Pd) porphyrins encapsulated inside hydrophobic dendrimers, coated at the periphery with hydrophilic polyethylene glycol (PEG) residues.^265^^–^^267^

**Fig. 15:**
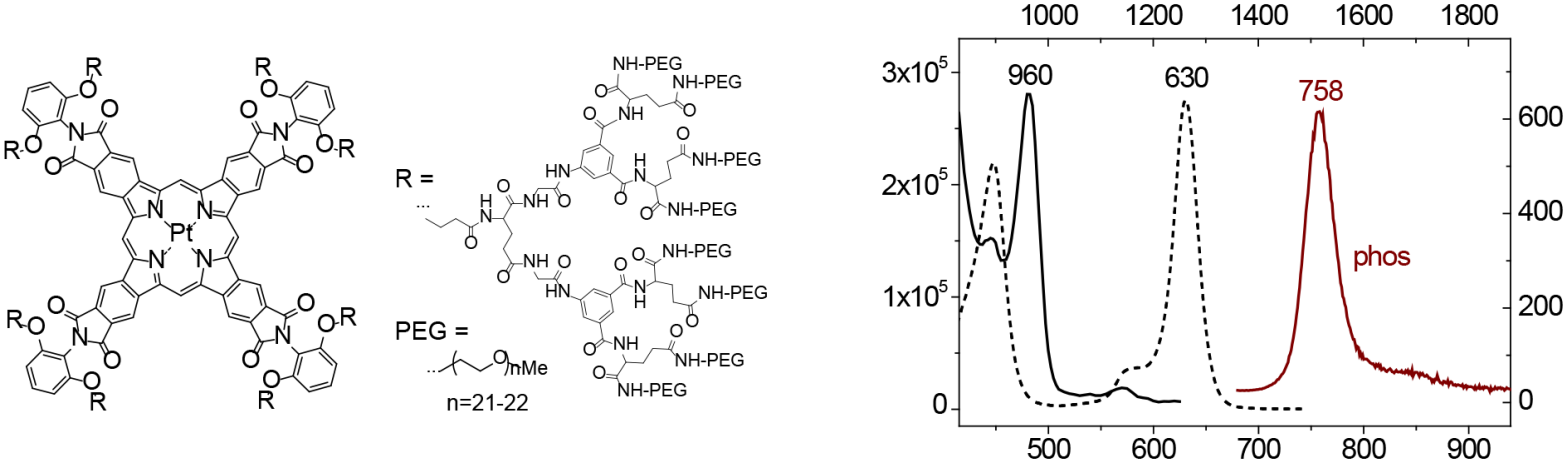
Structure, optical absorption spectra and basic photophysical properties of probe Oxyphor 2P. In the spectra (right): dotted black line, 1P absorption; solid black lin, 2P absorption; solid dark red line, phosphorescence.

Over the past decade, a special subset of phosphorescent probes has been developed specifically for 2P phosphorescence lifetime microscopy (2P PLIM or simply 2PLM) of oxygen.^124^ The 2PLM technique has become particularly popular in neuroimaging, enabling imaging of oxygen distributions and concentration gradients in live brain with micron-scale resolution.^268^^–^^274^ While being excellent linear absorbers, Pt and Pd porphyrins have vanishingly small 2P absorption cross-sections. In the original 2P oxygen probes (e.g., PtP-C343) the ability to absorb light by a 2P mechanism was boosted by supplementing the porphyrins with multiple 2P chromophores comprising 2P antennae.^124^^,^^275^^,^^276^ However, later a more potent probe was developed, known as Oxyphor 2P^125^ (Fig. 15), which relies on a single porphyrin whose 2P-active excited electronic states have been tuned by way of synthetic manipulations, rendering a chromophore with strong 2PA and exceptionally bright phosphorescence.^277^^,^^278^ Presently, Oxyphor 2P is the probe of choice for 2PLM as well as for other applications of phosphorescence-based oximetry (e.g. Ref. 279).

The next challenge in the phosphorescent probes development area is to combine oxygen imaging with sensing of other environmental analytes (e.g. temperature, proton gradients, metal ions, etc.), explore higher orders of non-linearity (e.g. 3PA), and create sensors that can be tagged selectively to particular types of tissues and cells.

### 3.9 Bioluminescent Tools for Imaging, Modulating, and Integrating

Photons are typically generated from a physical light source, but they can also be produced by a genetically encoded luciferase, an enzyme that oxidizes a substrate, a luciferin, thereby emitting bioluminescence. Bioluminescent imaging enables minimal background and no photobleaching as there is no need for an excitation light source. Bioluminescence can be repurposed beyond imaging and be utilized as a versatile genetically encoded light source for controlling optogenetic actuators.

**Fig. 16:**
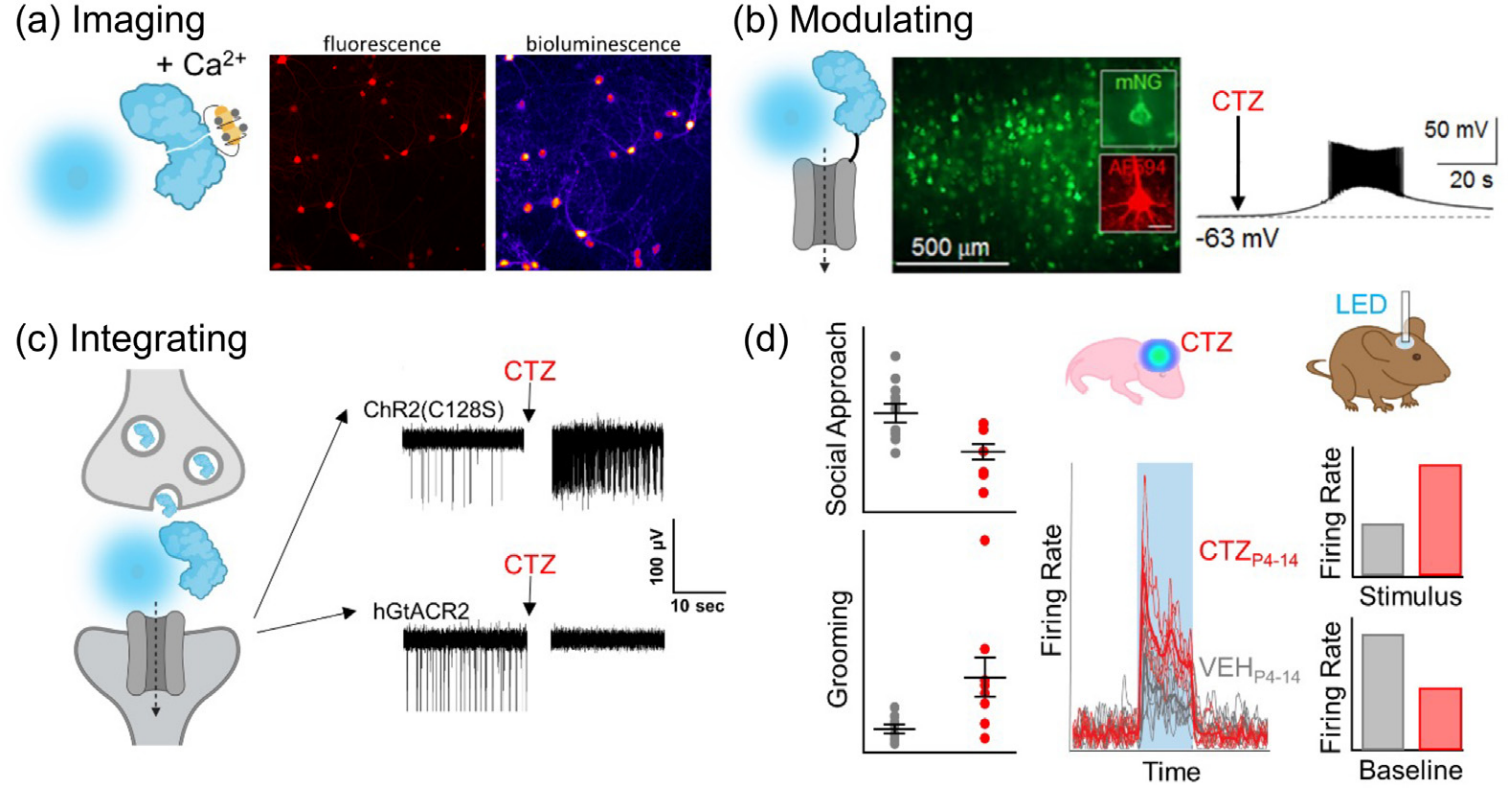
Bioluminescent tools for imaging, modulating, and integrating. (a)–(c) Luciferases are genetically encoded light emitters that can be employed for imaging neuronal activity when used as a ${\mathrm{Ca}}^{2+}$ dependent split molecule (a), for modulating neuronal activity when tethered to an optogenetic element (b), and for integrating neuronal activity by expressing the light emitter pre- and the light biosensor post-synaptically (c). (d) Example of experiments taking advantage of the bimodal feature of luminopsins: genetically targeted brain circuits were activated chemogenetically (luciferin) during a defined time window of postnatal development; the same neurons were interrogated in adult animals optogenetically (LED).

Core principles behind this platform technology are fusions of a light-emitting luciferase and a light-sensing photoreceptor such as opsins (channelrhodopsin, pumps),^280^^,^^281^ enzymes (adenylate cyclase, Cre recombinase),^128^^,^^282^ or transcription factors (CRY2/CIB, GAVPO).^128^^,^^283^ Expressing light-emitting luciferases and light-sensing proteins in pre- and postsynaptic neurons permit causal interrogation of synaptically connected populations through an “optical synapse” [Fig. 16(a)]. Engineered split luciferases allow for functional reconstitution, allowing light emission to be harnessed to activate light sensing proteins when ${\mathrm{Ca}}^{2+}$, voltage, neurotransmitters, and neuropeptides are detected or altered [Fig. 16(b)]. By making light itself activity dependent, optogenetic proteins can be modulated by luciferases serving as an integrator of biological processes [Fig. 16(c)].

Light emission from luciferases can be harnessed to control a range of biological processes by coupling bioluminescence with light-sensing proteins. Fusions of luciferase and opsin (luminopsins) have been used *in vivo* for improving motor deficits in a Parkinson’s disease mouse model,^284^ suppression of seizure activity,^285^ enhancing neuronal repair and functional recovery after ischemic stroke^286^ and spinal cord injury, modulating spatial and episodic short-term memory,^287^ studying the effects of developmental hyperexcitation on behavior and circuit activity in adulthood^129^ [Fig. 16(d)], and promoting axon regeneration after peripheral nerve injury.^288^

We can expect that this technology will benefit from discovery of new organisms with novel luciferase-luciferin combinations.^289^ Molecular engineering of luciferases towards brighter and faster indicators in conjunction with tuning light sensing proteins will enable imaging, modulation, and integration of neuronal activity in freely moving mice at increasing sensitivities. This will further be enabled by chemical engineering of synthetic luciferins extending the color palette and intensity of emitted light and advances in miniscopes.^290^ The trajectory of bioluminescent probes will allow imaging, controlling, and integrating neuronal activity with increasing sophistication.

### 3.10 Optogenetic Actuators

**Fig. 17:**
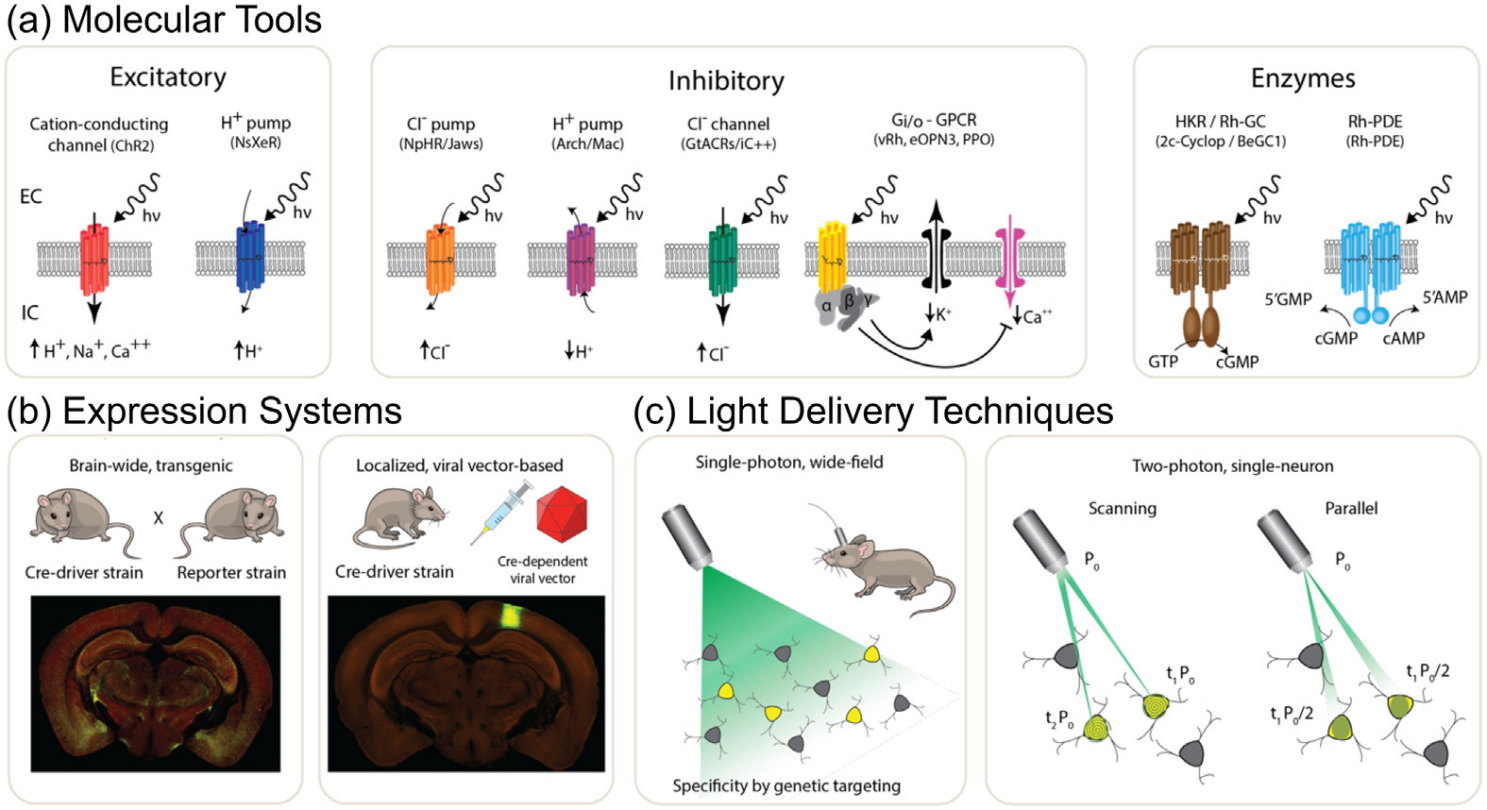
Optogenetic tools, genetic targeting and optical techniques. (a) Three major classes of rhodopsin-based optogenetic tools: excitatory pumps and channels (left); inhibitory opsins composed of chloride-conducting channelrhodopsins, proton and chloride pumps, and G-protein-coupled rhodopsins coupled to the inhibitory Gi/o pathway (center); enzyme-rhodopsins (right); shown are the histidine kinase group and the phosphodiesterase group. (b) Expression systems: two examples of gene delivery techniques used to express optogenetic tools in target neuron populations: crossing a recombinase driver mouse line with another that expresses a recombinase-dependent optogenetic actuator (left) or stereotactic injection of a viral vector encoding an optogenetic actuator. (c) Light-delivery techniques for optogenetics: (left) wide-field illumination of neurons expressing an optogenetic actuator through a chronically implanted multimode optical fiber or (right) 2P illumination of individual neurons using spiral-scanning of a diffraction limited spot or parallel illumination of extended shapes using wavefront shaping approaches. Images in panel (b) are from the Allen Institute for Brain Science (https://connectivity.brain-map.org/, experiments #741951571 and #288264301).

Optogenetic techniques have revolutionized neuroscience this past decade, offering an unprecedented toolkit for manipulating neuronal circuits with light.^130^ Driven by the goal of understanding the organization and function of the brain, optogenetics began with the discovery of the light-gated cation-conducting channelrhodopsins ChR1 and ChR2^291^^,^^292^ and the application of ChR2 for light-based excitation of mammalian neurons.^131^^,^^293^^,^^294^ This groundbreaking advance was followed by efforts of multiple groups to enhance and diversify the optogenetic toolbox.^295^^,^^296^ These efforts have culminated in the engineering of dozens of novel optogenetic tools for activation and silencing of neuronal activity, for modulation of G-protein signaling pathways and for direct modulation of second-messenger pathways [Fig. 17(a)]. Optogenetic tools have also been developed for light-based control over gene expression, synaptic release, and biochemical signaling pathways. While these have been recently reviewed elsewhere,^297^^–^^301^ we will focus here on the most commonly applied tools, which are based on microbial and vertebrate rhodopsin proteins. Common to all these novel tools is the utilization of naturally occurring light-sensitive proteins as actuators to drive cellular processes. The critical advance, and the main attraction of optogenetic tools for neuroscience research, is that these tools are all genetically encoded. This allows the use of genetic engineering techniques to direct the expression of optogenetic tools to precisely defined cell populations, through implementation of transgenic techniques and/or viral vector technology [Fig. 17(b)]. Selectivity is achieved via specific promoter or enhancer sequences that are exclusively expressed in the target cell populations. Cells expressing the light-sensitive protein respond to light in a manner defined by the properties of the specific protein used, while their non-expressing neighbors will remain unaltered. Together, the intersection of genetic specificity and the natural insensitivity of most brain tissue elements to light, render optogenetics a powerful technique for functional dissection of brain circuits in living animals. Optogenetics has been used for a wide range of experimental purposes, from functional dissection of neuronal circuit connectivity^302^ through investigation of the contribution of defined neuronal populations to highly specific behaviors^303^ to the development of light-driven interventions in animal models of neurological disorders^304^ and, most recently, treatment of human patients suffering from neurodegenerative disease.^305^

Optogenetic techniques require optical access to cells expressing optogenetic actuators, to allow light-based modulation of the targeted cells. While this is simple to achieve *in vitro* (Boyden et al., 2005), applying optogenetic manipulations *in vivo* requires technology for delivering light to the living brain. The first experiments using ChR2 in behaving rodents used multimode optical fibers coupled through cannulas that were chronically implanted or affixed to the skull.^295^^,^^306^ This arrangement allowed reversible connection of the optical fiber without the need to physically insert a light guide into brain tissue for each experiment. This simple technical approach has been the basis for numerous studies using 1P excitation [Fig. 17(c)] in a vast number of brain regions and neuronal circuits. However, one must consider that such manipulations are far from being able to accurately mimic the natural activity patterns expressed by populations of neurons in the unperturbed brain and might therefore lead to uninterpretable results due to “off-manifold” effects.^307^

In recent years, 2P techniques have been combined with highly refined optogenetic actuators to allow the control of single neurons^308^^–^^310^ and precisely defined multi-neuron ensembles [Fig. 17(c)], see Sec. 6). Using pulsed near-infrared lasers combined with spiral scanning or parallel illumination approaches,^311^^,^^312^ these developments have the potential to advance optogenetic techniques to the point that they can be utilized to “write in” information at the same level of spatial and temporal precision that the brain uses to encode information.^313^^–^^316^ These exciting developments will surely yield important insight into the natural “language” that neurons use to communicate, encode the external world, and execute behavior. These are long-standing questions in neuroscience. The “optogenetic revolution” has brought us to the exciting place where we can begin to address these questions with remarkable detail and precision.

#### 3.10.1 Optogenetic tools: light-sensitive proteins

Optogenetic tools are derived from a wide range of organisms, from archaea to fungi, algae, and animals. Genes encoding opsin proteins are widespread in nature, being found in all three domains of life and viruses.^317^^,^^318^ Metagenomics led to a revolution in the field of opsin research in the early 2000s and now both metagenomic and metatranscriptomic techniques are driving the rapid expansion of our collective understanding of the taxonomic and functional diversity of this family of proteins.^319^ While channelrhodopsins were the first to be applied as optogenetic tools,^131^ the current optogenetic toolbox contains tools based on light-gated ion pumps, cation and anion-conducting channels, G-protein coupled receptors and light-activated enzymes. The diversity of optogenetic tools allows for unprecedented flexibility in experimental design, but also demands an understanding of the basic working principles of the complexities and limitations associated with utilizing light-sensitive proteins.

#### 3.10.2 Light-activated ion pumps and channels

The most widely used opsins in optogenetics are cation-conducting and anion-conducting channelrhodopsins, termed CCRs and ACRs respectively. In the early days, the cation conducting Channelrhodopsin-2 from the alga *Chlamydomonas reinhardtii* (CrChR2 or just ChR2) and its first mutant (ChR2-H134R, a mutation introduced to enhance the amplitude of functional photocurrents) monopolized optogenetic investigation.^292^^,^^320^ ChR2 is a CCR, mostly permeable to protons but which also conducts other cations, including sodium but to a much lesser extent.^321^ Naturally occurring ACRs, first discovered in 2015, conduct a variety of anion species.^322^^,^^323^ Expressed exogenously in host cells, CCRs can act as photoactivatable depolarizing actuators while the function of ACRs is dependent on the chloride reversal potential. In many circumstances, the chloride concentration difference is such that ACR activation leads to hyperpolarization and neuronal inhibition. ACRs have been used extensively to silence adult mammalian neurons but they do have some limitations. Photoactivation of ACRs can lead to excitation in some cells (based on chloride reversal potential) and antidromic propagation when they are expressed in the axon.^324^^–^^326^ In contrast to channelrhodopsins, ion pumping rhodopsins have the advantages of high ion specificity, active transport of ions against the electrochemical gradient, and a wide variety of functions. Inward proton pumps (e.g. NsXeR) can be used to depolarize neurons,^327^ while outward proton (e.g. eArch3.0), outward sodium (e.g. eKR2), and inward chloride pumps (e.g. eNpHR3.0) hyperpolarize neurons and lead to inhibition.^328^^–^^331^ Unfortunately, in contrast to the robust ion flux generated by some channelrhodopsins, pumps are limited by a one-to-one photon-to-ion stoichiometry which can translate into weak photocurrents or photodamage induced by prolonged and intense illumination in efforts to overcompensate for these limitations.^332^ Additionally, excessive buildup of ions over time can damage cells, especially protons which can alter the intracellular or extracellular pH. These inconsistencies and limitations have motivated the community to attempt to identify new tools for neuronal inhibition. G-protein coupled receptors (GPCRs, discussed below) which engage G-protein signaling cascades or highly selective potassium channels (owing to the pronounced potassium gradient of neurons) would solve some of the issues with pumps and channels as hyperpolarizing actuators. While significant efforts to engineer a potassium channel from other channelrhodopsins enjoyed only partial success, naturally occurring potassium channels from *Hyphochytrium catenoides* were characterized recently and were termed Kalium Channel Rhodopsins (KCRs).^333^ Even though KCRs still need to be tested and validated for *in vivo* experiments, this new discovery has long been awaited by the optogenetics community and these actuators are a welcome addition to the toolbox.

#### 3.10.3 Opsin engineering and enhancement

Not long after the first optogenetics experiments were performed in the early 2000s, it became clear that the capabilities and diversity of opsins available to neuroscientists would need to increase substantially to match the scientific potential of the technique and overcome known and yet unforeseen roadblocks. In addition to the ongoing microbial ecological research to discover naturally occurring opsins, the engineering of opsins with desirable properties through targeted mutation and other means (such as fusions) became a fixture of the field. Efforts to engineer opsins with improved properties such as ion selectivity, spectral diversification, kinetics, light sensitivity, and ion conductance have seen success in recent years and have remained active areas of research. Spectral tuning is of particular interest, because red shifted light (i) penetrates more deeply into tissues, (ii) is less energetic than blue shifted light and thus causes less photodamage, and (iii) can be helpful to eliminate cross talk when opsins are paired with other tools such as fluorescent labels and voltage or ${\mathrm{Ca}}^{2+}$ indicators. A limited list of engineered opsins of interest include those developed for (1) altered ion selectivity: ChloCs,^334^ (2) spectral properties: ReaChR^335^ and Phobos,^336^ (3) light sensitivity: ChRmine,^314^ and (4) kinetics: Chronos,^337^ f-Chrimson.^338^ The selection of optogenetic actuators for specific experimental applications should be guided by their ionic selectivity, kinetics, action spectrum, and photocurrent amplitude.

#### 3.10.4 G-protein coupled rhodopsins and light-activated enzymes

Animal rhodopsins are GPCRs which catalyze GDP/GTP exchange through engagement of heterotrimeric G-proteins after light absorption.^317^ The utility of GPCRs in optogenetics research has advanced significantly in recent years with the implementation of bistable melanopsins and parapinopsins. Human and mouse melanopsin variants (hOpn4L, mOpn4L) from retinal ganglion cells can be activated and inactivated with blue light and yellow light, respectively, an advantage in optogenetics experiments where temporal control of activity is desired.^339^ While both opsins induce $G_{i/o}$ dependent inward rectifying $K^{+}$ (GIRK) currents and are highly light sensitive, mOpn4L exhibits superior on and off kinetics as well as less reduction in amplitude from sustained stimulation. However, these Opn4 variants both showed mixed activation of multiple G-protein pathways, leading to complex effects on neuronal physiology.^339^ Herlitze and colleagues later described Lamprey Parapinopsin (UVLamP or PPO) which has improved activation and deactivation kinetics compared to mOpn4L and reduced spectral overlap between the active and inactive states resulting in shorter light pulses required for full activation and deactivation.^340^ More recently, two new bistable opsins were characterized which couple selectively to the inhibitory $G_{i/o}$ signaling pathways and allow for efficient optogenetic silencing at presynaptic terminals. The targeting-enhanced mosquito homolog of encephalopsin, termed eOPN3, as well as the lamprey parapinopsin (PPO) were both shown to inhibit synaptic vesicle release through $G_{i/o}$ coupling.^341^^,^^342^ PPO allows switchable synaptic silencing, activated with blue-violet light, and deactivated with green light. eOPN3 exhibits a red-shifted action spectrum, high light sensitivity, and rapid activation. It returns to the inactive state spontaneously within minutes, making it an excellent inhibitory tool on the time scale from minutes to hours. Another notable member of the bistable GPCR-rhodopsin family, the zebrafish opsin Opn7b also couples to the $G_{i/o}$ pathway and is inactivated by light stimulation. Opn7b was applied as an “inverse optogenetic tool.” (Ref. 343).

The modulation of secondary messengers for the interrogation of cyclic nucleotide mediated cellular processes and signaling pathways has far reaching implications in neuroscience and biological systems in general and is an area where the spatiotemporal control afforded by optogenetics can provide a valuable advantage over pharmacological techniques.^344^ There are three classes of enzyme-rhodopsins that have so far been described in the literature: histidine kinase rhodopsin (HKR), rhodopsin guanylyl cyclase (Rh-GC), and rhodopsin phosphodiesterase (Rh-PDE).^345^^–^^347^ While the use of enzymerhodopsins for *in vivo* optogenetics research is not yet a staple, there are promising advances in the use of photoactive enzymes from flavoproteins. For example, the system comprising photoactive adenylyl cyclase from *Beggiatoa* (bPAC) and a cAMP-gated K+ channel from *Spriochaeta thermophila* (SthK) has been successfully applied to inhibit diverse neuron types in mice and Zebrafish embryos.^348^ More recently, a variant of bPAC was developed which exhibits lower dark activity (biPAC) and was then applied to investigate cAMP signaling in hypothalamic neurons and its impact on sexual behavior in mice.^349^

## 4 Imaging Brain Activity with One-Photon (1P) Excitation

Due to the rapidly expanding arsenal of fluorescent probes (discussed in Sec. 3), there is a growing need for technologies to enable fast and efficient imaging of these probes in brain tissue. The main advantage of 1P fluorescent methods is the efficiency of excitation quantified as the absorption cross-section: the probability of photon absorption by a fluorophore molecule. In comparison with multiphoton methods (Sec. 5), the probability of 1P absorption for virtually all of these probes is very high. Therefore, 1P excitation does not require pulsed lasers and can be achieved with relatively inexpensive diode-pumped solid-state laser (DPSS) lasers, LEDs, or even a filtered white light source such as a xenon or tungsten–halogen lamp.

With 1P excitation, one way to achieve fast, large-scale imaging is by using wide-field illumination of the entire brain surface and capturing the emitted fluorescent light with a camera. This “mesoscale” imaging (Sec. 4.1) is fast, because no scanning is involved, but lacks sectioning along the z-axis and has limited spatial resolution in the XY-plane due to light scattering. Therefore, mesoscale imaging is well suited for studies of cortical activity that do not require resolving single cells or cortical layers. Cell-type specificity can be achieved by genetic targeting of fluorescent probes.

1P fluorescence imaging technology has been miniaturized to enable measurements in freely behaving animals.^350^ These wearable “miniscopes” (Sec. 4.2) have been widely applied, in part due to a relatively simple, open-source, low-cost design.^351^^,^^352^

Laser scanning microscopy improves resolution at the price of imaging speed, because of the need to scan the excitation beam. Among different approaches, light-sheet microscopy^6^ has been successfully applied for *in vivo* imaging of brain activity in various organisms from semitransparent zebra fish embryos^353^ to mouse cortex^354^^,^^355^ (Sec. 4.3).

Optical imaging of deeper brain regions usually requires removal of the overlaying tissue or insertion of bulky lenses. As a compromise between the invasiveness of the measurement and the information content, fiber photometry has been used in many studies for detection of the fluorescence signal without spatial resolution. A number of recording channels can be increased using hundreds of splayed microfibers, each following a path of least resistance through tissue.^356^ More recently, a proof-of-principle demonstration of deep brain imaging has been achieved by scanning the excitation beam through an implanted multimodal optical fiber to form an image (Sec. 4.4). Stabilization of this minimally invasive technology will enable cellular and subcellular resolution beyond the penetration limit of multiphoton imaging (we discuss multiphoton imaging in Sec. 5).

### 4.1 Wide-Field Calcium Imaging

**Fig. 18:**
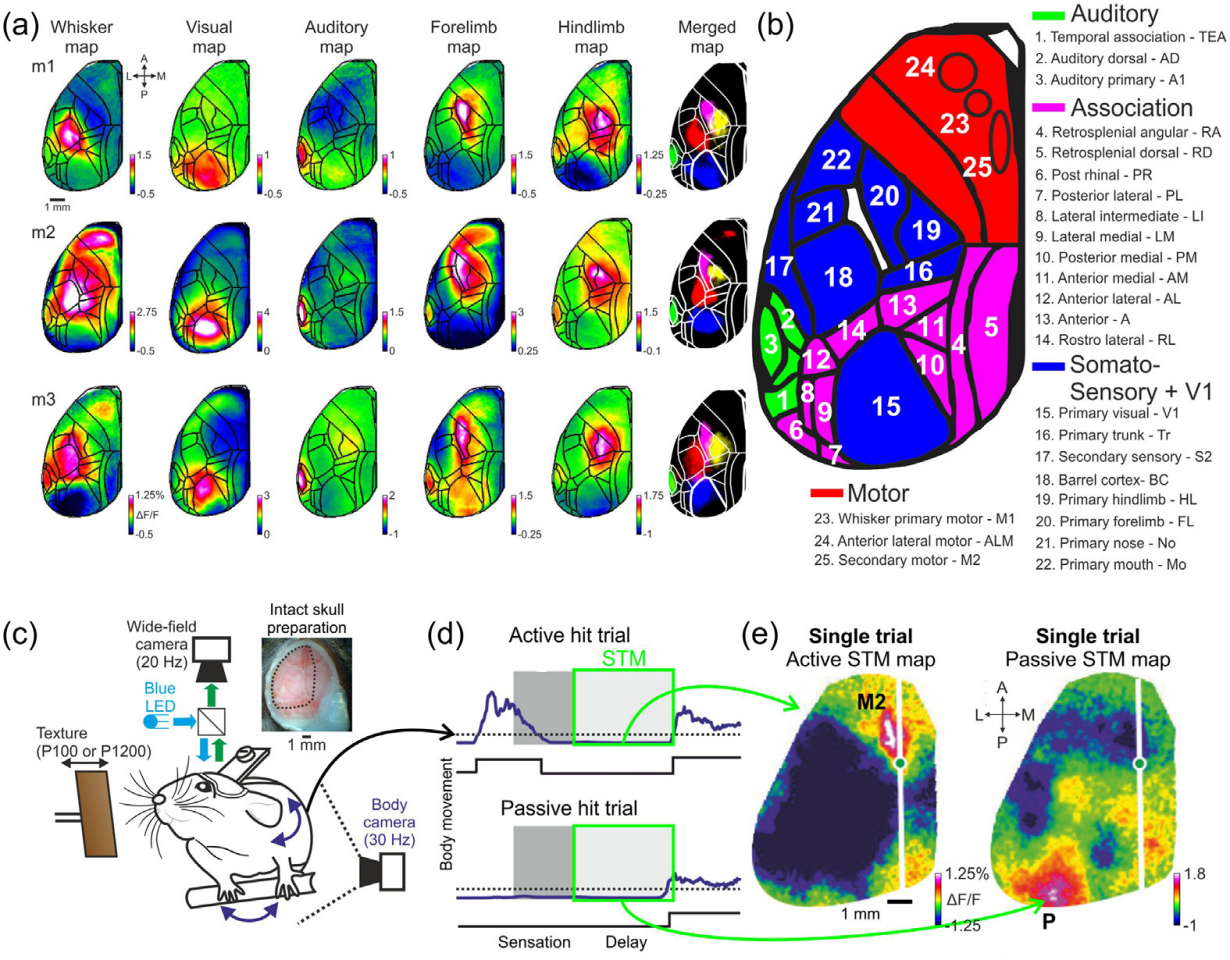
Mapping of brain areas and task-induced neuronal activity with mesoscale ${\mathrm{Ca}}^{2+}$ imaging. (a) Functional mapping of cortical areas. Example maps from three mice (m1, m2, m3) under anesthesia in response to different sensory stimuli: whisker, visual, auditory, forelimb, and hindlimb. Mean evoked activity map is shown for each stimulus type for each mouse. Color denotes normalized fluorescence (ΔF/F). The maps are registered onto the brain atlas (black lines; © 2004 Allen Institute for Brain Science. Allen Mouse Brain Atlas. Available from: http://mouse.brain-map.org/). Overlays of the 5 maps are shown on the right. (b) Parcellation of the dorsal cortical surface into 25 areas. Cortices are color-coded: auditory (green), association (pink), somatosensory + V1 (blue), and motor (red). (c) Behavioral and wide-field imaging setup in mice performing a whisker-dependent memory guided task. (d) Body movement vector in two example hit trials, one active and one passive. (e) Short term memory (STM) maps (averaged during the quiet delay period) for an active and passive trial showing frontal and posterior patches of activity, respectively. Color denotes normalized fluorescence (ΔF/F).

*In vivo* wide-field ${\mathrm{Ca}}^{2+}$ imaging in awake mice expressing GECIs (see Sec. 3.1) allows simultaneous imaging of many brain areas as mice perform complex behavioral tasks.^357^ Sensory integration, perception, memory and learning are just several examples of the cognitive functions that can be studied using this approach.^358^^–^^362^ In addition, mesoscale imaging can be used to characterize the organization of spontaneous cortical activity^363^^–^^366^ and functional mapping of cortical areas^358^^,^^367^ [Figs. 18(a)–18(b)].

Very much like a conventional epifluorescence microscope, a wide-field or mesoscale fluorescence imaging system consists of two major components: an excitation path delivering light to the brain surface and an emission path that projects an image of the light emitted by the fluorescent indicator onto a camera chip. The light source in most state-of-the art wide-field setups is either a DPSS laser or an LED matching the excitation peak of a fluorescent sensor. A corresponding emission filter in front of the camera blocks excitation light from reaching the camera. The camera is often the most expensive component in the imaging setup. Many imaging setups use sensitive and expensive sCMOS or EMCCD cameras, particularly when the fluorescence signal is weak due to sparse labelling or limited brightness of the sensor. Tandem lens systems, as initially described by Ratzlaff and Grinvald in 1991^368^ are often used to collect and project the emitted light onto the camera chip.^369^ Excitation light can enter in between the lens pair via a dichroic mirror. Alternatively, the brain surface can be illuminated obliquely via one or multiple lightguides positioned independently from the excitation path.^370^

The advantages of wide-field imaging are its large field of view (the entire dorsal cortical surface in the mouse) and the ability to image through the intact skull offering minimally invasive and stable conditions. In addition, a typical wide-field imaging setup is fairly inexpensive, appealing to many labs worldwide. The disadvantages are that 1P imaging is mainly sensitive to superficial cortical layers, and suffers from light scattering, particularly when imaging through the skull. In addition, the raw fluorescence signal can be contaminated by hemoglobin absorption. While many of commonly used fluorescent sensors for wide-field imaging are green (e.g., GCaMPs), hemoglobin strongly absorbs light in the blue-green spectrum. This leads to hemodynamic artifacts due to the hemodynamic response (an increase in the blood volume and decrease in blood oxygenation) associated with an increase in neuronal activity. Several methods have been developed to correct for this artifact. These methods require illumination with additional wavelengths, thereby adding further components along excitation and emission paths. One method uses estimated changes in concentration of oxy- and deoxyhemoglobin, derived from reflectance measurements at, e.g., 530 and 630 nm.^363^ Specifically for GCaMP imaging, one can use ${\mathrm{Ca}}^{2+}$-independent GCaMP fluorescence upon excitation at ∼405  nm.^371^ Due to the significant decrease in hemoglobin absorption at red wavelengths, hemodynamic artifacts are less prominent with red-shifted indicators, such as jRGECO1a, leading to relaxed requirement for hemodynamic correction of the collected fluorescence signals.^117^

An example of the insights gained from wide-field imaging is shown in Figs. 18(c)–18(e) adapted from Ref. 359. This study demonstrated that short-term memory was maintained in distinct cortical locations depending on the behavioral strategy of the mouse, either in a frontal area during an active strategy or a posterior area during a passive strategy.

Presently, wide-field imaging is being extended to specific neuronal populations via cell-type-specific targeting of GECIs.^164^ Further development of red-shifted GECIs and other types of indicators, such as voltage sensors, may enable deeper and faster imaging. Combining wide-field imaging with fiber photometry and/or electrophysiological recordings will permit simultaneously imaging/recording of neuronal activity from both cortical and subcortical areas. Finally, 1P fluorescence imaging is being implemented in freely moving mice (Sec. 4.2), opening the door to study more natural behaviors.

### 4.2 Wearable 1P Microscopes

Table-top microscopy systems, such as the one shown in Fig. 18 above, require head fixation of the animal under the microscope objective. This condition is incompatible with many behaviors. Wearable imaging systems have been developed to enable measurements in freely moving model organisms, from rodents to zebra finches and primates.^352^^,^^372^^,^^373^ These miniaturized microscopes (or “miniscopes”) exist in several implementations regarding imaging capabilities, size, and weight depending on experimental needs. Many of them are currently tethered (i.e., connect to the data acquisition system through flexible wires and, in some cases, fibers), allowing measurements during behavioral assays commonly used in research settings. Most of them rely on fluorescent indicators to provide the specificity and contrast necessary to study the cellular or molecular signaling of interest.

**Fig. 19:**
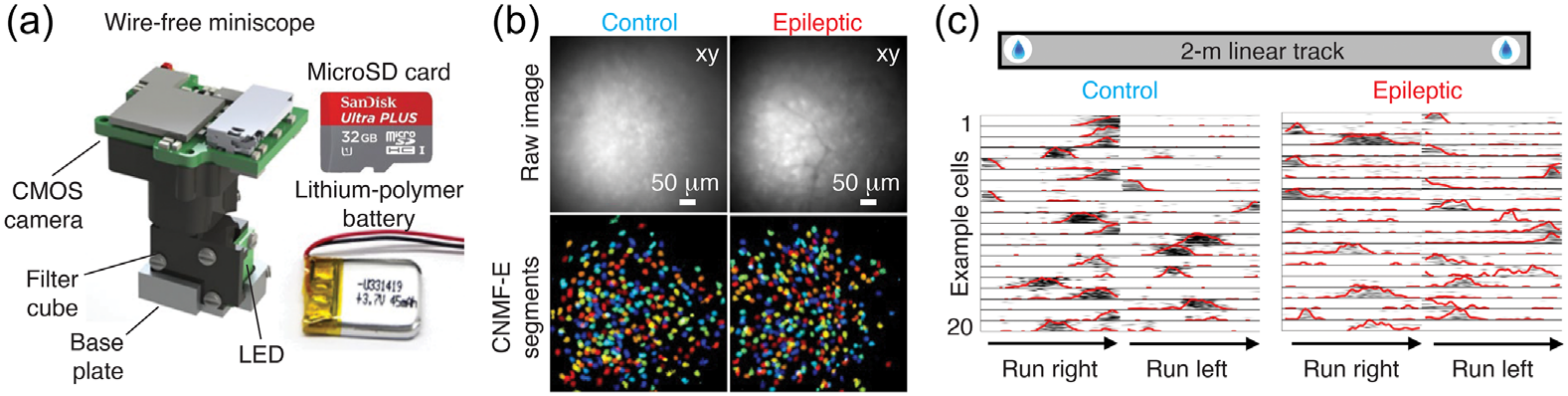
1P wearable microscopes for cellular-resolution imaging in freely behaving animals. (a) Example 1P miniscope for high-speed, cellular-resolution imaging in freely behaving mice. This implementation is wire-free and weighs 4-5 grams, including a 1.1-gram single-cell lithium-polymer battery. It allows recordings of ≥30  min duration at 20 Hz, 320 x 320 pixels resolution, x2 pixel subsampling across a 550  μm×550  μm FOV. Data is logged onto a MicroSD card for offline analysis. (b) Example raw images of GCaMP6f ${\mathrm{Ca}}^{2+}$ indicator-expressing (top) and computationally identified cells (bottom) in the dorsal hippocampus of control and epileptic mice. Imaging was performed using a tissue-implanted GRIN lens. (c) Spatially tuned activity pattern across 20 example cells from control and epileptic mice trained to run on a 2-m linear track for water reward. Adapted from Ref. 374 with permission from Nature Publishing Group.

1P miniscopes typically employ integrated LEDs or fiber-coupled DPSS lasers for fluorescence excitation and CMOS (complementary metal oxide semiconductor) image sensors for detection [Fig. 19(a)].^352^^,^^372^^,^^373^ They offer high frame rates (up to ∼60  Hz at full resolution) across large fields of view (from ∼0.6  mm up to 10 mm diameter). Spatial resolution varies with the field of view from few microns to tens of microns.^375^^,^^376^ One of the main drawbacks of 1P miniscopes is their potential out-of-focus signal contamination and limited optical depth penetration in densely labeled tissue (≤150  μm depending on tissue type). Deeper regions can be imaged using tissue-implanted relay optics, such as gradient index (GRIN) lenses [Figs. 19(b)–19(c)]. The relatively low equipment cost and ease of use of 1P miniscopes have facilitated their widespread adoption.

Wearable imaging systems have enabled biological studies difficult or unfeasible with their larger stationary counterparts (e.g., social interactions, sleep-wake transitions, seizure activity).^374^^,^^376^ They have provided insight into how genetically defined cell types encode external (e.g., sensory and motor) and internal (e.g., cortical state) information in various brain regions. More recently, miniscopes have also enabled real-time measurement of cellular activity in the spinal cord of behaving mice.^372^^,^^377^^–^^379^

In the future, we can expect these imaging systems to become even more powerful. Improved electronics and optics (e.g., new CMOS sensors, custom micro-optics, electronic focusing systems) will enhance 1P miniscopes’ ability to record at higher resolution, sensitivity, contrast, and speed, in multiple colors and across larger FOVs or tissue volumes.^352^^,^^372^^,^^378^^–^^380^ While initial tether-free designs exist [Fig. 19(a)], further improvements in onboard or wireless power and data transmission technology are needed to reduce overall device weight and boost recording duration at desired image resolutions and frame rates.

Complementary to these hardware improvements, new indicators (e.g., transmitter and voltage sensors) and computational tools [Fig. 19(b)] (see also Sec. 9) are likely to enable real-time analyses and interrogation of nervous system activity. These innovations will permit longitudinal investigation of various disease, treatment, or behavioral conditions best studied in freely moving animals.

### 4.3 Imaging in 3D with Light-Sheet Microscopy

The standard method of forming 3D images in intact, living specimens such as the brain is to scan a high numerical aperture (NA) point of light to different positions within a volume of interest. For instance, sequential raster scan of individual planes is often used in confocal microscopy to reconstruct a volume. However, this approach becomes increasingly challenging for high-speed imaging, both because scanners begin to exceed physical limits, and integration time per pixel becomes vanishingly small, nearing the fluorescence lifetime of many fluorophores.^381^ To overcome these limitations, it is possible to acquire data using parallel illumination of multiple regions at once. In multiphoton microscopy (Sec. 5), this approach has taken many forms, from multi-point scanning^382^^,^^383^ to extended axial range imaging.^384^^–^^386^ An embodiment of this approach using 1P excitation is light-sheet microscopy, which illuminates an entire plane within the sample, achieving optical sectioning by generating a focused image of this illuminated plane onto a camera detector. This approach greatly increases integration time per pixel compared to point scanning, while its selective illumination of only the plane being imaged makes light-sheet microscopy especially light-efficient.^381^

**Fig. 20:**
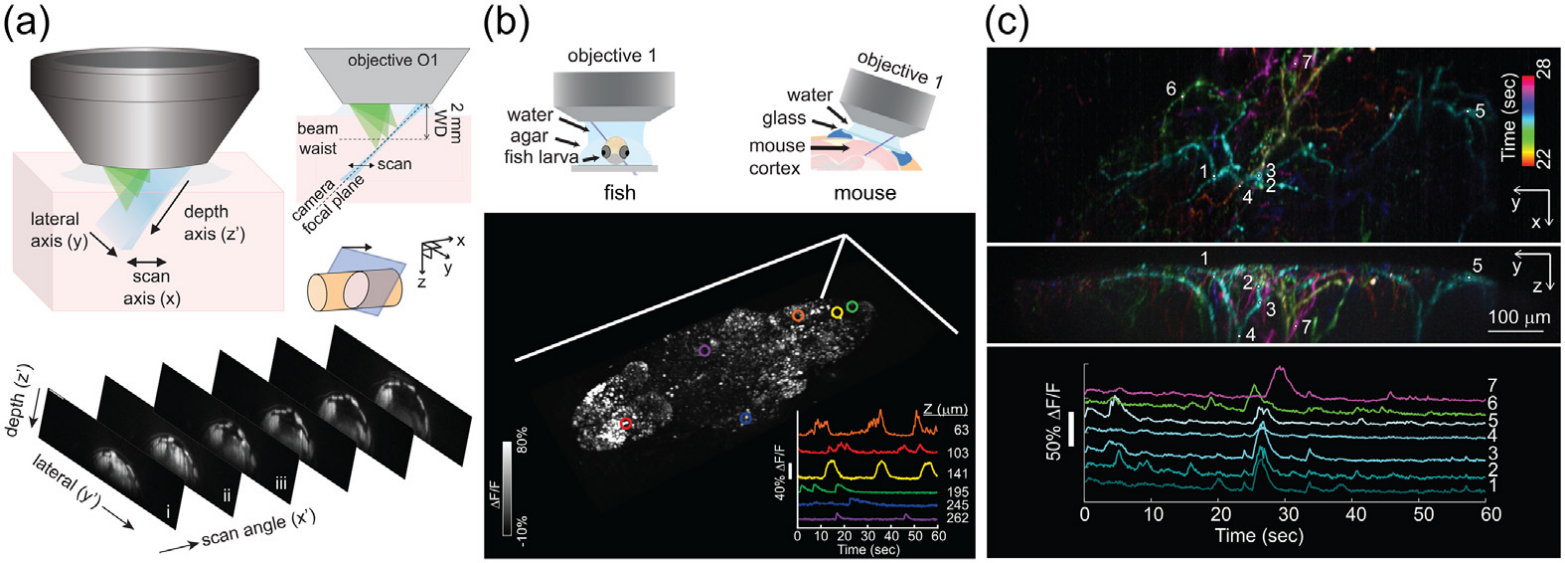
SCAPE microscopy: elements and application. (a) Top: SCAPE microscopy uses an oblique light sheet to illuminate the sample, and emitted light is collected by the same objective lens. SCAPE sweeps the oblique light sheet back and forth across the sample, de-scanning and rotating returning light to focus onto a stationary camera. Bottom: example images of a drosophila larva, captured as the light sheet scans. (b) Top: experimental imaging configurations to image neuronal activity in zebrafish larvae and awake, behaving mouse. Bottom: visualization of spontaneous neuronal GCaMP activity in the whole brain of a larval zebrafish. Volume rendering shows a maximum intensity projection over time with inset showing time-courses of 6 cells at 6 different depths in the brain. (c) Top and middle: spontaneous activity of apical dendrites of layer 5 neurons in whisker barrel cortex via GCaMP6f imaging in awake behaving mouse. Maximum intensity projection from the top (XY) and side (YZ) with colors denoting time of peak activity. Bottom: time-courses for the seven numbered regions of interest indicated above.

Light-sheet microscopy has traditionally been applied to imaging cleared tissues (Sec. 2.2), where generating a thin light sheet and focusing onto this light sheet with a second, orthogonal objective is relatively simple for samples up to the size of a whole mouse brain.^387^^,^^388^ Another application has been to image small organisms such as Drosophila embryos and zebrafish during early development, and also for functional imaging in the larval zebrafish brain and heart.^389^^,^^390^ However, the standard use of two orthogonal objective lenses in these light-sheet systems limits both sample size and geometry. 3D imaging speed is also limited by the need to either translate the sample through the light-sheet plane or scan the light sheet in synchrony with repositioning of the detection focal plane. An approach that overcomes these speed and geometry constraints is swept confocally aligned planar excitation (SCAPE) microscopy, a single-objective light-sheet approach, which uses a confocal de-scanning to enable an obliquely incident light sheet at the sample to be scanned from side to side, while the image of the oblique plane is de-scanned, rotated and mapped onto the face of a fast camera [Fig. 20(a)].^354^^,^^355^ The system’s single, stationary objective permits a wide range of intact samples to be imaged in an upright or inverted geometry and does not require restraint or physical translation of the sample. This approach permits 3D microscopy of a wide range of living organisms at speed of up to 300 volumes per second, leveraging the low phototoxicity associated with light-sheet excitation and high light efficiency to capture ${\mathrm{Ca}}^{2+}$ dynamics as well as 3D movement in behaving animals.

SCAPE microscopy has been applied to high-speed imaging of a range of different samples including proprioceptive neurons in crawling Drosophila larvae,^391^ whole-brain ${\mathrm{Ca}}^{2+}$ imaging in adult Drosophila,^392^ zebrafish larvae [Fig. 20(b)],^381^ ${\mathrm{Ca}}^{2+}$ and red blood cell dynamics in the beating hearts of zebrafish larvae,^355^^,^^393^ and neuronal activity in freely moving C. elegans worms.^355^ SCAPE is also effective for imaging the mouse nervous system, capturing activity in apical dendrites in layers 1–3 of the awake mouse cortex at 10 volumes per second [Fig. 20(c)],^7^^,^^354^^,^^381^^,^^394^ and profiling ${\mathrm{Ca}}^{2+}$ response properties of 10,000’s of olfactory sensory neurons in the intact mouse olfactory epithelium.^395^ All the prior examples utilized 1P excitation at 488 and 561 nm illumination. However, SCAPE and related light-sheet approaches are also suitable for 2P excitation which can enable deeper imaging into the living mammalian brain. In the future, 2P SCAPE will provide a competitive alternative to multiplexed 2P strategies outlined in Sec. 5. Efforts are also underway to implement SCAPE via an implanted GRIN lens enabling imaging of deep brain regions.

### 4.4 Deep Imaging Through Multimode Optical Fibers

GRIN lenses, prisms and fiber bundles have been successfully used to image deeper brain regions.^396^^–^^401^ However, insertion of these optical elements into the brain is invasive. One strategy to minimize this problem is imaging though hair-thin multimode fibers (MMFs). Light transport through MMFs is a complex, hardly predictable, yet deterministic process. It can be described by the transmission matrix (TM),^402^ a linear operator whose components can be experimentally measured by means of wavefront manipulation and interferometry.^403^ For this application, the TM can be conceptually thought of as a lookup table between the light “shape” entering and exiting the fiber.

**Fig. 21:**
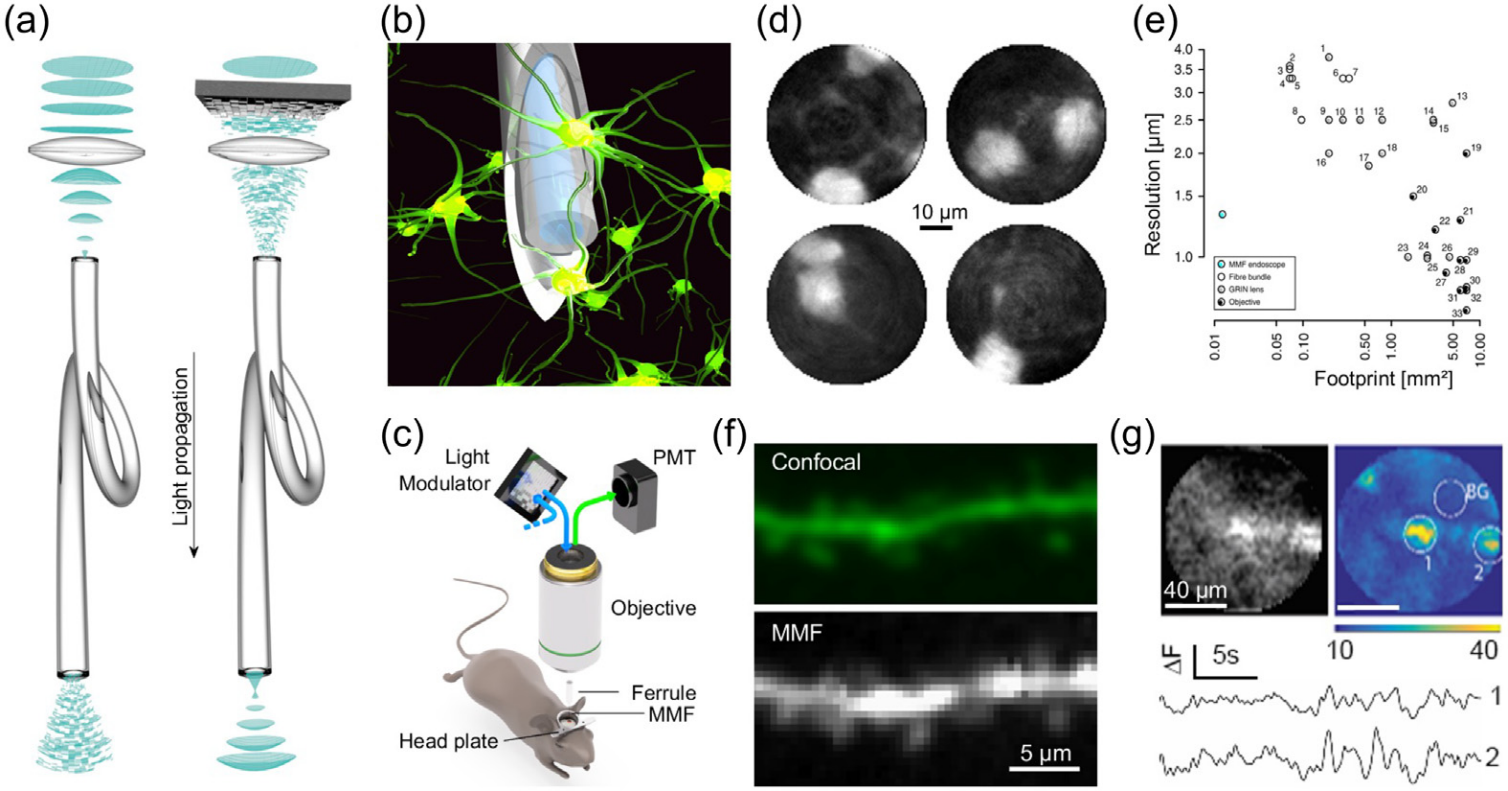
Deep brain imaging through an optical fiber. (a) Coherent light propagated through a MMF forms a speckle pattern at the output (left). Manipulation of the wavefront entering the MMF yields a desired optical field, e.g., a focal spot (right). (b)–(c) Experimental configuration for *in vivo* imaging. (d) Inhibitory neurons expressing tdTomato imaged *in vivo* (panel adapted from Ref. 403). (e) The trade-off between the fiber size and resolution. (f) Dendritic spines of a hippocampal neuron *ex vivo* resolved using a MMF (bottom). Comparison of the same field of view imaged with a confocal microscope (top). Panels (e) and (f) are adapted from Ref. 404. (g) *In vivo* neurons expressing GCaMP6f (left) and their activity visualized as standard deviation over time (right). Bottom traces show the change of signal in time after background (BG) subtraction from two regions of interest (1 and 2). Panel adapted from Ref. 405 © Optica.

Once available, the TM prescribes an optical field entering the MMF required to form the desired field distribution at the focal plane [Fig. 21(a)]. When applied to deep-tissue fluorescence imaging, the fiber is being advanced through the overlying tissue until it arrives at a subcortical brain region under investigation [Fig. 20(b)], which is then sequentially exposed to a series of illumination fields. These fields most commonly form diffraction-limited spots sequentially displayed across the focal plane, mimicking a laser scanning microscope. The emitted fluorescence is collected through the same fiber, spectrally separated, and recorded by a photodetector such as a photomultiplier tube (PMT) [Fig. 20(c)].

In context of neuroscience, holographic endoscopy has been used for structural imaging of neurons in cultures,^405^ acute brain slices^404^^,^^406^ and *in vivo*^403^^–^^405^ [Fig. 20(d)]. Among all other endoscopes, imaging with MMFs features the smallest probe footprint inducing tissue damage while maintaining subcellular resolution [Fig. 20(e), Ref. 404]. Even dendritic spines and axonal boutons can be resolved using MMFs with diameters ∼100  μm [Fig. 20(f), Ref. 404]. The first indication that spatially-resolved functional imaging of intracellular ${\mathrm{Ca}}^{2+}$ is possible to implement through the means of holographic endoscopy has been shown recently [Fig. 20(g), Ref. 405].

Using fibers with higher numerical apertures, imaging of custom-designed regions of interests and optimizing the animal models for indicator sparsity required in 1P imaging should ultimately open the door for minimally invasive imaging of neuronal activity anywhere in the brain. Moreover, high speed scanning of custom-designed trajectories along blood vessels containing a fluorescent indicator would allow imaging of blood flow similarly to a 2P technique pioneered by David Kleinfeld and collaborators.^407^ Importantly, the same probe could be used not only for imaging but also for spatially resolved optical stimulation of opsins (i.e., optogenetics).

All these imaging modalities funneled through a hair-thin optical probe would push the microscopic visual reach beyond the fundamental limits of optical far-field imaging technologies. The main challenge lies currently in transitioning this technology into awake animals and the realm of chronic experiments.

## 5 Imaging Brain Activity with Multiphoton Excitation

There are a few fundamental challenges when imaging brain activity. One challenge already discussed above (Sec. 4) is to be able to capture fast signaling events (1–100 ms) flowing rapidly between neurons. This challenge scales with the size of neuronal network under investigation. Another challenge is that brain tissue is often highly scattering, which limits the depth penetration of 1P microscopy methods. For example, in the mouse cortex, 1P voltage imaging can be used to measure spikes in cortical neurons with cell bodies located in layers I and II, i.e., less than ∼200  μm below the surface.^99^^,^^201^ Deeper penetration requires multiphoton imaging.

Here, we introduce a suite of multiphoton technologies that have been developed and/or significantly advanced in the last decade to penetrate deep into scattering tissue and cover a large field of view while retaining high resolution and speed. We start by highlighting a multi-region 2P microscopy design that has been developed to improve the imaging field of view (Sec. 5.1).^408^^–^^410^ These microscopes rely on very large objectives with high NA. The problem of multiregion imaging has also been addressed by optical systems with multiple arms and/or objectives.^411^^–^^413^

Next, we provide a brief overview of several strategies to increase the imaging speed through temporal and spatial multiplexing and “light sculpting,” i.e., engineering of the point spread function (PSF, a.k.a. the focal spot) (Sec. 5.2). This is followed by a more detailed discussion of two fast imaging strategies: discrete sampling of specific voxels of interest with acousto-optic lenses^414^ (Sec. 5.3) and multipass temporal multiplexing where the excitation laser pulses sequentially excite fluorescence in axially displaced voxels (Sec. 5.4).^415^

In 2P microscopy, excitation of fluorophore molecules by scattered photons results in the background signal leading to deterioration of signal to background ratio (SBR) with depth. This background can be reduced using two photons of different color derived from two laser beams that overlap only in the focal plane (Sec. 5.5). In 3P microscopy^416^ (Sec. 5.6), the probability of simultaneous absorption of 3 photons outside of the focal volume is very low, allowing deeper penetration. In addition, 3P excitation requires less energetic, red-shifted photons that scatter less in tissue.

The ability of multiphoton technologies to image the brain tissue deeper, clearer, and faster makes these tools well compatible with the “circuits-to-behavior” neuroscience studies. Ideally, these studies would be conducted in freely behaving animals. To this end, several miniaturized, portable multiphoton designs have been explored (Sec. 5.7).^417^^,^^418^ The major limitation for these miniaturization efforts is the fundamental tradeoff between the objective diameter, resolution, and field of view. To overcome this limitation, alternative strategies are being explored including microlens arrays^419^^,^^420^ and nanofabricated metalenses.^421^ In the meantime, however, performance of tabletop multiphoton microscopes remains unsurpassed.

### 5.1 Multi-Region 2-Photon (2P) Imaging

Many conventional 2P imaging systems have fields-of-view (FOVs) that are limited to $1\;{\mathrm{mm}}^{2}$ or less, and typically a single image plane is monitored per acquisition. However, neuronal circuitry can be distributed across many cubic millimeters, and capturing dynamics at this spatial scale, with a sub-second time resolution, can lead to novel insights into brain function. Thus, it is useful to pursue large-scale 2P imaging.

**Fig. 22:**
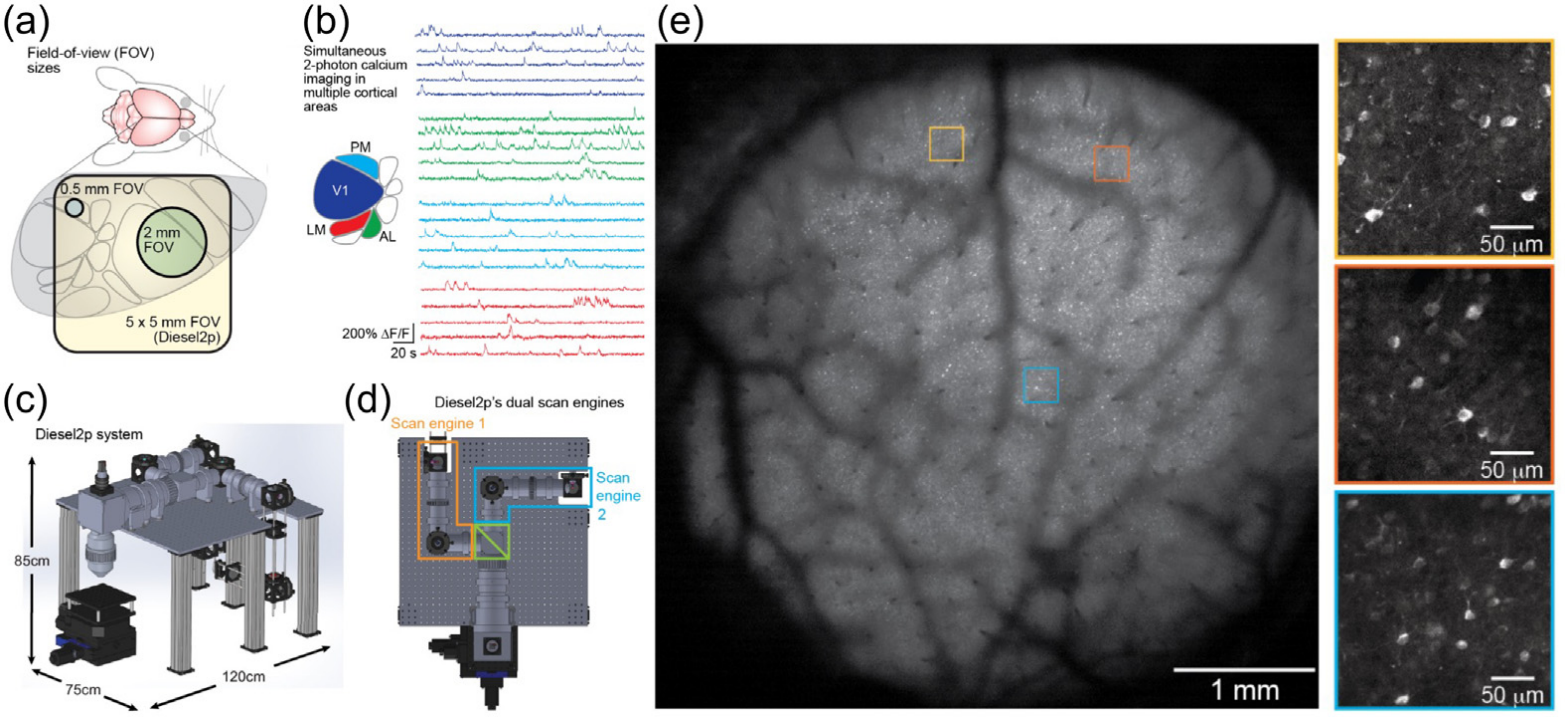
Large field-of-view, multiregion 2P imaging. (a) FOV available in conventional 2P systems are often confined to ∼0.5  mm, which is smaller than many cortical areas. Larger FOVs (2-mm diameter, or the 5 x 5 mm region of the Diesel2p system^410^ provide optical access to multiple cortical areas simultaneously. (b) Neuronal activity can be monitored across these FOVs through rapid beam steering and/or multiplexed beams. (c)–(d) The Diesel2p is a system that provides a large FOV (c) and dual scan engines coupled into a single objective (d) for independent multiplexed imaging and/or 2P optogenetic manipulations of neuronal activity. (e) With large FOV, 2P imaging systems, subcellular resolution is available across a large imaging volume, containing on the order of a million neurons. Adapted from Ref. 410.

Measuring neuronal activity across large FOVs ($∼10\;{\mathrm{mm}}^{2}$ or more) with subcellular resolution requires relatively large diameter optics, such as microscope objectives that are >50  mm wide.^408^^,^^422^^,^^423^ Objectives with moderate-to-high numerical apertures (>0.45  NA) and large FOVs (i.e., long focal lengths) provide access to an order of magnitude more pixels. However, the sampling time of the raster scan is unchanged, so scanning the full FOV results in frame rates that are too slow (>1  s per frame) to be useful for many applications. To this end, strategically choosing specific subregions within the large FOV and fast jumping between these subregions^408^^,^^410^^,^^422^ can be used to sample across large FOVs while still maintaining sub-second temporal resolution. This technique can be combined with temporal multiplexing^422^^,^^424^ and spatial multiplexing^409^ to achieve fast, simultaneous acquisition of multiple volumes (Fig. 22). An alternative approach to imaging in multiple regions simultaneously is to have multiple, small ROIs under separate, independently positioned objectives.^411^^,^^412^

Multiphoton imaging has been scaled up in the axial dimension as well. For example, one can temporally multiplex 2P imaging and 3P imaging (Sec. 5.6) to simultaneously image superficial and deeper neocortical layers.^425^ Many innovations in conventional 2P imaging have found application in multi-region 2P systems as well, including adaptive optics^410^ (Sec. 5.8), axially extended Bessel beam volume scanning^426^ (Sec. 5.2), and quad-region scanning.^427^

### 5.2 Sampling Strategies for Fast 2P Imaging

**Fig. 23:**
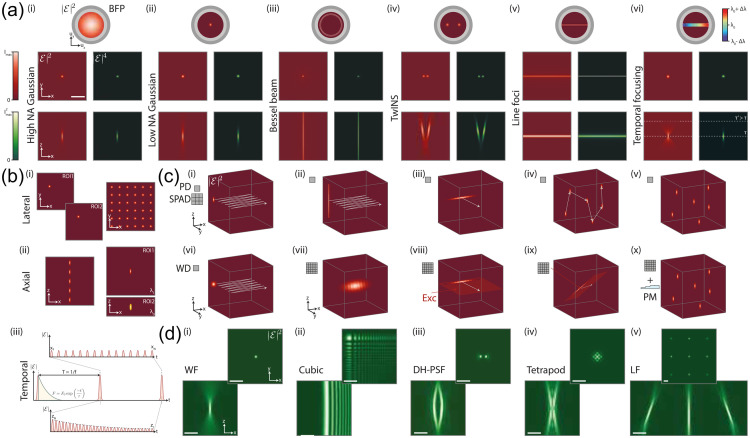
A summary of state-of-the-art approaches for fast 2P imaging. (a) Commonly used excitation modalities: (i) high NA gaussian beams,^428^ (ii) low NA gaussian beams,^429^ (iii) Bessel beams,^430^ (iv) stereoscopic low NA gaussian beams,^386^ (v) line foci for tomographic imaging^182^ and (vi) temporally focused, low NA Gaussian beams.^311^^,^^431^ Top row: simulated intensities of incident beams in the objective back focal plane (BFP) used to generate different excitation PSFs. Middle row: simulated excitation PSFs (red colormap) and the proportional 2P excited fluorescence (green colormap) generated in the focal plane (XY). Bottom row: same as middle but axial (xz) slices. The color bars and principal axes in (i) are applicable to all simulated data apart from (vi) where different colors are used to represent the different wavelengths constituting the ultrafast pulse with central wavelength $\lambda_{0}$ used for temporal focusing. τ refers to the pulse duration which is shortest at the focal plane where 2P excitation is maximized. Abbreviations: |E| refers to the amplitude of the electric field; ${\left|E\right|}^{2}$, the intensity, and ${\left|E\right|}^{4}$, the proportional 2P fluorescence; NA, numerical aperture; TwINS, 2P imaging of neurons using stereoscopy. (b) A summary of different implementations of (i) lateral, (ii) axial, and (iii) temporal multiplexing that have been applied to fast 2P imaging. (i) Left: the incident beam is divided into two beamlets each directed to simultaneously image different regions of interest (ROI).^432^ Right: the excitation field is divided into multiple laterally separated spots which sample different portions of the same field of view.^433^ (ii) Multiple planes throughout a volume can be imaged simultaneously using axially multiplexed beams^432^ or by directing beamlets with different wavelengths to different sample depths.^424^ (iii) Since the lifetime of common fluorophores is shorter (shown, not to scale) than the separation between subsequent pulses generated by suitable mode-locked lasers, each pulse can be divided into multiple beamlets, which can be laterally (upper) or axially (lower) displaced to perform fast 2P imaging.^415^^,^^434^^,^^435^ (c) Sequential and parallel acquisition modes used for fast 2P imaging. (i)–(v) Methods based on single pixel detection: (i),^428^ (ii),^430^ (iii),^182^ (iv),^436^ and (v).^437^ (xii)–(x) Common parallel excitation methods combined with camera detection: (vii),^438^^,^^439^ (viii),^429^ and (ix).^440^ Abbreviations: PD, point detector; SPAD, single-photon avalanche diode array. (d) (i) Simulated widefield PSF and detection PSFs used for extended depth of field imaging: (ii),^441^ (iii),^383^ (iv),^442^ and (v).^443^ Abbreviations: PM, phase mask; WF, widefield; DH, double helix PSF; LF, light field.

Conventional 2P microscopy is based on raster scanning of a near-infrared laser beam focused into a “point” (diffraction-limited) PSF. However, as previously described, sequential scanning of this PSF throughout large (${\mathrm{mm}}^{3}$) volumes is too slow to image neuronal activity, which occurs on the timescale of milliseconds. Figure 23 summarizes various approaches for fast 2P imaging with large FOVs that have been proposed in recent years. Among them is the multi-region microscopy discussed in Sec. 5.1. Another (not mutually exclusive) approach is “sculpting” the PSF in space and time in order to increase the imaging speed (or to image larger volumes whilst maintaining acquisition rates).

Diverse photonic technologies^354^^,^^444^^–^^452^ have increased the rate of sequential scanning, and volumetric imaging rates have also been increased by converting lateral scanning of a patterned substrate in space into axial displacements.^453^^,^^454^ Some techniques aim to reduce the pixel dwell time of 2P microscopy to its fundamental limit (determined by the excited state fluorophore lifetime) by dividing high-energy ultrafast laser pulses into beamlets that sequentially excite fluorescence in different voxels.^415^^,^^424^^,^^434^^,^^435^ The temporal resolution of 2P microscopy has also been improved by optimizing the scan trajectory^450^ or by discretely sampling voxels distributed throughout the region of interest.^100^^,^^436^^,^^455^

An alternative approach for fast 2P imaging is to sculpt the excitation PSF and increase the instantaneous volume of excitation to reduce the total number of sequential measurements. This includes line scanning,^182^ and axially elongated excitation PSFs such as Bessel beams which are scanned in the directions orthogonal to their elongation.^426^^,^^430^^,^^456^^,^^457^ The acquisition of stereoscopic (tomographic) information is necessary in the case of elongated foci and densely labelled samples.^182^^,^^386^ Illumination with spatial and temporal focusing of femtosecond pulses has also been used to increase frame rates.^431^^,^^438^^,^^439^ Another subclass of parallel methods spatially multiplex the excitation beam, project multiple foci in 3D, and record fluorescence using 1D^425^^,^^432^^,^^437^^,^^449^ or 2D detectors.^383^^,^^433^^,^^441^^,^^458^^–^^460^

### 5.3 Two-Photon Imaging in 3D with an Acousto-Optic Lens

For many applications, such as imaging ${\mathrm{Ca}}^{2+}$ transients in neuronal cell bodies, specific dendrites, axons, or synapses, the ideal sampling strategy would not be a complete coverage of a volume, but rather a set of regions of interest (ROIs) strategically positioned to overlap with locations of interest. This can be achieved by using random-access 3D imaging with an acousto-optic lens (3D-AOL) to steer a 2P laser beam to arbitrary preselected locations in the imaging volume.^461^ Moreover, the same technology can be used to correct for brain movement in real time, which can be problematic for imaging of small structures during behavioral tasks.^462^ Selective imaging of ROIs with 3D-AOL microscopy enables considerably higher temporal resolution than imaging the entire volume, since the inertia-free focus and scanning is rapid and the ROIs typically occupy a small fraction of the volume.^414^^,^^436^^,^^463^ In addition, this type of acquisition streamlines curation, analysis, storage, and sharing, because only the required data are collected.

**Fig. 24:**
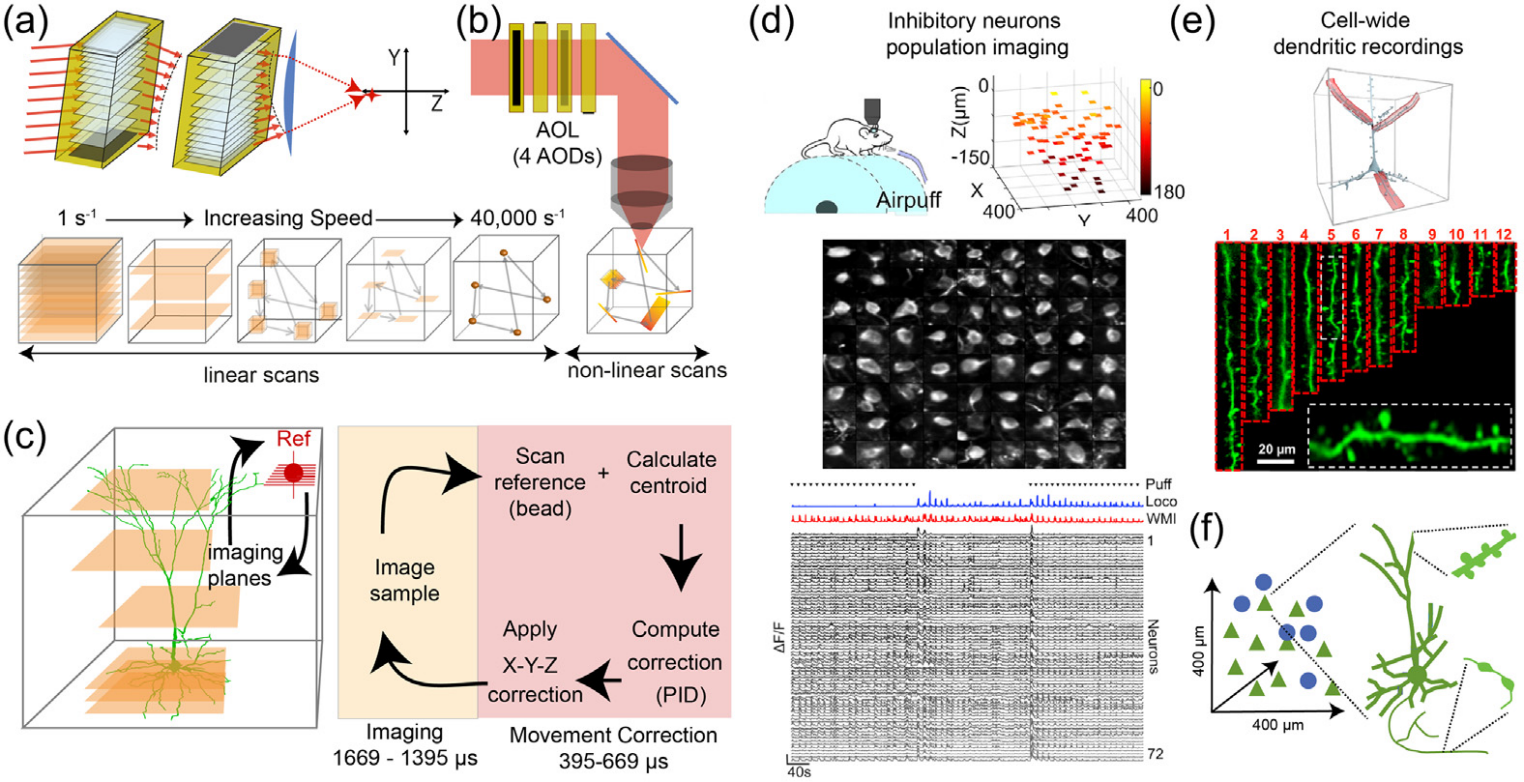
Principles and applications of 3D-AOL microscopy. (a) Schematic of pair of AO deflectors (AODs) with chirped sound waves that curve the optical wavefront of a laser beam (red lines). Modified from Ref. 463. (b) Two pairs of orthogonally aligned AODs that make up a spherical AOL that can focus and scan a laser beam in 3D. Cubes below illustrates multiple linear (left) and nonlinear (right) drive-based imaging modes. Modified from Ref. 414. (c) Left: illustration of 3D movement correction with XY and Z scans of a bead interleaved with multiplane imaging. Right: sequence of operation to track a reference object in 3D and compensate imaging for brain movement. Modified from Ref. 462. (d) Selective imaging of sparsely distributed inhibitory interneurons in the cerebellum. Top left: awake mouse on a treadmill. Top right: locations of somatic ROIs in 3D-AOL imaging volume of mouse expressing GCaMP6f in Golgi cells. Middle: montage of 72 selectively imaged Golgi cell somata. Bottom: activity traces extracted from the cells during locomotion, whisking (WMI) and mild air puff to the whiskers (Puff). Modified from Ref. 465. (e) Image of pyramidal cell with dendritic branches selectively imaged with multiple line scans (Ribbon scanning). Aligned images of individual dendritic segments with spines clearly visible (middle). Modified from Ref. 466. (f) Schematic illustrating multiscale imaging functionality of 3D-AOL microscopy, which enables simultaneous imaging of neuronal populations, dendrites, spines and axons present in a local circuit.

3D-AOL laser scanners consist of two orthogonally arranged pairs of AO deflectors, with counter-propagating acoustic waves that fill the ${\mathrm{TeO}}_{2}$ crystals, creating dynamic diffraction patterns that can steer and focus a 2P laser beam in 10–25  μs^436^^,^^455^^,^^464^ [Figs. 24(a), 24(b)]. Linear acoustic drives enable random access focusing of a laser beam to any point in the 3D imaging volume (called 3D-RAMP)^436^ or random access focusing and continuous line scanning in different x–y planes,^455^ while nonlinear drives enable continuous line scans in any arbitrary direction (x, y, z) at tens of kilohertz.^467^ Unlike resonant galvanometers, 3D-AOL line scans can be varied in orientation, length, and dwell time ($∼10^{7}\text{–}10^{5}\;s/\text{pixel}$). A compact design version of the 3D-AOL has a 10 × 10 × 25 cm footprint and can be introduced into the optical path of conventional 2P microscopes providing an imaging volume of $400\times 400\times 400\;{\mu m}^{3}$.^414^ Real-time correction for brain movement is achieved by tracking a fluorescent object in the imaging volume [typically a bead; Fig. 24(c)] with closed-loop FPGA-based image processing and compensating for the movement with a rigid translation of the imaging volume using the 3D-AOL scanner with update rates up to 1 kHz.^462^

3D-AOL microscopy has been used with GECIs to image neuronal population activity in the forebrain of tethered zebrafish while performing swim bouts,^462^ and populations of excitatory^463^^,^^468^^,^^469^ and inhibitory neurons^465^^,^^470^ in mice [Fig. 24(d)]. Its high spatial resolution has enabled imaging of large populations of axons in the cerebellum^471^ and selective imaging of dendritic trees and spines with millisecond precision with either 3D-RAMP^436^^,^^463^ or multiple line scans^466^ [Fig. 24(e)]. It is particularly suitable for high-speed multiscale imaging of neurons and neuronal structures that are sparsely distributed within local circuits [Fig. 24(f)].

Current developments include increasing the imaging volume and speeding up movement-stabilized imaging of entire dendritic trees. In the medium-term, this technology could be optimized for movement-stabilized voltage imaging^100^ and photo-stimulation applications. On the longer-term, ultra-high-speed adaptive optics capability of this technology^467^^,^^472^ could be useful for a range of applications, including higher spatiotemporal imaging of neuronal circuits, optical tweezers, and nanofabrication.

### 5.4 Dense Volumetric Imaging with Light Beads Microscopy

**Fig. 25:**
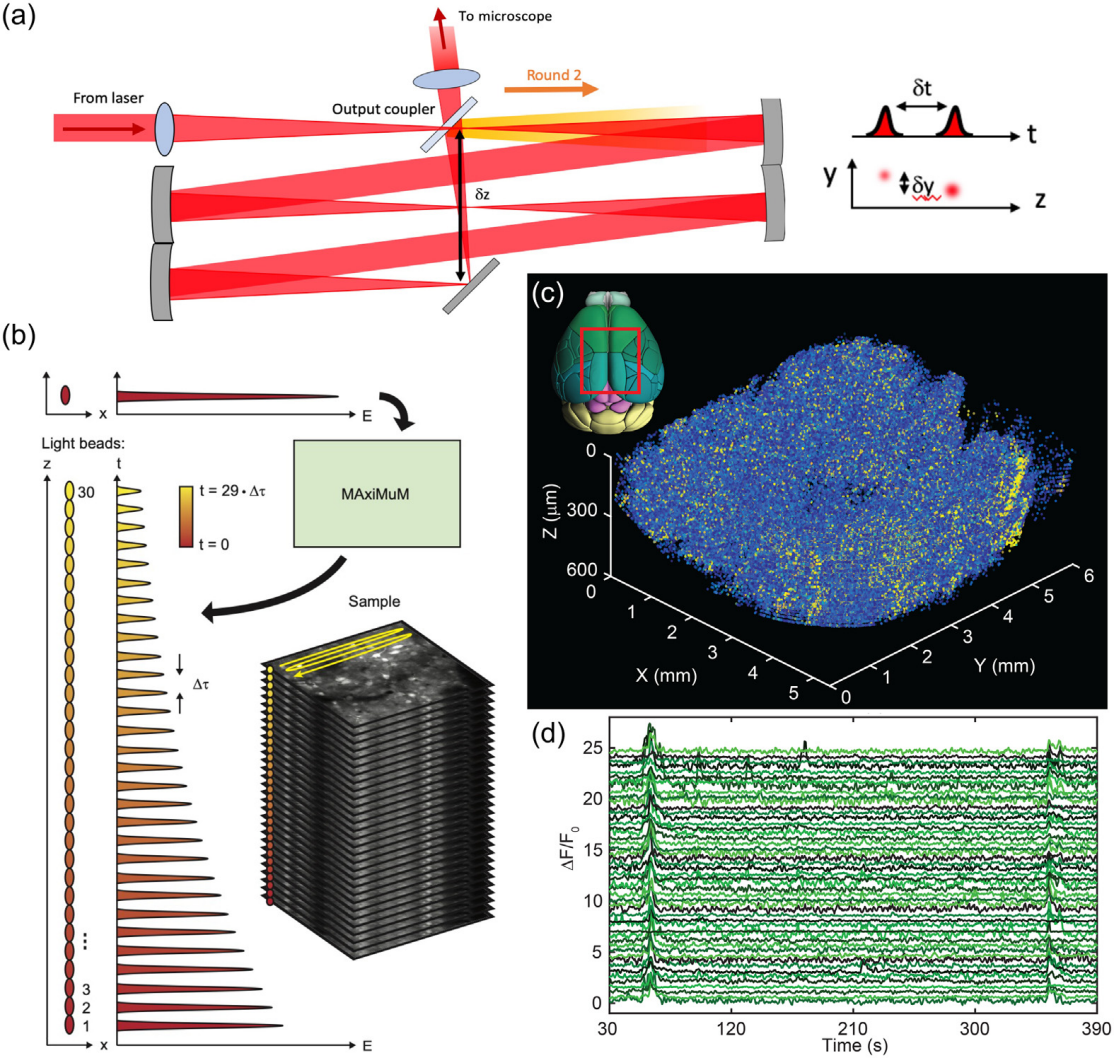
Light Beads Microscopy: elements and application. (a), (b) The Many-fold Axial Multiplexing Module (MAxiMuM) enables 30-fold temporal multiplexing in the axial direction using a cavity-based design resulting in a set of 30 axially focused beams, “Light Beads” at the sample with $\sigma_{x,y}∼1.1\;\mu m$ and $\sigma_{z}∼13\;\mu m$. A high energy pulse undergoes 30 roundtrips in a slightly imbalanced 8f reimaging cavity and during each round trip a sub-pulse at an appropriate delay is coupled out. Choosing the reflectivity of the output coupler, the exponential falloff of pulse energy can be exactly matched to the required power increase in scattering tissue for all beads. (c) Volumetric recording of 807,748 neurons within $∼5.4\times 6\times 0.5\;{\mathrm{mm}}^{3}$ at 2.2 Hz within the mouse cortex. 3D rendering of neuron spatial coordinates for a 9-minute recording. (d) Subset of 50 traces from the total of 807,748 extracted neurons, offset: 0.5 $\Delta F/F_{0}$.

Another approach to address a trade-off between resolution, speed of acquisition and the volume size of the recorded neuronal population is Light Beads Microscopy (LBM)^415^ that pushes multiphoton imaging to the limits dictated only by the tolerable biological radiation exposure and the nature of fluorescence. In LBM, a set of axially separated and temporally distinct foci record the entire axial imaging range near-simultaneously, enabling volumetric recording at $1.41\times 10^{8}\;\text{voxels}$ per second only limited by fluorescence lifetime (Fig. 25). Combined with an acquisition strategy that allows recoding of each distinct spatial voxel by only one laser pulse, this results in maximization of generated fluorescence light per unit excitation power.

LBM has been applied to achieve cellular resolution recordings within a volume of ∼5.4×6×0.5  mm spanning both hemispheres of the mouse cortex and containing more than 1 million neurons at ∼2  Hz as well as other configuration in which volume size is traded for recording speed. These data provided evidence of covariance of neuronal activity within the population of stimulus-tuned neurons separated by several millimeters across the brain at single-trial level representing information on internal states or uncontrolled aspects of external stimuli and behavior.^415^

We expect that in the future, SNR of LBM can be further enhanced using shorter laser pulses and higher bandwidth amplification between the photodetectors and digitizers. Together with development of more efficient, red-shifted indicators these developments are expected to extend the depth of imaging and the size of recorded neuronal population within the limits of tolerable sample exposure to laser light. The ability to record from such large neuronal populations with cellular resolution opens up a range of opportunities for understanding the neurocomputation principles underlying multiregional encoding and processing of sensory and behavioral information across the mammalian brain.

### 5.5 Non-Degenerate 2P Microscopy

**Fig. 26:**
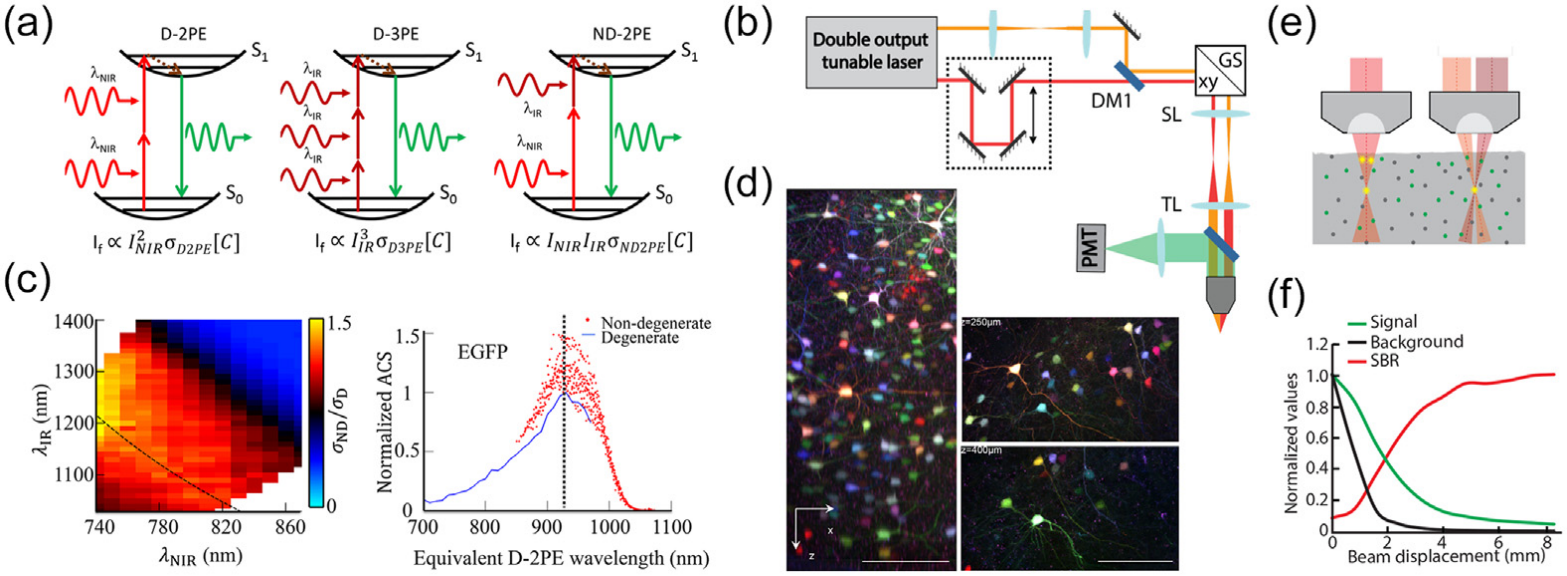
ND-2PE with collinear and displaced beams. (a) Schematic Jablonski energy diagram for D-2PE, D-3PE, and ND-2PE. $I_{f}$, fluorescence signal intensity; $I_{\mathrm{NIR}}$ and $I_{\mathrm{IR}}$, intensities of the two laser beams; $\lambda_{\mathrm{NIR}}$ and $\lambda_{\mathrm{IR}}$, the wavelength of the two laser beams; $\sigma_{D2\mathrm{PE}}$, $\sigma_{D3\mathrm{PE}}$, and $\sigma_{\mathrm{ND}2\mathrm{PE}}$, cross-sections for D-2PE, D-3PE, and ND-2PE; [*C*], fluorophore concentration. (b) Simplified schematic of ND-2PE microscope setup: GS-XY, galvanometer scanner; SL, scan lens; TL, tube lens; DM, dichroic mirror; OL, objective lens; PMT, photomultiplier tube. (c) Left: color-coded normalized non-degenerate absorption cross-section for EGFP as a function of the combination of NIR and IR wavelengths. The cross-section value at each combination of wavelengths was normalized by the peak value of this fluorophore. The isocline corresponding to the ground to excited state transition energy is indicated in dashed black line. Right: normalized cross-section values as a function of the equivalent degenerate wavelength $2/\lambda_{D}=1/\lambda_{\mathrm{NIR}}+1/\lambda_{\mathrm{IR}}$ are shown in red. Independently measured degenerate cross-section values normalized by its peak within the equivalent range of the total photon energy are shown in black. The black dashed line indicates the position of the peak degenerate absorption used for the normalization procedure (930 nm). These wavelengths correspond to the energy isocline shown in panel (b). (d) Simultaneous excitation of four different fluorescent proteins in Brainbow mouse cortical tissue using ND-2PE with $\lambda_{\mathrm{NIR}}=850\;\mathrm{nm}$, and $\lambda_{\mathrm{IR}}=1100\;\mathrm{nm}$. Panel (d) is reproduced with permission from Ref. 473. (e) Left: D-2PE excites the sample both near the surface and within the focal volume. Right: by selecting the two excitation beams for ND-2PE outside of the excitation spectrum of the fluorophores and spatially displacing the beams, the sample is excited only in the focal volume where two beams overlap. (f) Simulation results for the normalized values of signal, background, and SBR versus the beam displacement from the colinear configuration.

In the 2P imaging modalities described above (Secs. 5.1–5.4), a fluorophore was excited by the simultaneous absorption of two photons of the same energy within the near infrared spectrum, derived from the same pulsed laser beam. This is known as “degenerate” 2P (D-2P) excitation. Alternatively, the same energy needed for the transition to the excited state can be delivered via absorption of two photons of different energy (i.e., different color) [Fig. 26(a)]. This is “non-degenerate” 2P excitation (ND-2PE). ND-2PE has a long history in the chemical physics and microscopy.^474^^–^^477^ Using ND-2PE, we can vary the energies of the individual photons ($\hbar \omega_{1}$ and $\hbar \omega_{2}$) while keeping the sum energy $E=\hbar (\omega_{1}+\omega_{2})$ constant. The first laser beam can be tuned within the standard near-infrared range of excitation wavelengths used in conventional 2P microscopy, placing the second laser beam within the short wavelength infrared wavelengths range for excitation of visible emission fluorophores (green to red). Due to the requirements of two temporally synchronized pulsed beams [Fig. 26(b)], it is preferential to use a single source as a master oscillator. Commercially available turn-key options include dual output femtosecond lasers with one beam fixed at 1040-nm and a second tunable beam.^478^ To gain more tuning flexibility, one can also use a femtosecond Ti:Sapphire laser pumping an Optical Parametric Oscillator (OPO).^479^^–^^481^

ND-2PE results in an increase in the probability of 2P excitation,^480^^,^^482^ quantified as the excitation cross-section – an important parameter that, for a given fluorophore concentration and laser power, translates into brighter fluorescence. Because laser illumination may have adverse effects on live biological tissues, large cross-section is always a desirable feature at the focus of fluorophore engineering.^277^ Importantly, ND-2PE does not alter the shape of 2P absorption spectrum, so that that the peak absorption remains the same [Fig. 26(c)].

ND-2PE has been used to extend the excitation wavelength range providing a third “virtual” excitation wavelength.^473^^,^^483^^,^^484^ Using synchronized pulses from a femtosecond laser and an OPO, Mahou et al. demonstrated simultaneous excitation of four different fluorescent proteins in Brainbow mouse cortical tissue^473^ [Fig. 26(d)]. ND-2PE has also been explored to increase spatial resolution (Refs. 485, 486 2004 #2024) and penetration depth in scattering media.^487^^,^^488^ Importantly, ND2P excitation can be used to suppress the background (out-of-focus fluorescence) by choosing their wavelength outside of the D-2PE spectrum of the fluorophores and shaping the beams to overlap only at the focal spot.^478^^,^^488^^–^^491^ For example, one can arrange the beams to enter the back aperture of the objective side-by-side parallel to each other and the optical axis [Fig. 26(e)]. This arrangement reduces their overlap above the focal plane minimizing the background at the price of reducing the effective numerical aperture (NA) for each beam.^478^^,^^492^ Simulation studies using a beam propagation model have shown that the amount of enhancement in the signal-to-background ratio (SBR) depends on the amount of displacement [Fig. 26(f)]. Both the efficiency of ND-2PE (signal) and out-of-focus fluorescence (background) decrease by increasing the spatial separation of the beams. However, the background decreases at a higher rate leading to the overall increase in SBR.^478^

Further studies are required to optimize the key parameters important for achieving the optimal SBR in ND-2PE: the amount of displacement, the beam size, and the quality of the beam overlap in the focal volume. These improvements would allow increasing the depth penetration of 2P microscopy, particularly in cases of dense fluorescence labeling.

### 5.6 Three-Photon (3P) Microscopy

**Fig. 27:**
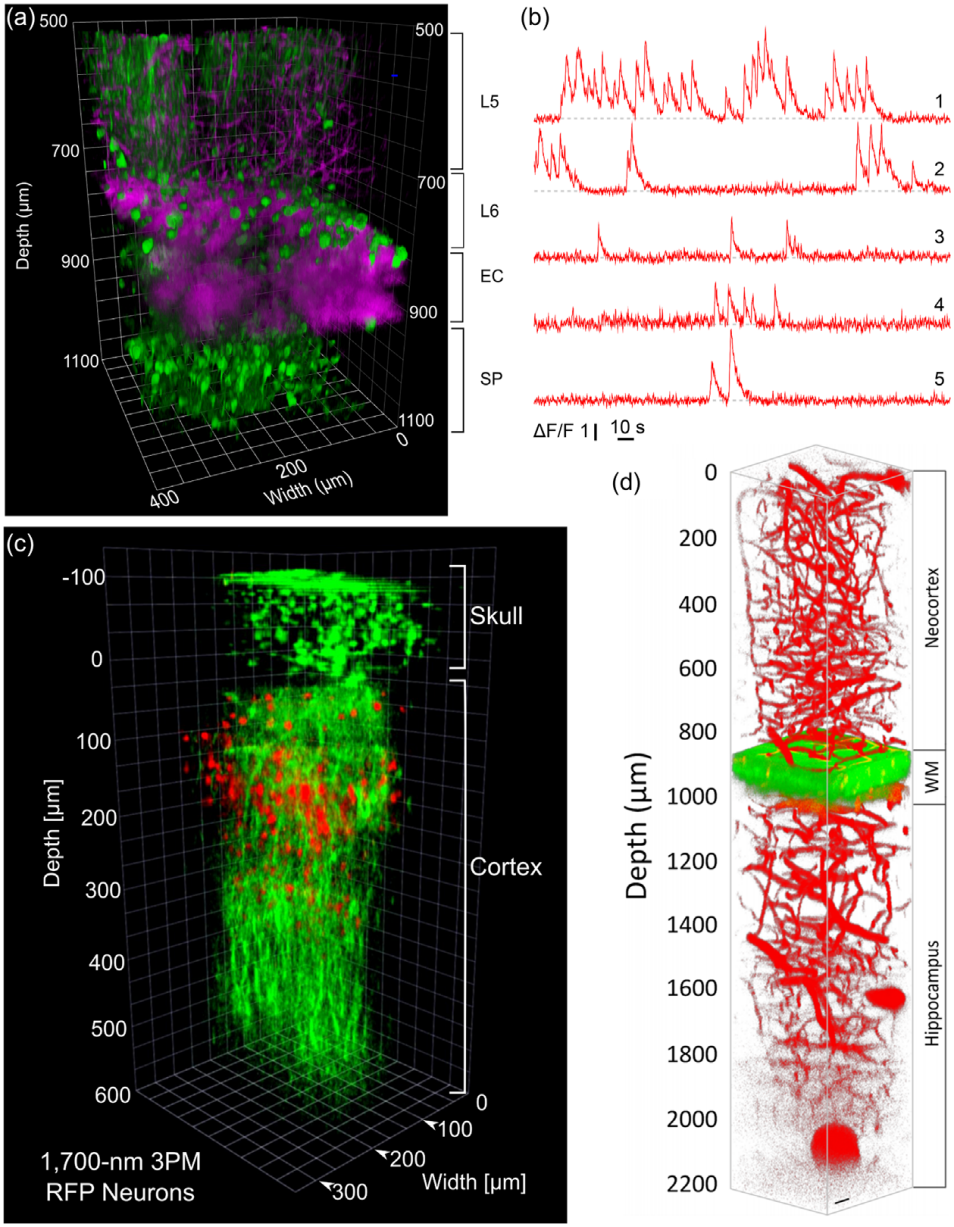
Deep imaging with 3PM. (a) 3PM of GCaMP6s-labeled neurons in the mouse cortex and the hippocampus. Green, fluorescence; magenta, third-harmonic generation (THG). (b) Activity recording in the Stratum Pyramidale layer of the hippocampus at ∼1  mm depth. Only a small portion of the 48-minute recording session is shown. Reproduced with permission from Ref. 416. Springer Nature. (c) Imaging through the intact mouse skull. Red, RFP-labeled neurons in a Brainbow mouse, male, 12 weeks. Green, THG. The zero depth is set just beneath the skull. Reproduced with permission from Ref. 495. Springer Nature. (d) *In vivo* 3PM of mouse brain vasculature labeled with quantum dots (Qtracker 655) to ∼2.2  mm depth. Reproduced with permission from Ref. 496, © 2019 American Chemical Society.

As we discussed above, 2P microscopy (2PM)^428^ in combination with genetically expressed fluorescent probes^493^ has been enormously influential in neuroscience. It opened the door for studies of brain structure and function with cellular and subcellular resolution in living animals. The main limitation of this technology is depth penetration: SBR deteriorates when imaging deep into the brains.^494^ This problem is commonly known as out-of-focus background fluorescence (mentioned in Sec. 5.5). 3P microscopy (3PM, Fig. 27)^497^ overcomes this “fundamental” limitation.

The deep tissue penetration capability of 3PM derives from the longer excitation wavelengths to reduce tissue scattering^497^^–^^499^ and the higher-order nonlinear excitation to steepen the drop in the probability to excite fluorescence away from the focus.^415^^,^^497^^,^^500^^,^^501^ In contrast to 2PM, 3PM is not limited by out-of-focus fluorescence, so far even in densely labeled samples.

Long wavelength 3PM is being applied to deep tissue imaging beyond the depth limit of 2PM, such as 3PM of GCaMP-labeled neurons in the mouse hippocampus^497^ and 3PM of quantum-dot-labeled mouse brain vasculature at >2.1  mm depth.^496^ 3PM is capable of imaging through a highly turbid layer such as through the intact skull of adult mouse^495^ and adult zebrafish.^502^ 2PM and 3PM can be combined for simultaneous imaging of both the cortical layers and the subcortical regions.^424^ By leveraging blue-shifted 3P excitation, simultaneous imaging of multiple fluorophores can be achieved. For example, commonly used blue, green, yellow and red fluorescent proteins can be 3P excited using a single excitation beam at 1340 nm,^503^ enabling four color, deep tissue imaging.

While long wavelength 3PM has significantly increased the penetration depth of high spatial resolution fluorescence imaging, there are a number of limitations that must be overcome for 3PM to achieve its full potential. 3P excitation is inherently weaker than 2P excitation.^504^^,^^505^ In fact, the 3P signal strength sets the practical limit of the imaging depth of 3PM today.^506^ Therefore, improving the 3P signal is essential to increasing the 3P imaging depth. Large 3P cross-sections,^503^ adaptive optics,^507^^,^^508^ and imaging the regions of interest only^509^ can improve the 3P signal. Furthermore, the laser source for 3PM is not yet optimized for deep tissue penetration, and the complexity and cost of the excitation source is a major barrier for the applications of 3PM. An excitation source with cost and user experience similar to a Ti:Sapphire laser for 2PM is necessary to make deep tissue 3PM a routine instrument for brain research. Finally, foundational knowledge of many parameters for 3PM is not yet available. For example, the spectral properties of 3P cross-sections have not been studied for nearly all dyes and fluorescent proteins at wavelengths >1100  nm. Although the scattering and absorption characteristics of the mouse brain between 1300 nm and 1700 nm have been measured recently,^495^^,^^510^ systematic studies of tissues are needed for other brains (e.g., fly, fish, non-human primate, etc.) to define the application space for 3PM and lower the barrier of adoption of the 3PM technique beyond the mouse brains.

### 5.7 Wearable Multiphoton Microscopes

**Fig. 28:**
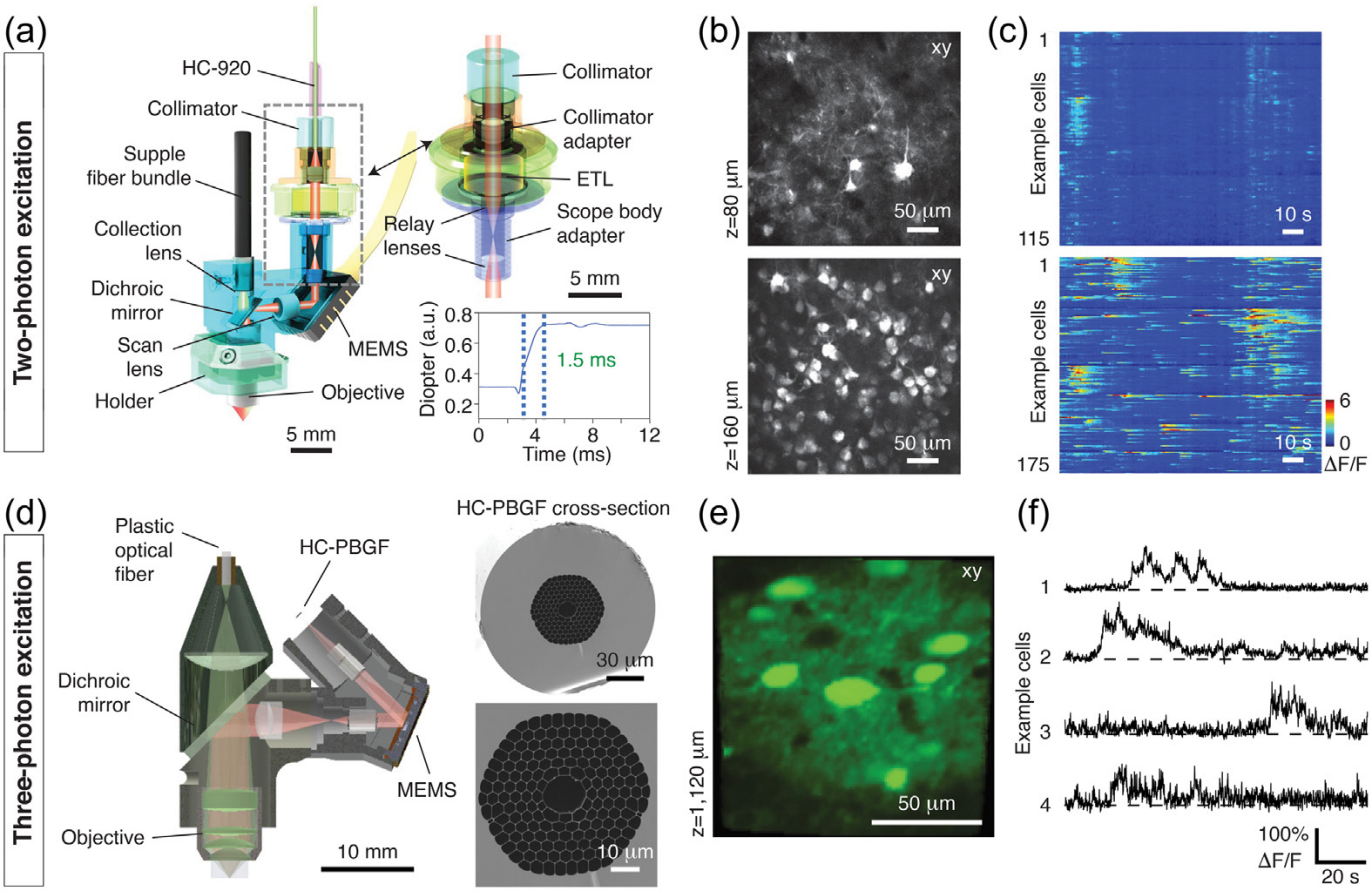
2P and 3P wearable microscopes for cellular-resolution imaging in freely behaving animals. (a) Example 2P miniscope for high-resolution imaging at depth. This implementation weighs 4.2 grams and includes an electrically tunable lens (ETL) (top right) for rapid focus adjustment across a 180  μm focal range (bottom right). Using a microelectromechanical system (MEMS) scanner, the miniscope allows 10 Hz recordings at 512 x 512 pixels resolution across a 420  μm FOV. (b) Example images of GCaMP6s-expressing neurons acquired at the indicated focal depths (z) in the mouse prefrontal cortex. (c) ${\mathrm{Ca}}^{2+}$ transients from dendritic (top) and cell body regions of interest (ROIs) (bottom) within the focal planes shown in (b). (d) 3P miniscope for deep, high-resolution imaging. This microscope weighs 5 grams and includes a 1.2-m hollow-core photonic bandgap crystal fiber (HC-PBGF) (right) for low-dispersion delivery of the ∼1,320  nm excitation light-pulses. A plastic optical fiber collects the emitted fluorescence for remote detection by photomultiplier tubes (PMTs). (e) Image of GCaMP6s-labeled rat cortical neurons at the indicated focal depth (z) in the posterior parietal cortex. (f) ${\mathrm{Ca}}^{2+}$ spiking from four example neurons recorded at 1,120  μm cortical depth. (a)–(f) Adapted with permission from Nature Publishing Group: (a)–(c) from Ref. 418 and (d)–(f) from Ref. 417.

Both 2P and 3P microscopes have been miniaturized to enable imaging in freely behaving animals. Compared to 1P miniscopes (Sec. 4.2), their 2P counterparts offer higher resolution, both laterally and axially, and larger depth penetration (up to ∼300  μm) [Figs. 28(a)–28(c)].^372^^,^^373^^,^^418^ Depending on the scanning modality, frame rates of up to ∼40  Hz (but typically ≤15  Hz) can be achieved. 3P miniscopes offer even larger imaging depths (up to ∼1.1  mm in the cortex) but require low-repetition-rate amplified laser systems and special fibers in less widespread use [Figs. 28(d)–28(f)].^417^ Due to their reliance on short-pulse lasers and photomultiplier tubes (PMTs) for fluorescence excitation and detection, respectively, multiphoton miniscopes are fiber-coupled. The use of hollow-core (HC) photonic crystal fibers has allowed efficient fluorescence excitation at defined wavelengths [Figs. 28(a), 28(d)]. However, multiphoton miniscopes’ attainable field of view tends to be smaller (up to 420  μm but typically ≤300  μm) than their 1P counterparts [Figs. 19(b), 28(b), 28(e)].^372^^,^^373^ Additionally, their relatively low frame rate and depth-of-field can complicate stable recordings during animal behavior-induced or tissue-intrinsic movements.

In the future, improved laser scanning and optical components may enhance multiphoton miniscopes’ FOV, frame rate, and axial beam control [Fig. 28(a)]. Other features may include the ability to perform all-optical or combined imaging and electrophysiological interrogations in unrestrained animals.^352^^,^^372^

### 5.8 Adaptive Optics in Multiphoton Microscopy

**Fig. 29:**
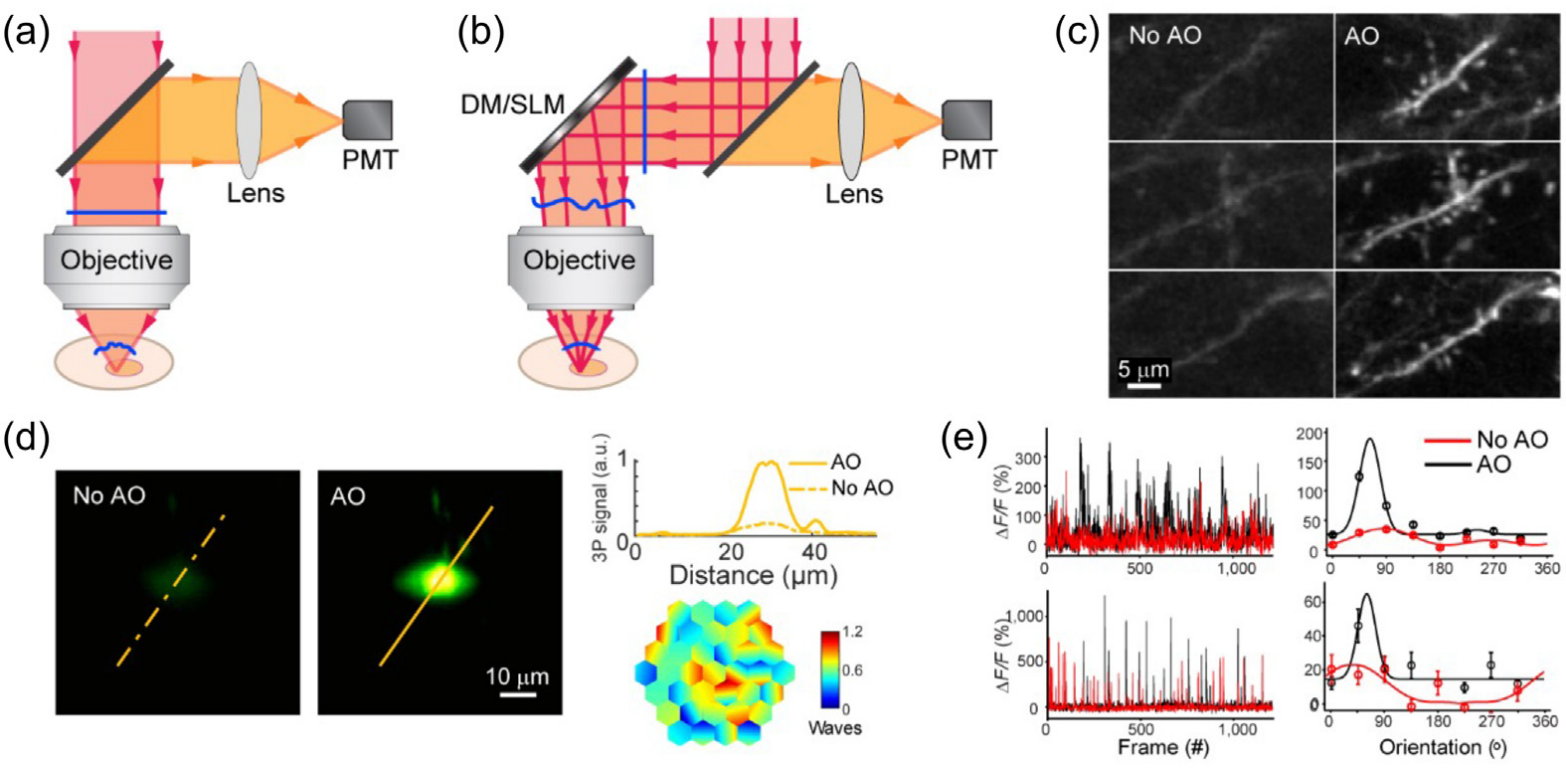
Adaptive optics enables high-resolution multiphoton microscopy at depth. (a) Wavefront distortion of the excitation light can be (b) corrected with a deformable mirror (DM) or liquid crystal spatial light modulator (SLM) to form a diffraction-limited excitation focus. Blue: wavefront. Red: excitation light. Orange: fluorescence. (c) 2P fluorescence images of dendrites and dendritic spines (606−608  μm depth) in the mouse cortex. (d) 3P fluorescence images of a neuron (414  μm depth) in the mouse spinal cord. (e) AO increases ${\mathrm{Ca}}^{2+}$ transient strength ΔF/F and enables detection of orientation selectivity.

Adaptive optics (AO) is a method used in brain imaging to reduce the blurring effect of wavefront distortions and recover diffraction-limited performance deep within the brain tissue. AO was first developed for ground-based telescopes to measure and correct the distortion introduced by the turbulent atmosphere on wavefronts of distant astronomical objects.^511^^,^^512^ It was later adopted by ophthalmologists to correct the aberrations of the eye for high-resolution imaging of retinal structures.^513^^,^^514^ With the increasing popularity of multiphoton fluorescence microscopy for *in vivo* brain imaging, AO methods have been developed to measure and correct the distortion that brain tissue imparts on the wavefront of the excitation light [Fig. 29(a)] for high-resolution imaging at depth.^515^

An AO system corrects the distortion or optical aberrations of the image-forming light (i.e., the excitation laser in multiphoton microscopy) by controlling its wavefront with a phase device such as a deformable mirror or liquid-crystal spatial light modulator, so that a diffraction-limited focus can be formed within the sample [Fig. 29(b)]. Effective correction requires accurate measurement of sample-induced aberrations. Methods similar to those applied in astronomy and ophthalmology, where the distortion is directly measured by a wavefront sensor (typically made of a lenslet array and a camera), have been demonstrated for sample preparations with minimal scattering.^516^^,^^517^ For samples with substantial scattering, indirect wavefront sensing methods have been developed. Some measure wavefront distortion by manipulating the excitation wavefront and detecting its effect on fluorescent signal/image,^518^^–^^520^ while others measure the electric PSF of the excitation focus directly.^521^

Both direct^522^^,^^523^ and indirect AO methods^521^^,^^524^^–^^526^ have been applied to 2P fluorescence microscopy for high-resolution structural and functional imaging of the brain down to layer 5b of the mouse cortex^523^ and through thinned skull [Fig. 29(c)].^527^ Combined with 3P fluorescence microscopy, AO has enabled non-invasive high-resolution imaging of structures such as subcortical synapses and spinal cord neurons [Fig. 29(d)].^506^^,^^508^ The recovery of diffraction-limited resolution enabled accurate measurement of ${\mathrm{Ca}}^{2+}$ activity from thalamic boutons in layer 4 of the mouse primary cortex [Fig. 29(e)], which led to the discovery of extensive orientation-selective thalamic inputs.^528^

The proof-of-principle experiments described above have firmly established the importance and effectiveness of AO for high-resolution brain imaging at depth. Now it is time for the field to go beyond purely technological demonstrations so that AO can contribute to biological discoveries in the brain. The biggest obstacle is how to integrate AO modules into existing and new microscopes, and to ensure its ease of use and robust performance even in the hands of users with no optical expertise. Further engineering and collaboration with microscope companies will be necessary to realize this goal.

## 6 Two-Photon Optogenetics

The advent of optogenetic actuators (Sec. 3.10), has ushered a new era in causal neuronal circuit interrogation. Yet, in most “traditional” applications of optogenetic perturbation an entire population of neurons is excited or suppressed together, and furthermore the experimenter often does not observe the neuronal response to the perturbation. However, in essentially every neuronal circuit, the activity of different neurons within a population is heterogeneous. Further, neuronal excitability varies in both space and time, strongly affecting responses to optical stimulation. Thus, providing more nuanced insights into key problems in systems neuroscience like the connection between neuronal code and behavior, requires the advancement of neurophotonic methods with increased precision and built-in optical readout. The resulting set of techniques are often termed “circuit optogenetics”^529^ or “all-optical” circuit interrogation.^311^^,^^530^^,^^531^

### 6.1 2P Optogenetic Photostimulation

**Fig. 30:**
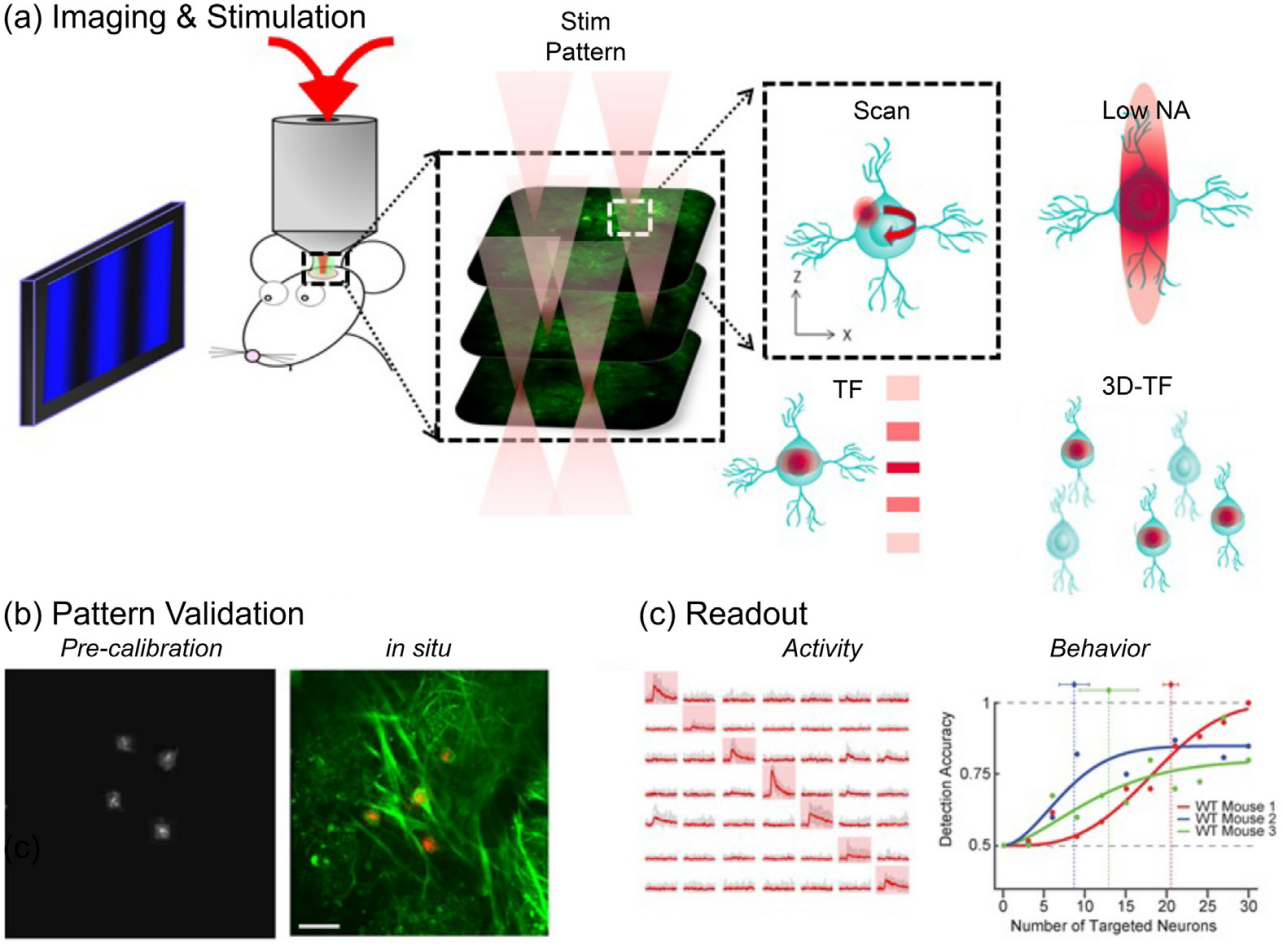
Precision optogenetic modulation of neuronal circuits and behavior. (a) All-optical precision circuit interrogation is enabled by combining 2P imaging and cell-targeted holographic stimulation of a distributed pattern of multiple neurons, typically using a dedicated low-repetition rate (amplified) femtosecond laser. The illumination is extended across each neuron’s membrane by either rapid scanning, reducing the effective numerical aperture (at the cost of optical sectioning) or by parallel illumination of temporally focused extended shapes in two or three dimensions. (b) An array of methods are being developed to calibrate the projected light patterns, adjust as needed, and “smooth” their intensity profile (by minimizing holographic speckle^547^), and in some cases, validate and correct their precise targeting *in situ* inside the tissue.^548^ (c) Beyond target validation, all-optical precision neurophotonic interfaces enable direct readout of the effects of circuit perturbations with simultaneous imaging of neuronal activity (left panel from Ref. 536). This paradigm can be used to infer circuit connectivity and dynamics. One can also infer neuronal activity indirectly through animal behavioral reports of its (perturbed) perceptual experience (right panel, from Ref. 538), which informs us about the behavioral relevance of specific neurons and coding features.

From a technical standpoint, stimulating distributed sets of pre-defined neurons in the brain with single-cell resolution has become possible through the advent of holographic 2P optogenetic photostimulation (e.g., Refs. 532–536), which has transformed the study of the behavioral relevance of specific circuits and activity patterns.^313^^–^^315^^,^^537^^,^^538^ 2P optogenetic photostimulation utilizes the principle of non-linear 2P absorption to excite light-sensitive opsins within a confined axial plane deep into tissue.^308^^,^^532^^,^^539^ Holographic optical neuronal interfaces (HONIs, in analogy to their holographic optical trap (HOT) predecessors) use phase-modulating spatial light modulators (SLMs) to diffractively shape a distributed excitation pattern in two or three dimensions.^449^^,^^540^^–^^543^ Tightly focused diffraction-limited focal spots maximize 2P excitation efficiency within the focal volume but are not very efficient in modulating behavior of the targeted neuron, because only a small patch of the cell membrane is affected. Therefore, practical implementation of 2P optogenetics commonly relies on one of the two general strategies: rapidly scanning a diffraction-limited spot to cover a large fraction of the cell’s membrane^310^^,^^314^^,^^315^^,^^532^^,^^536^ or defocusing (i.e., spatially expanding) the focal spot to cover the entire cell body^533^^,^^535^^,^^537^^,^^539^^,^^544^^–^^546^ [Figs. 30(a)–30(b)]. The first approach generally trades robustness of optogenetic actuation with temporal precision, and typically lacks the high temporal resolution necessary for generation of precise spike timing to reproduce spatiotemporally patterned activity. The second approach requires more complex hardware. In addition, temporally focused 2P excitation^311^^,^^438^^,^^544^ is used to decouple the lateral and axial dimensions of the excitation spots by shaping the light pulse’s spatiotemporal profile.

All-optical HONIs technology is a highly active area of neurophotonics and multiple recent studies have advanced key performance attributes of the optogenetic probes and the spatiotemporal characteristics of the generated light patterns. Several new families of red-shifted optogenetic probes were developed and/or characterized primarily for this application including Chrimson,^337^ ReachR,^549^ ChRome^535^ and ChRmine,^314^ soma-targeting strategies were developed for increased efficacy and precision,^533^^,^^535^^,^^550^^,^^551^ and transgenic animals that express these probes under genetic control have been developed^546^^,^^552^ (see also Sec. 3.10). While these opsins have the desirable property of being effectively excited at the ∼1040  nm wavelengths of many common pulsed lasers, their extended excitation shoulder at shorter wavelengths means that they suffer from unwanted “cross-talk” excitation from the 2P imaging laser (which becomes worse for more sensitive probes). One possible alternative strategy being developed is to combine a blue-shifted opsin like CoChR with a red-shifted indicator like jRCamp.^534^^,^^551^ While earlier studies in this area excited neurons with moderate temporal precision (typically >50  ms), sensitive probes and effective excitation from amplified laser sources with low repetition rate now enable millisecond-precise stimulation,^314^^,^^533^^,^^535^^,^^538^^,^^546^ turning SLM switching times into the limiting factor. Increasing this performance parameter is possible by employing rate-optimized SLMs^314^ (where the liquid crystal elements are also heated), by sequentially stacking several SLMs,^314^ or by sequentially illuminating different sectors of the SLM^553^^,^^554^ —a technique first developed for rapid pulse shaping.^555^ While holographic patterning of diffraction-limited focal spots in 3D is quite straightforward, development of temporally focused holographic stimulation required creative optical solutions.^311^^,^^531^^,^^542^^,^^556^^–^^558^

### 6.2 Combining Precision Optogenetics with Psychophysical Measurements

These advances, together with a complementary toolbox of experimental and analysis techniques, have set the stage for neurophotonic probing of the precise relationship between subtle circuit manipulations and the observed behavior [Fig. 30(c)]. Population-wide circuit perturbation often leads to dramatic and easily detectable behavioral effects. In contrast, early attempts to apply precision optogenetics in awake behaving animals have failed to evoke measurable behavioral effects.^532^ To date, several (non-exclusive) strategies to address this challenge have emerged. The first one is to intensify the manipulation, e.g., by increasing its duration.^313^^–^^315^ The second strategy is to train the animal to detect or discriminate among purely synthetic percepts. In the process of such training, animals become increasingly sensitive to subtle perturbations, reducing their perceptual thresholds by several orders of magnitude. This approach underlies recent studies on the coding logic of olfactory,^538^^,^^559^^,^^560^ and visual^561^ perception. Efforts in this area are often inspired by classical studies that have used subtle electrical stimulation to bias sensory psychophysical curves.^562^

Due to the above-mentioned neurophotonic advances, complex challenges in determining the behavioral relevance of specific features of neuronal circuit activity can now uniquely be addressed with all-optical circuit interrogation solutions and with unprecedented detail. For example, this toolbox now allows experiments like “leave one out” and cross-excitation of two equivalent populations, providing unprecedented data for computational studies. Likewise, precise manipulation of stimulus timing and inter-neuron synchrony can also be precisely studied. Moreover, changing the parameters of optogenetic actuation by light, including frequency and duration, not only affects the stimulation efficacy, but also provides an important control for possible experimental confounds.^538^

## 7 Microscopic Imaging of Blood Flow and Oxygenation

Brain cells, including astrocytes and microglia, partner with neurons to maintain healthy brain activity and metabolism (see Secs. 3.6, 3.7). Another important player in this partnership is blood vessels.^563^ During neuronal activity, vasoactive molecules and ions released by neurons and glia control the diameter of local microvasculature producing changes in blood flow.^564^^,^^565^ In parallel, active brain cells consume $O_{2}$ causing an increase in $O_{2}$ extraction from blood vessels to cerebral tissue. Therefore, measurements of blood flow and oxygenation indirectly reflect neuronal activity.^566^ In addition to serving as a surrogate for neuronal activity, these measurements are important in their own right, because perfusion of tissue by blood and the partial pressure of $O_{2}$ (${\mathrm{pO}}_{2}$)—in blood vessels and tissue (the interstitial space)—arefundamental physiological parameters in health and disease.

Cerebral blood flow (CBF) can be measured volumetrically with microscopic resolution using OCT that utilizes red blood cell (RBC) motion-induced Doppler shifts (Sec. 7.1). Other optical CBF measurements include laser speckle contrast imaging (LSCI)^567^^–^^569^ and 2P imaging of RBC velocity.^407^ LSCI provides a rapid widefield qualitative characterization of the motion of light scattering particles.^570^ 2P measurements of RBC velocity require fluorescent labeling of the blood plasma or RBC.^407^^,^^571^

Approaches to microscopic measurements of $O_{2}$ in the brain utilize different principles and differ in their degree of invasiveness and sensitivity.^572^^,^^573^ Depth-resolved real time measurements of intravascular and interstitial ${\mathrm{pO}}_{2}$ with micron-scale resolution have been enabled by 2P imaging of phosphorescent nanoprobes (Sec. 3.8). This technique termed ‘2P phosphorescence lifetime microscopy’ or 2PLM (Sec. 7.2) also enables estimation of cerebral metabolic rate of $O_{2}$ by quantifying ${\mathrm{pO}}_{2}$ gradients around penetrating cortical arterioles.^574^^,^^575^

Measurements sensitive to cerebral blood flow, volume and oxygenation are commonly referred to as “hemodynamic.” One of the most popular hemodynamic imaging modalities is functional Magnetic Resonance Imaging (fMRI), a macroscopic noninvasive technology commonly used in humans that leverages paramagnetic properties of deoxyhemoglobin to generate signal. Optical hemodynamic imaging technologies create contrast due to differences in the absorption spectrum of oxy- and deoxyhemoglobin.^576^ Among them is functional near-infrared imaging (fNIRS),^577^ an optical counterpart of fMRI, which we cover in the companion paper dedicated to human optical imaging technologies. In animals, optical intrinsic signal imaging (OISI) has been extensively used for mapping of cortical activity.^578^

Photoacoustics (PA) offers another solution to hemodynamic imaging (Sec. 7.3). This is a hybrid technology that relies on absorption of photon energy to produce other physical phenomena: heat (a change in temperature) and sound (a pressure wave), which is detected by ultrasonic transducers.^579^ Photoacoustic microscopy (PAM), which required optical focusing, offers quantification of oxygenation in individual RBCs. Photoacoustic tomography (PAT) provides deeper imaging at the price of lower resolution.^580^

### 7.1 Imaging Brain Function with Optical Coherence Tomography

Cerebral blood flow (CBF) supports brain function by delivering oxygen and other nutrients, while removing waste. Neuronal activity modulates CBF through a process known as “neurovascular coupling.” Therefore, measurements of CBF and related hemodynamic parameters can reflect brain function. Earlier (Sec. 2.6), we discussed optical coherence tomography (OCT), which uses optical interferometry to detect echoes of light backscattered from different depths in tissue,^75^ in the context of imaging tissue structure. Over the years, researchers have developed a rich toolbox of functional extensions based on fluctuations of light scattered from red blood cells (RBCs), providing a measurement of brain function. These methods include Doppler,^581^ angiography,^582^ decorrelation,^583^ and capillary transients (RBC passage).^584^

**Fig. 31:**
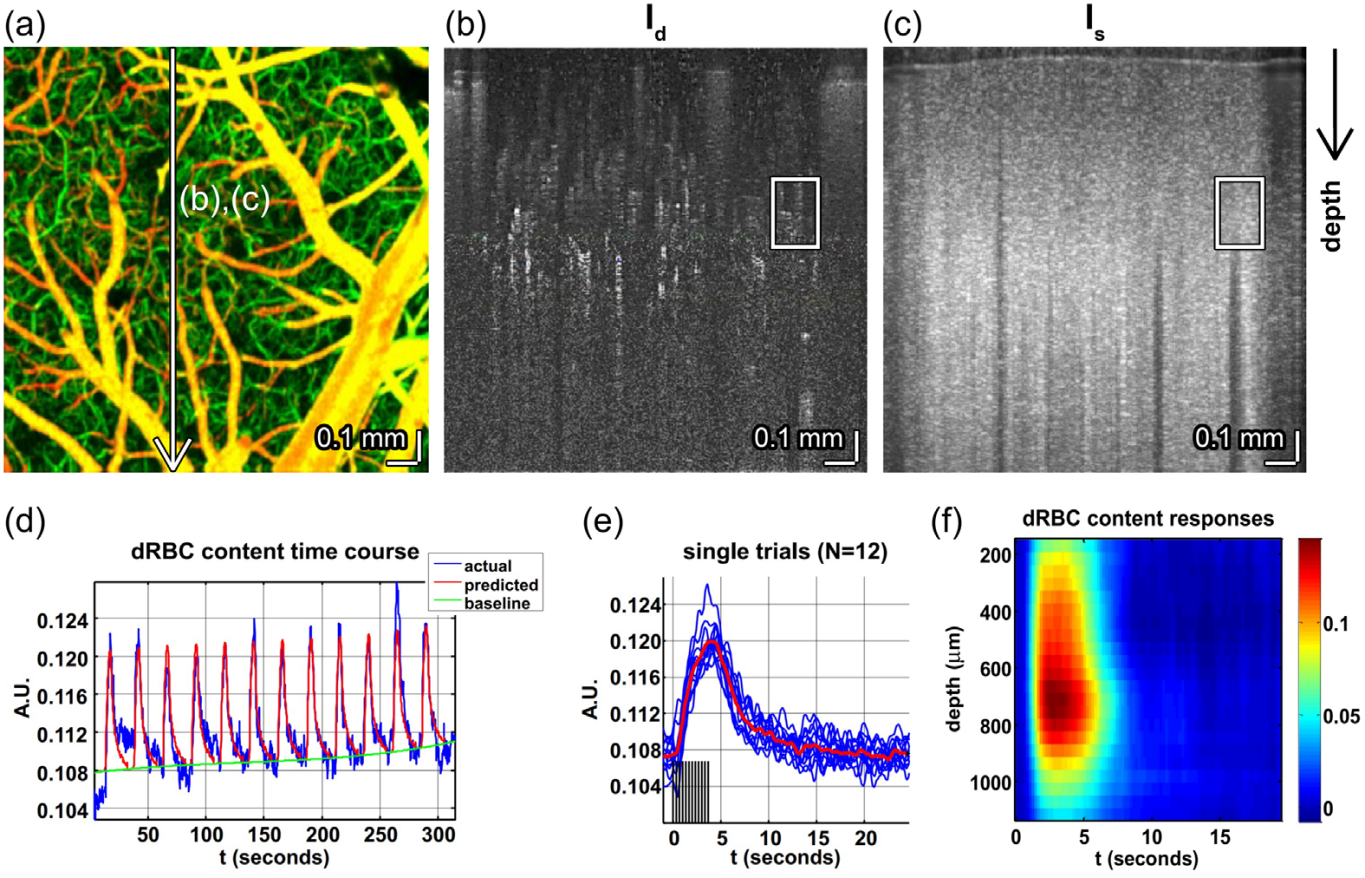
Angiography and blood flow measurements with OCT. (a) Depth-resolved OCT angiography of the rat somatosensory cortex.^587^ Location of OCT cross-sections shown on volumetric angiogram [maximal intensity projection (MIP); superficial vessels in orange-yellow, deep vessels in green]. (b), (c) Cross-sectional angiogram (dynamic intensity, (b)) and static intensity (c) images. (d), (e) Localized changes in dynamic red blood cell (dRBC) content extracted from boxes in (b) and (c), respectively. (f) Depth-resolved measurements show the strongest dRBC responses in middle cortical layers.

Compared to 2P microscopy, in general, OCT offers slightly greater penetration depths if performed at the same excitation wavelength, enables imaging through less invasive preparations, and does not require extrinsic contrast agents. Amongst optical methods, OCT has the unique advantage of acquiring with depth priority. This enables measuring simultaneously across cortical depths, revealing subtle differences between cortical laminae^585^^,^^586^ that might otherwise be obscured by physiological noise. This capability has led to OCT angiography studies that pinpoint layer IV as the cortical layer with the largest RBC response to functional activation^585^^,^^587^ (Fig. 31). Second, OCT acquires three-dimensional volumes in seconds or even fractions of a second, enabling comprehensive assessments of microvascular network dynamics. This capability has led to studies showing flow homogenization during functional activation^588^^,^^589^ as well as the retrograde propagation of dilation.^590^ Third, OCT uses time and confocal gating to reject multiply scattered light and isolate backscattered light from sub-picoliter brain volumes. Such precise control of the light path enables quantitative measurements of capillary speed,^588^ oxygenation,^591^ hemoglobin concentration,^592^ and metabolic rate of oxygen consumption.^592^ These baseline measurements are “absolute” in the sense that they can be compared between locations or subjects, and followed over time, unlike conventional laser speckle and optical intrinsic signal imaging. Such baseline measurements provide critical context to interpret neuronal activity-associated changes in hemodynamics or metabolism.

Emerging trends include 1700 nm OCT for imaging of deep cortical and subcortical regions,^83^ new contrast mechanisms,^593^ and algorithms^594^ that may highlight different aspects of brain function at the level of neuroglial tissue. Proper validation against other gold standards, such as multiphoton microscopy,^595^ will be critical to provide a sound physiological underpinning for these and other emerging OCT techniques.

### 7.2 Two-Photon O_2_ Imaging

High-resolution, real-time quantification of the partial pressure of $O_{2}$ (${\mathrm{pO}}_{2}$) *in vivo* has long been a much-desired objective.^596^^–^^599^ Development of 2P-enhanced oxygen probes (Sec. 3.8)^124^^,^^125^^,^^275^^,^^600^^–^^603^ and 2PLM of ${\mathrm{pO}}_{2}$ in the brain^268^^,^^269^ have provided important insights into the brain oxygen metabolism. Among them are confirmation of the “initial dip” in ${\mathrm{pO}}_{2}$ upon neuronal activation,^269^^,^^270^ evidence for artero-venous diffusional shunts in the brain^269^ and experimental demonstration of diffusional gradients in $O_{2}$ around individual RBCs—the so-called Erythrocyte-Associated Transients (EATs).^269^^,^^604^

**Fig. 32:**
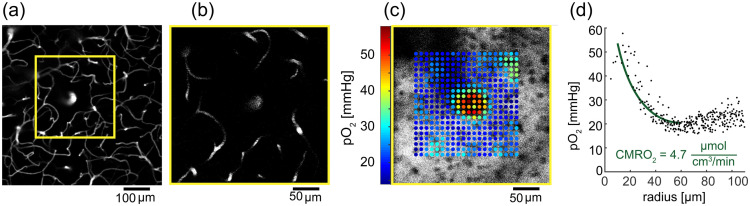
2P imaging of tissue ${\mathrm{pO}}_{2}$. (a) MIP of a 2P vascular image stack of the top 200  μm of cerebral cortex. Contrast is due to intravascular fluorescent dye. (b) Zoomed-in measurement plane. (c) Color-coded ${\mathrm{pO}}_{2}$ measurement points overlaid onto the survey phosphorescence image. (c) ${\mathrm{pO}}_{2}$ as a function of distance from the center diving arteriole.

This technology also enabled estimation of the cerebral metabolic rate of $O_{2}$ (${\mathrm{CMRO}}_{2}$), a notoriously challenging task,^605^ based on ${\mathrm{pO}}_{2}$ gradients immediately surrounding diving arterioles (Fig. 32).^574^^,^^575^ This method relies on 2 assumptions. The first assumption is that periarterial tissue gets all its $O_{2}$ from the arteriole. This is justified by the absence of capillaries within the periarteriolar space,^606^ the presence of a ${\mathrm{pO}}_{2}$ gradient in tissue around diving arterioles,^270^^,^^575^ and a ${\mathrm{pO}}_{2}$ decrease within arterioles from the vessel center to the vessel wall indicating the $O_{2}$ leaves through the arteriolar wall. This organization can be approximated by Krogh’s model of $O_{2}$ diffusion from a cylinder^607^ where the diving arteriole serves as a single $O_{2}$ source for the periarterial tissue. With this model, one can estimate ${\mathrm{CMRO}}_{2}$ from maps of periarterial tissue ${\mathrm{pO}}_{2}$. Recently, this method was used to evaluate baseline ${\mathrm{CMRO}}_{2}$ across layer in the mouse cortex.

We are only starting to understand microscopic $O_{2}$ metabolism, and many questions remain. In current practice, ${\mathrm{pO}}_{2}$ measurements are acquired in a “point scan” mode, where the laser beam jumps from point to point. Interleaving of point scan 2PLM with traditional line scan fluorescence measurements would allow near-simultaneous probing of ${\mathrm{pO}}_{2}$ and other parameters, such as neurotransmitter release and vasodilation, needed to address important questions about regulation of $O_{2}$ delivery to cerebral tissue. Combined with imaging of glucose metabolites (see Sec. 3.6), ${\mathrm{pO}}_{2}$ measurements will expand our understanding of the relative contribution of oxidative and non-oxidative metabolic pathways in the brain across different conditions.

### 7.3 Photoacoustic Imaging

Photoacoustic (PA) tomography (PAT) is a rapidly developing modality that combines optical excitation with ultrasonic detection and can be used over a range of spatial scales at high speed. PAT can utilize endogenous (label-free) contrasts and exogenous probes to provide anatomical, functional, histologic, metabolic, and molecular imaging.^579^^,^^608^^,^^609^

In PAT, non-ionizing laser pulses (∼ps−ns) are directed to the tissue for excitation. Some of the delivered optical energy is absorbed by the tissue and converted into heat. The heat then induces a pressure rise through thermoelastic expansion. The pressure rise launches an ultrasonic wave, which is referred to as a PA wave. The PA wave is detected by ultrasonic transducers to form an image by a computer. PAT has two primary incarnations: reconstruction-based photoacoustic computed tomography (PACT) and focused-scanning-based photoacoustic microscopy (PAM).^579^

**Fig. 33:**
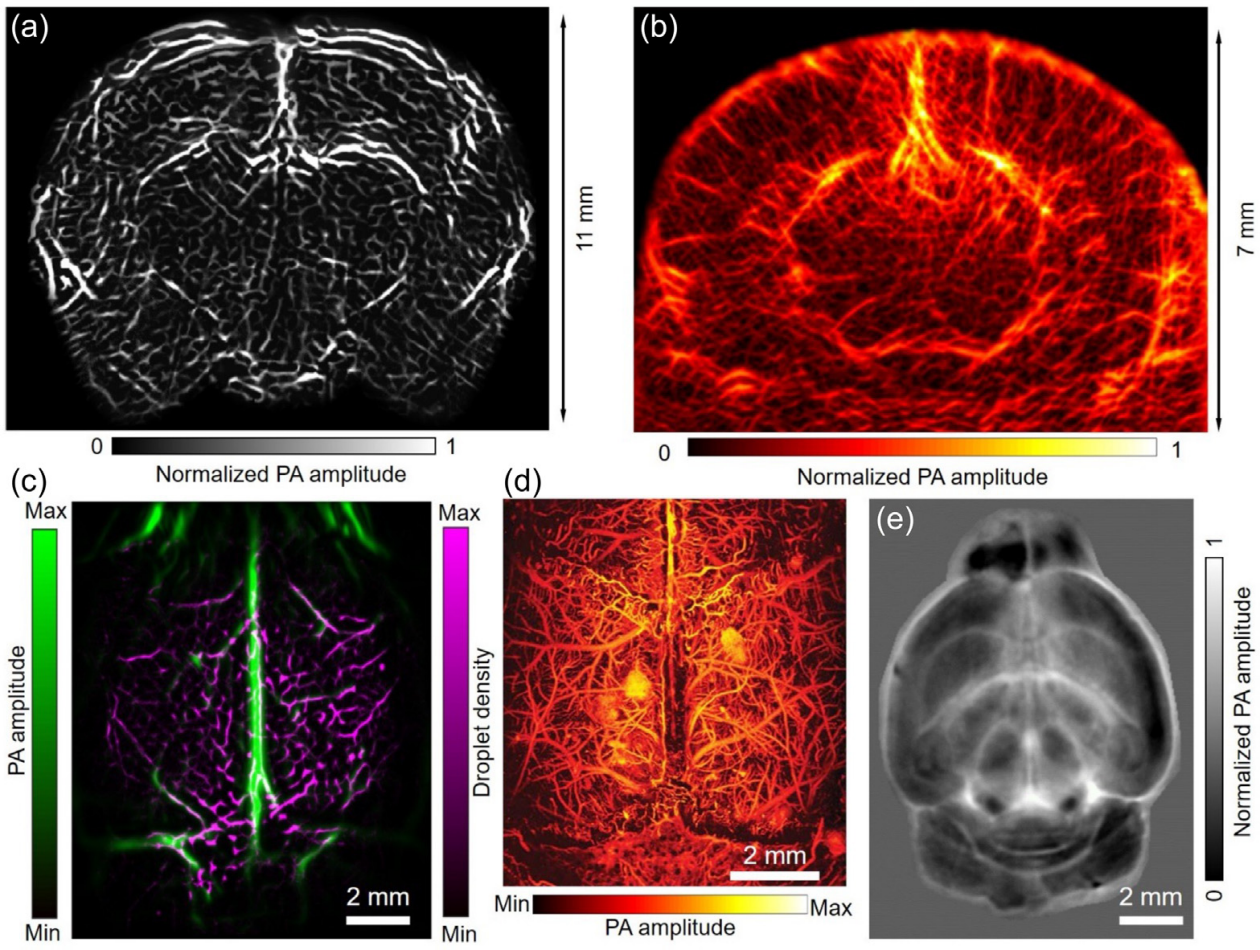
PAT of the brain. (a) *In vivo* PACT image of a whole rat brain in the coronal plane.^610^ (b) *In vivo* high-resolution PACT image of a mouse brain in the coronal plane.^611^ (c) *In vivo* superresolution PACT image of a mouse brain, where green shows the conventional PACT signals, and magenta shows the enhanced signals from localized droplets.^612^ (d) *In vivo* PAM image of a mouse brain.^613^ (e) Cross-sectional PACT image of a mouse brain at 2.8 mm depth.^614^

PAT has found broad applications in neuroscience (Fig. 33). PAM has been used to map cortical vasculature in the mouse brain and quantify oxygen saturation and total concentration of hemoglobin at the single capillary level, based on an endogenous PA contrast—hemoglobin absorption.^613^ Label-free PACT has imaged detailed mouse brain structures,^614^ provided a measurement of the whole rat brain resting-state functional connectivity,^610^ and visualized the epileptic wave propagation across the whole mouse brain during a seizure.^611^ More recently, PACT has imaged brain activity in adult humans.^615^ Exogenous PA probes are also being developed to monitor glucose metabolism and detect rapid voltage and ${\mathrm{Ca}}^{2+}$ responses to external stimulations in live animals.^616^^,^^617^

In the future, we expect that PAT will visualize action potential pulses in the deep brain in rodents with the development of novel $\text{voltage}/{\mathrm{Ca}}^{2+}$ indicators operating in the red or near-infrared spectral range. We also expect PAT to be translated to adult human brain imaging, while its current primary barrier is the skull. The adult human skull severely attenuates the excitation light and the emitted PA waves and strongly distorts the PA waveforms, deteriorating image quality. A potential solution is to combine PAT with x-ray computed tomography (CT) or MRI, which can provide accurate skull information to correct for the wavefront distortion. In addition, machine learning methods may play a revolutionary role in this problem due to their high tolerance to skull modeling errors and high computational speed.

## 8 Hybrid and Multimodal Approaches

In contrast to optical imaging technologies based on detection of photons, hybrid technologies rely on absorption of photon energy to produce other physical phenomena. Above, we discussed photoacoustics (Sec. 7.3) where absorption of photon and heating launches an ultrasonic wave.^579^ Ultrasound (US) is also being explored to steer the light in tissue^618^ and in US-assisted optical imaging (Sec. 8.1).^619^^–^^622^ Recently, the photoacoustic process has been used for spatially confined neuronal stimulation.^623^

Another approach is to combine those technologies that do not interact serving as parallel and complementary information channels extending the spatiotemporal scale, resolution and specificity of each modality alone. For example, implanted “neurophotonic” probes have been developed bearing LEDs and electrodes along the penetrating shanks; LEDs deliver the light required for optogenetics actuation of light-gated ion channels and ion pumps, and electrodes record neuronal activity.^624^^–^^626^ An alternative solution for depth-resolved optogenetic actuation is tapered optical fibers that allow the light to exit at different locations along the tapered fiber end depending on the angle of incidence of the laser beam entering the fiber. Recording electrodes printed along the fiber enable simultaneous electrophysiological recordings (Sec. 8.2). Another multimodal approach is combining optical imaging with electrophysiological recordings using optically transparent electrode arrays^627^^–^^629^ (Sec. 8.3). Integration of photodetectors along the shanks of penetrating neurophotonic probes is also being explored.^626^ Our final example is simultaneous optical imaging (and optogenetic stimulation) with fMRI^630^^–^^634^ (Sec. 8.4) that can help physiological underpinning of noninvasive imaging signals and human translation.

### 8.1 Ultrasound-Enabled Deep Fluorescence Imaging

Light scattering in brain tissue is a barrier to studying the living brain beyond superficial depths. Conventional optical focusing methods rely on ballistic photons, which exponentially decrease with depth. Loss of photons to scattering limits the signal that can be achieved at the focal spot. In addition, scattered photons contribute to out-of-focus excitation. Taken together, these two effects decrease the signal to background ratio (SBR) with depth. Ultrasound (US) scatters less than light in tissue and can be used to image beyond superficial depths, but in general has lower specificity compared to what can be achieved with targeted fluorescent probes.

**Fig. 34:**
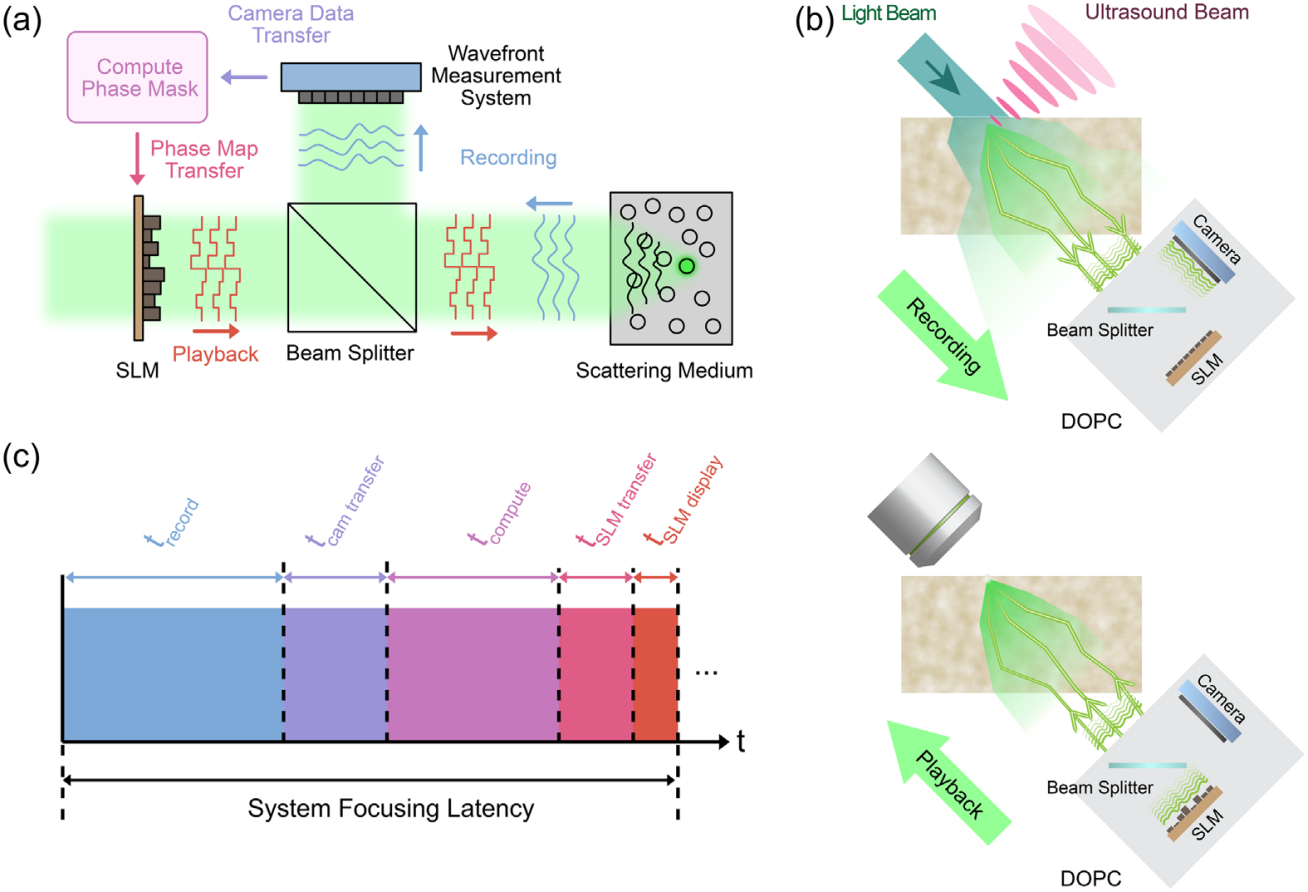
Wavefront shaping system design. (a) The wavefront shaping system consists of 5 major steps: Recording, camera data transfer, phase mask computation, phase map transfer, and phase map display and playback. First, the scattered light from a guidestar inside the tissue is captured and collected by the wavefront measurement system. Depending on the type of guidestar, the measurement system may use interferometry to detect the signal from the guidestar (e.g., with an ultrasonic guidestar) or simply measure the bulk signal after filtering (e.g., fluorescence from a particle or cell). The captured data from the camera in the measurement system is then transferred to a computing device such as a PC or FPGA where it is processed to find the desired phase map to send light back to the guidestar. Then, this phase mask is transferred to the SLM and displayed to form the shaped playback wave. (b) An example of a wavefront shaping system used to focus light in brain tissue using time-reversed US-encoded light focusing. The US-tagged light is recorded by the camera and then used to calculate the correct wavefront solution to playback a light focus at the previous location of the ultrasonic focus. (c) The sum of the time required to perform each step of the wavefront sensing and shaping process forms the system latency. Typically, the wavefront measurement time is the most significant portion of the process, but when large pixel number cameras and SLMs are used, the time for data transfer becomes significant as well. Parallel processing on embedded systems such as FPGAs can be used to enhance processing time. Furthermore, SLM technologies such as DMDs can be used to reduce the display time to several tens of microseconds as compared to the much slower, millisecond-order liquid-crystal based SLMs.

US has been used to assist optical imaging and photostimulation and has also been explored as a guidestar mechanism for optical wavefront shaping (Fig. 34). Optical wavefront shaping works by tailoring the input optical wavefront to counteract scattering as it travels through the tissue. The scattering properties of tissue (ignoring the contributions by blood cells) is static on the timescale of several seconds at depths up to a few millimeters.^635^ Within this time window, knowledge of the relevant parts of the transmission matrix allows the incident wavefront to be pre-distorted to form a desired pattern inside the tissue. In Sec. 5.8, we discussed the use of fluorescent guidestars in multiphoton microscopy. Non-optical guidestars can greatly extend the penetration depth of optical techniques.

The fundamental property that allows the scattering behavior of tissue to be characterized and compensated for is the deterministic nature of elastic scattering.^636^ In practice, this property is exploited by latching onto a signal from inside the sample provided by a guidestar to find the needed part of the transmission matrix and correctly modulate the incident light. The predominant methods to find this correct input pattern rely on either feedback optimization or direct conjugation of the signal from a guidestar. In addition to ultrasonic^637^ and fluorescent guidestars,^638^ other modalities have been explored including kinetic,^639^^,^^640^ magnetic particle,^641^ and photoacoustic^642^ guidestars. The performance of these systems is typically benchmarked by the number of independent elements of the transmission matrix that can be controlled and the latency between wavefront recording and playback. The spatial resolution in each case is limited by the nature of the guidestar.

Using wavefront shaping, the penetration depth is no longer limited by scattering (i.e., the transport mean free path) but by the absorption limit. This limit can be several centimeters deep within tissue at wavelengths where absorption is low in the near-infrared regime, and wavefront shaping systems have been demonstrated to successfully focus at depths of up to 2.5 cm in chicken breast and 10 cm in tissue-mimicking phantoms.^643^

However, there are still significant challenges imposed by the temporal dynamics of light scattering in the tissue. *In vivo*, blood flow (i.e., moving red blood cells, RBCs) is a major contributing factor to scattering dynamics. Due to RBC flow, the measured transmission matrix elements can become outdated within several milliseconds even at a depth of 1 millimeter and, due to the nonlinear relationship between scattering path length and penetration depth, this decorrelation time decreases below one millisecond at a depth of only 2 millimeters.^644^ This requires low latency wavefront sensing and shaping systems to maintain the correct wavefront solution. To date, state-of-the-art systems have achieved latencies on the order of a few milliseconds with control of 10^5^ optical modes by leveraging fast waveform modulators such as digital micromirror devices.^645^ While lower latencies can be achieved with faster modulators such as acousto-optic^646^ or microelectromechanical light valves,^647^ these techniques typically only offer control over several hundred optical modes.

Recently, a proof-of-principle for another US-enabled fluorescence imaging method has been demonstrated that is not limited by fast speckle decorrelation.^619^ Rather, the fluctuating speckle intensity at a point of interest is co-encoded in the emitted fluorescence and US-modulated light. Using the US-modulated transmissions as a decoding key, the fluorescence from the target point at the US focus can be extracted from the overall fluorescence emission. A number of technical challenges associated with this approach still need to be addressed including decreasing the measurement time and enlarging the field of view. In the meanwhile, wavefront shaping remains the most promising approach for overcoming light scattering.

In the future, we expect wavefront shaping technologies to develop along two main axes:

(1) For some applications where the penetration depth is relatively shallow, such as imaging of deep cortical layers, wavefront distortions are more strongly associated with gross tissue heterogeneity and less impacted by blood flow. These distortions can be addressed with adaptive optics solutions such as the ones described for multiphoton microscopy in Sec. 5.8.

(2) To be able to correct for dynamic light scattering induced by moving RBCs, further technological development is needed to improve system latency by leveraging fast wavefront shaping devices like digital micromirror devices (DMD) and parallelization to solve the data transfer and processing bottlenecks. Currently, wavefront shaping techniques have largely been demonstrated in carefully controlled experiments. To move toward broader adoption, it is necessary to develop strategies tackling the temporal dynamics, especially to reach the ultimate depth limits, and to address practical implementation concerns such as how to interface the wavefront shaping systems with the types of configurations commonly used in neuroscience experiments (e.g., freely moving rodents). To achieve these goals, we envision a wavefront shaping device which will integrate the wavefront sensing and shaping elements such as the one demonstrated in Ref. 648, will be critical to address the wavefront measurement bottleneck by parallelizing the measurement and guaranteeing alignment between the measured and shaped wavefronts.

### 8.2 Tapered Optical Fibers as Implantable Multimodal Neuronal Interfaces

Tapered optical fibers have recently emerged as a new platform for implantable devices to optically interact with deep structures of the mouse brain. These fibers can both deliver or collect light over either large brain volumes or spatially confined subregions all around the conical shape, while generating little to no damage to the brain tissue, comparable to that caused by sharp electrodes used for electrophysiological recordings.^649^^–^^651^

**Fig. 35:**
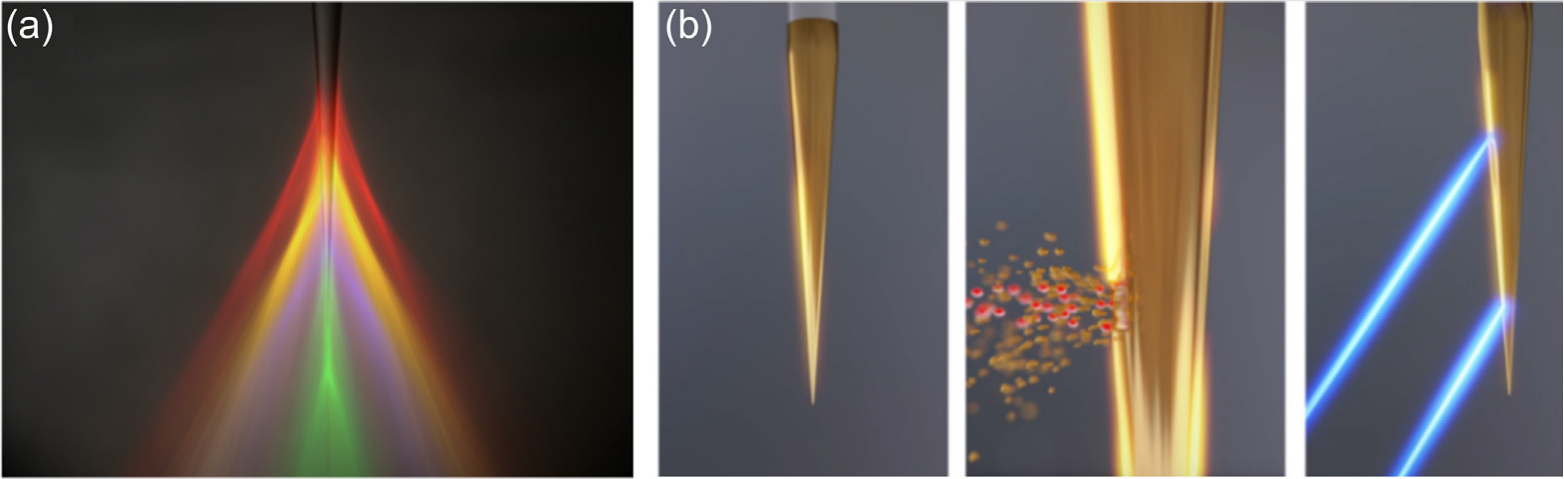
Tapered optical fibers as neuronal interfaces. (a) Example of how modes of different order outcouple at different sections of a TF. Image is in false colors. (b) High resolution patterning allows structuring the non-planar edge of metal-coated tapered optical fibers.

Tapered optical fibers have the unique ability of enabling depth-resolved optogenetics and fiber photometry by exploiting the modal properties of the narrowing waveguide: high and low order modes can exchange energy with the brain tissue at different depths along the implant, resulting in a fully integrated and implantable mode-division multiplexer/demultiplexer (MODEM) for brain microcircuitry [Fig. 35(a)]. For example, if the tapered fiber is implanted in cerebral cortex, one can control which cortical layer is being illuminated by changing the input angle of light entering the fiber [see different colors in Fig. 35(a)]. Due to modal mixing of propagating light in the waveguide, the resolution of this technique is in the order of a few hundreds of micrometers. For obtaining finer spatial resolution of light delivery and/or collection, micro and nanofabrication can be employed to coat the fiber with a reflective metal thin layer followed by etching small windows along the tapered end [Fig. 35(b)].^652^^–^^654^

Together with the low invasiveness, this technology has contributed to the dissection of basal ganglia neuronal circuitry in the mouse brain: motor-action-related topographical organization of the striatum was probed by depth-selective optogenetic stimulation,^655^ while differentiated dopamine transients in dorsal and ventral striatum in reward processing were observed by applying depth-resolved fiber photometry.^651^ From the perspective of advanced detection of photons generated in deep brain regions, tapered fibers are compatible with time-correlated single photon counting technologies recently employed to probe the biochemical state of neurons,^656^ and can help in bringing this technology to deeper (subcortical) brain structures.^657^

In addition to the ability of carrying excitation or fluorescence light, tapered fibers can host extracellular recording electrodes, resulting an interesting paradigm for probing small brain volume with spatially confined light delivery and electrophysiological recordings.^658^ The main challenge in this respect is the non-planar surface of the narrowing waveguide, which does not allow using standard micro- and nano-fabrication techniques.

The photonic properties of tapered fibers also offer the opportunity to control nanoscale light-matter interactions on the fiber surface^659^ providing a suitable platform to translate *in vivo* the many options for enhanced biomolecular interfacing using plasmonic resonances that have been proposed *in vitro*.^660^ These include Surface Enhanced Raman Scattering (SERS), refractive index sensing, metal-enhanced fluorescence, and highly localized, ultrafast heat delivery in combination with the modulation of the radiative field through sub-wavelength apertures for light delivery and collection in highly scattering tissue.

Next generation technology will therefore have to focus on the development of methods to integrate complex optical and optoelectronic elements on the edge of the fiber taper, aiming at the realization of multifunctional neuronal interfaces targeting both small and wide brain volumes.

### 8.3 Transparent Electrode Arrays for Multimodal Brain Mapping

Optical methods for monitoring and stimulating neuronal activity enable investigating large-scale neuronal networks up to subcellular resolution, and across multiple signaling dimensions. Combining optical tools with electrophysiological recordings allows comprehensive investigations of brain circuits *in vivo*, multi scale, as it leverages the finely tuned spatial resolution of optical imaging with the high temporal resolution and fidelity of electrophysiology. Multimodal studies, however, are not possible with conventional opaque metal microelectrodes, as they block optical access and suffer from photovoltaic artifacts that can contaminate the recordings.^661^^,^^662^

**Fig. 36:**
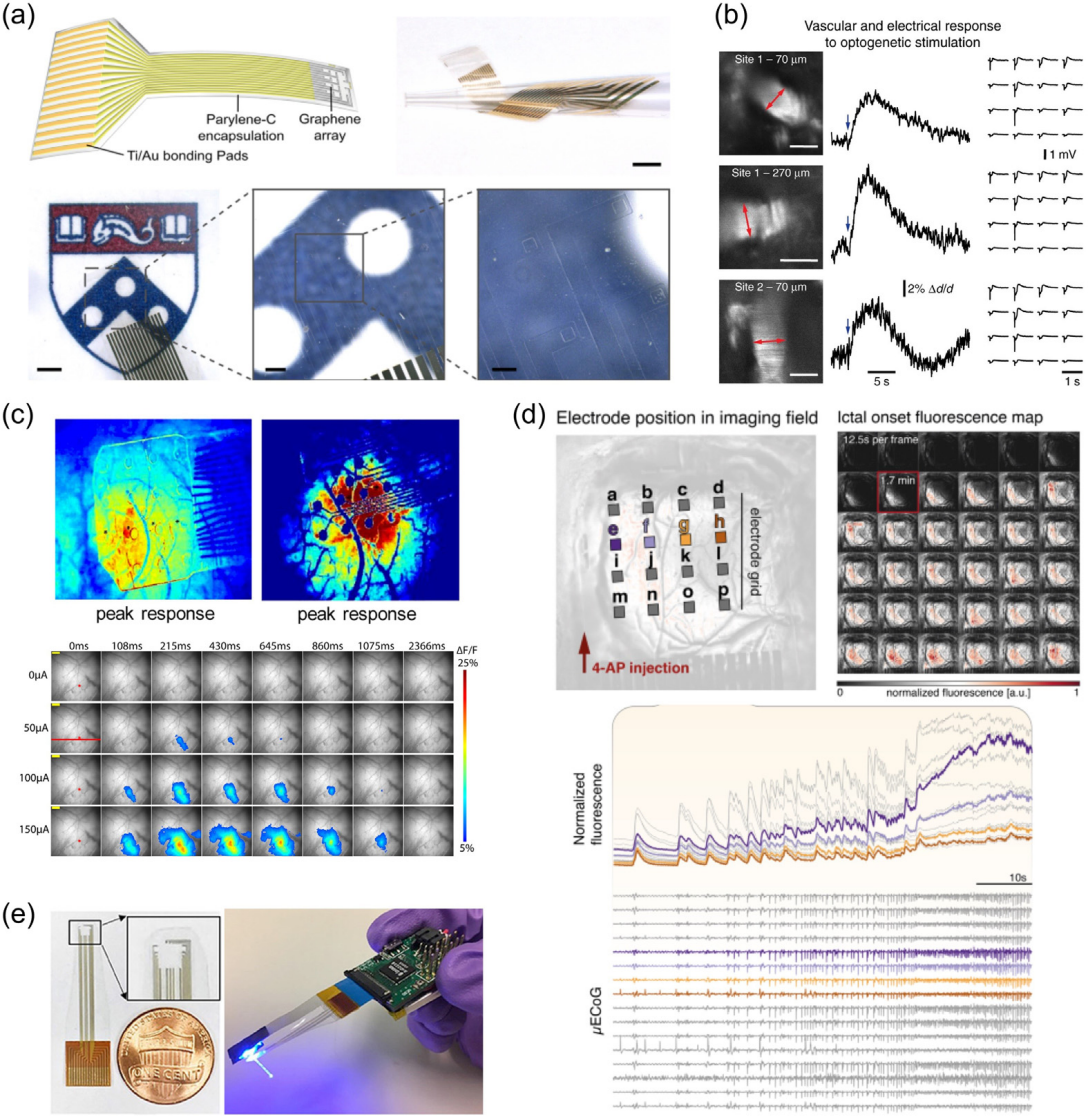
Multimodal measurements and interrogation of brain activity using transparent electrode arrays and neurophotonic technologies. (a) (Top) Design of a flexible, transparent 16-channel microECoG array for multimodal mapping the dynamics of epileptiform discharges in mouse models.^663^ The array is a grid of 50  μm×50  μm square electrodes with 500  μm spacing (total recording area: 1.55 mm × 1.55 mm, total array footprint 2.75 mm × 2.75 mm. Scale bar: 2 mm). (Bottom) Photographs illustrating the optical transparency of the array in the recording area. Scale bars, left to right: 1 mm, 250  μm, and 100  μm. Reproduced from Ref. 663. (b) Simultaneous optogenetics, recording and hemodynamic imaging in Thy-Cr2 mice through graphene arrays.^662^ (Left) Line-scan imaging of arteriolar dilation induced by optogenetic stimulation under two different electrode sites (marker: FITC-dextran, red arrows show line-scan location). Scale bars: 50  μm. (Middle) Average vascular diameter change and (right) light-evoked LFPs recorded at the graphene electrode sites. Reproduced from Ref. 662. (c) Maps of cortical responses to co-localized electrical stimulation delivered through graphene electrodes in GCamp6f mice.^664^ (Top) ${\mathrm{Ca}}^{2+}$ fluorescence intensity in response to cortical electrical stimulation under (left) a graphene and (right) a platinum array of the same size. (Bottom) Spatiotemporal maps of cortical activation following electrical stimulation at varying intensities delivered from a transparent graphene electrode (marked with the red star). Adapted from Ref. 664. (d) Multimodal analysis of ictal state transitions in acute mouse preparations induced by 4-aminopyridine (4-AP).^663^ (Top left) Schematics of the relative graphene contact positions and 4-AP application site. (Top right) Propagation of the ictal wavefront at seizure onset mapped with wide-field ${\mathrm{Ca}}^{2+}$ epifluorescence imaging. (Bottom) ${\mathrm{Ca}}^{2+}$ fluorescence intensity beneath each electrode and simultaneous microECoG recordings from the graphene array during seizure onset. Adapted from Ref. 663. (e) Closed-loop graphene device for artifact-free optogenetics. (Left) Photographs of the transparent graphene array and (right) the fully assembled battery-powered, closed-loop device integrating the graphene array with an optic fiber for optogenetic stimulation. Adapted from Ref. 665.

Recent progress in materials and fabrication processes for neuroelectronic interfaces has led to the development of transparent microelectrode arrays for simultaneous, artifact-free imaging and recording in the same brain location. These devices typically consist in planar arrays of transparent microscale electrodes (spatial scales ranging from 50  μm to 250  μm) patterned onto <20  μm thin, flexible polymeric films (e.g., parylene, polyimide, PET). The majority of the transparent devices proposed thus far are micro-electrocorticography (microECoG) arrays [Fig. 36(a)],^661^^–^^663^ although there are a few examples of laminar electrodes for intracortical recordings.^627^^,^^666^

The enabling technology behind transparent electrodes are novel nanomaterials that combine high optical transparency for imaging with the electrical conductivity required to record neuronal signals. Graphene—a 2D monoatomic layer of carbon atoms—in particular has emerged as the material of choice for many of these technologies, due to its remarkable combination of transparency, electrical conductivity, biocompatibility, low noise, and resistance to corrosion.^667^^–^^669^

From the initial works demonstrating co-localized ${\mathrm{Ca}}^{2+}$ imaging with electrophysiology^627^ and optogenetics^661^ through graphene microECoG arrays, transparent electrodes have been used for simultaneous LFP recordings, 2P ${\mathrm{Ca}}^{2+}$ imaging in layers II/III of the mouse cortex, optogenetics, and hemodynamic imaging [Fig. 36(b)],^662^ for delivering cortical stimulation and concurrently mapping the evoked cortical activation patterns [Fig. 36(c)],^664^ and for studying the onset and spreading dynamics of epileptiform discharges *via* simultaneous electrophysiology and wide-field ${\mathrm{Ca}}^{2+}$ imaging [Fig. 36(d)].^663^ Graphene electrodes have also been integrated with optic fibers to realize battery-powered devices for closed-loop optogenetics [Fig. 36(e)].^665^

Despite the exciting results, the main drawbacks of transparent graphene microelectrodes are the high interface impedance—which limits the ability to record single extracellular spiking activity—and the complexity of processing and fabrication. In the attempt to overcome these limitations, a number of alternative materials have been explored for transparent microelectrodes, including carbon nanotubes (CNTs),^670^ platinum nanoparticle coatings,^671^ conductive polymers (e.g., PEDOT-PSS^628^), and gold nanomeshes.^629^^,^^666^

In addition to addressing the impedance, temporal resolution and scalability issues, future work is needed to evaluate the longevity and biocompatibility of graphene-based and other transparent electrode technologies for chronic *in vivo* implants. Furthermore, innovative approaches combining surgical preparations with custom device configurations are required to conduct multimodal investigations into deeper subcortical regions, which are inaccessible to planar microECoG arrays. Finally, there is a lack of established frameworks for analyzing high-dimensional datasets operating on different spatial and temporal scales, such as those emerging from multimodal optical and electrophysiological mapping of neuronal activity.

### 8.4 Optical Imaging and Functional Magnetic Resonance Imaging (fMRI)

Blood-oxygen-level-dependent (BOLD) fMRI is a widely implemented, clinically and preclinically accessible, noninvasive neuroimaging tool with whole-brain coverage. Despite these strengths, BOLD-fMRI data have relatively low spatiotemporal resolution (sub-millimeter to millimeter, ∼1  Hz), and are insensitive to the cellular origins of activity. This can limit the utility and interpretability of BOLD-fMRI measurements.^672^ As a complementary neuroimaging toolset, optical imaging methods have high reporter specificity and spatiotemporal resolution (micron to sub-millimeter, tens to hundreds of Hz), but are limited to optically accessible tissue and to applications in animal models due to the introduction of fluorophores which requires invasive manipulation of the nervous system. Together, optical imaging and BOLD-fMRI provide a link between reporter specific measurements of activity and the most widely used method for assessing human brain function as well as a means of crossing disparate spatiotemporal scales.

**Fig. 37:**
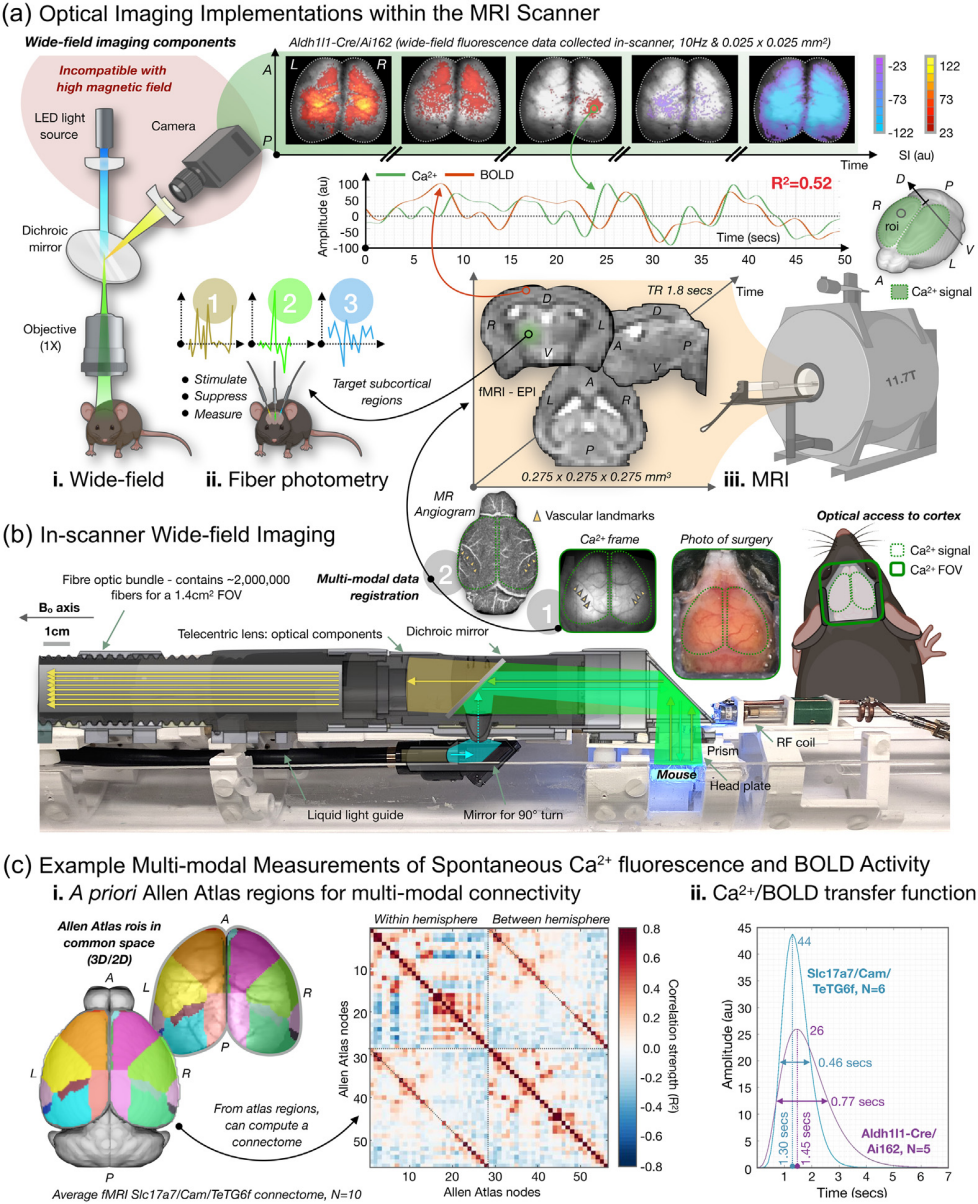
Concurrent optical imaging and fMRI. (a) Optical imaging implementations within the MRI scanner. Schematic of typical wide-field imaging components (i.). Excitation light is delivered (LED light source), passes through a dichroic mirror, and is focused onto the mouse cortex (1X objective). Emitted fluorescence signal is relayed to a camera and recorded. Example imaging frames from a movie recorded from a mouse expressing GCaMP in glia (Aldh1l1-Cre/Ai162) (top right). Data are collected in-scanner. Blooms of increased activity are in hot colors; epochs of decreased activity are in cool colors. Schematic of a typical fiber photometry experiment (ii.). Excitation light delivered and signal recorded through long fiber optic cables. Both (i.) and (ii.) can be performed within an MRI scanner (iii.) where simultaneous BOLD-fMRI data can be recorded. Wide-field imaging yields measures of cortical activity (2D images), while fiber photometry can target subcortical areas (1D time courses). Simultaneously recorded glia-${\mathrm{Ca}}^{2+}$ (green) and BOLD (orange) spontaneous activity from a cortical roi are plotted (middle). In this example, these signals are moderately correlated (Pearson’s correlation $R^{2}=0.52$, P < 0.05). (b) In-scanner wide-field imaging. Components and light path are indicated (image reproduced from Ref. 630). For wide-field imaging, optical access to the cortex is gained by resecting the scalp and covering the skull in dental cement, glue, and glass (right). The wide-field imaging data can be registered to the MRI data using the middle cerebral arteries as anatomical landmarks and an MR angiogram. Example multi-modal measurements of spontaneous ${\mathrm{Ca}}^{2+}$ fluorescence and BOLD activity. (c) With multi-modal data registered to a common space (i.), ROIs can be imposed (e.g., Allen Atlas regions) and a connectome computed. Multi-modal spontaneous activity can be used to compute a between contrast transfer function (ii.). Here, gamma-variant fitting^630^ was applied to compute a transfer function using simultaneously recorded spontaneous BOLD activity and excitatory neuronal ${\mathrm{Ca}}^{2+}$ activity (Slc17a7/Cam/TeTG6f, N = 6, plotted in blue) or glia ${\mathrm{Ca}}^{2+}$ activity (Aldh1l1-Cre/Ai162, N = 5, plotted in purple). Abbreviations: functional magnetic resonance imaging (fMRI, MRI, MR), light emitting diode (LED), anterior (A), posterior (P), right (R), left (L), dorsal (D), ventral (V), Pearson’s correlation ($R^{2}$), region(s) of interest (roi, rois), signal intensity (SI), arbitrary units (au), blood-oxygen-level-dependent (BOLD), seconds (secs), echo planar imaging (EPI), repetition time (TR), field of view (FOV), radio frequency (RF).

BOLD-fMRI and optical imaging can be performed concomitantly (Fig. 37), or serially, to interrogate the physiological reporter specific underpinnings of the BOLD-fMRI signal, explore brain-wide circuit- and network-level activity patterns, and neuro-glio-vascular coupling in health and disease. To collect BOLD-fMRI and optical imaging data from the same subject, significant experimental challenges must be overcome to accommodate the confined space and high magnetic field of the MR-scanner without compromising the optical signal. This can be particularly difficult if the multi-modal data are acquired simultaneously which is necessary when studying certain aspects of brain function such as spontaneous activity (without external stimuli).^673^^,^^674^

To date, there are a handful of implementations of BOLD-fMRI with simultaneous single/double-fiber photometry to yield ‘point’ measurements, or one-dimensional time-courses of optical data and two reports of simultaneous wide-field imaging using fiber-optic bundles to obtain two-dimensional optical images of cortical activity.^630^^,^^631^^,^^634^^,^^675^^–^^681^ Experimentally, each in-scanner optical imaging implementation includes a means of delivering excitation light to, and relaying optical signal from within the bore, reducing MR-susceptibility artifacts caused by surgeries that yield optical access to the brain, minimizing motion, and eliminating or keeping metal components at a safe distance from the MR-scanner. The signal-processing and analyses of these data follow a variety of strategies that depend on the implementation and scientific question.

In the near future, we expect improved in-scanner optical imaging compatibility with state-of-the-art MRI technology (e.g., imaging with an MR-cryo-coil), experimental versatility (e.g., use of novel or multiple optical probes), and data quality (e.g., better signal-to-noise). Along with these advances in data acquisition, more complex applications will be attempted including implementations in awake behaving animals, disease models, and both young and old subjects. Further, the analyses of these multi-modal data will expand and progress through more wide-spread implementation, data sharing and cross-specialty collaboration. Ultimately, BOLD-fMRI and optical imaging methods have the potential to link animal and human studies and to provide unique insight into the functional organizing principles of the brain in health and disease.

## 9 Computational Imaging

**Fig. 38:**
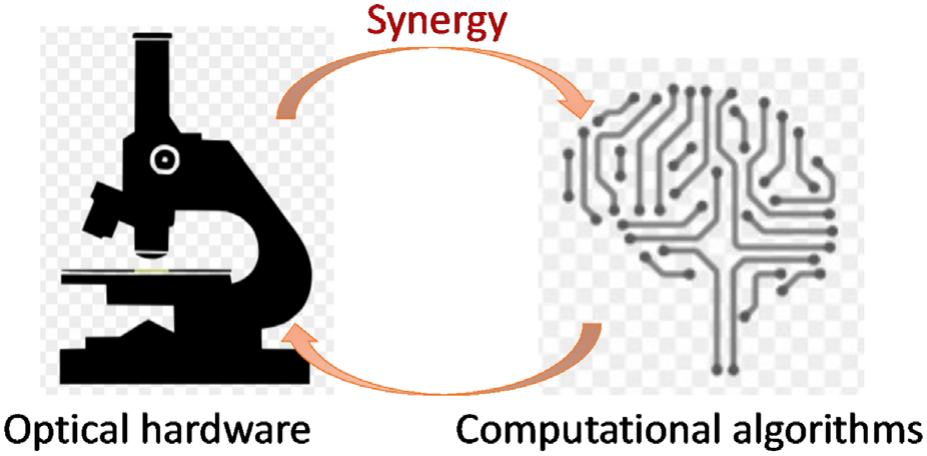
Computational microscopy synergistically combines optical hardware and computational algorithms.

Computational microscopy is a class of methods that synergistically combines optical hardware and computational algorithms for enabling novel capabilities that the optics alone do not support (Fig. 38). The motivation is to overcome the many challenges in neuronal imaging. These methods have been broadly applied to 1P or multiphoton imaging of functional indicators (e.g., ${\mathrm{Ca}}^{2+}$ imaging and voltage imaging), OCT, and PA imaging.

**Fig. 39:**
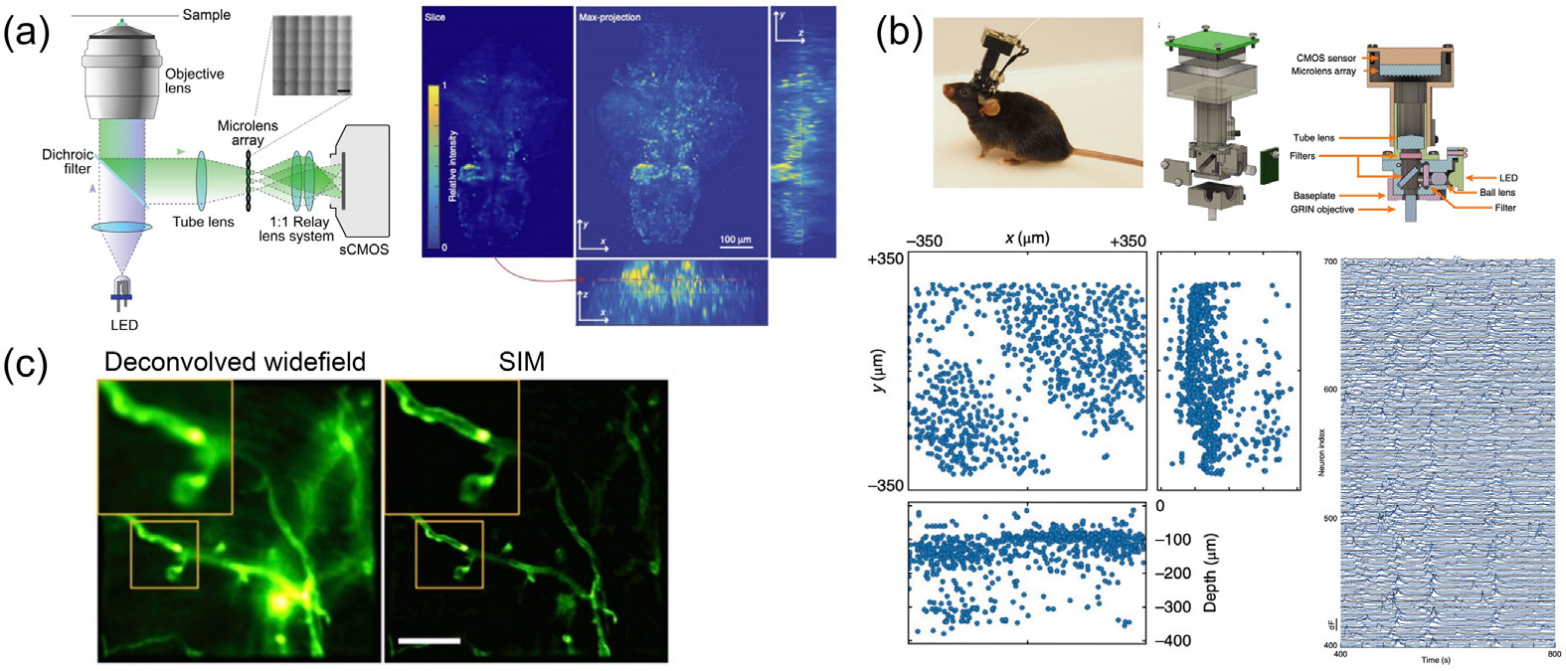
Computational microscopy enables novel imaging capabilities. (a) Light field microscopy enables single-shot high-speed volumetric neuronal imaging.^442^^,^^683^ (b) Miniaturized light field microscope allows volumetric neuronal recording in freely moving mice.^688^ (c) Structured illumination microscopy achieves super-resolution reconstruction of dendrites expressing ChR2-GFP on the mouse brain *in vivo*.^689^

The first type of challenges stem from the physical tradeoffs between several desirable imaging parameters, including SNR, imaging speed, spatial resolution, FOV, depth of field (DOF), and the size and weight of the optical device. To overcome this, new types of computational microscopes have been developed to enable high-speed volumetric imaging, enhanced resolution, and extended DOF. For example, Light field microscopy enabled single-shot *in-vivo* 3D recording of neuronal activity^442^^,^^682^^–^^687^ [Fig. 39(a)]. Similar concepts have enabled miniaturized microscopes, such as MiniLFM,^688^ Miniscope3D,^690^ CM2,^419^ and FlatScope,^691^ for 3D recording of neuronal activity in freely moving animals [Fig. 39(b)]. Structured illumination microscopy enabled super-resolution reconstruction and background suppression for *in vivo* brain imaging^689^^,^^692^^,^^693^ [Fig. 39(c)].

**Fig. 40:**
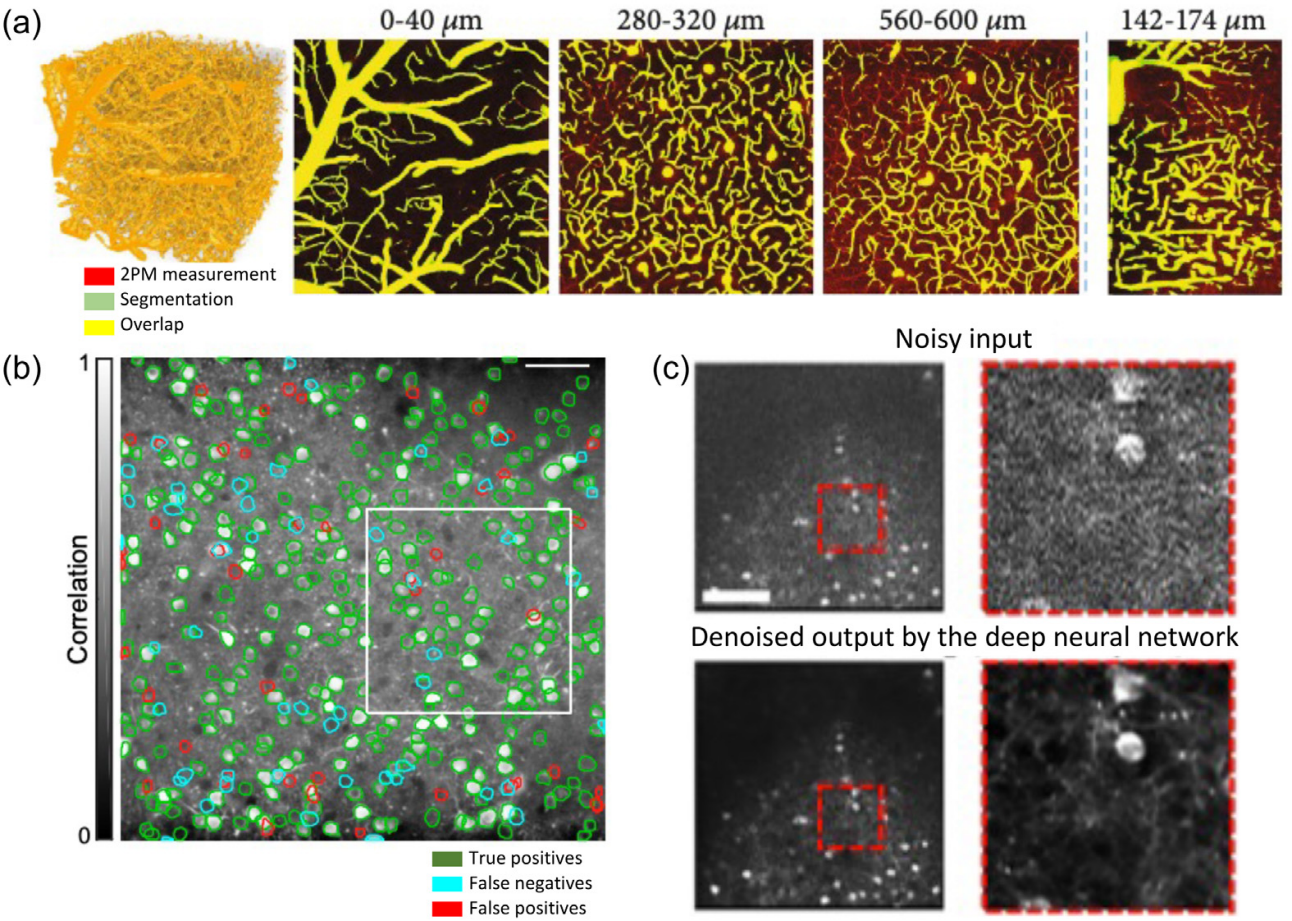
Deep learning enables state-of-the-art neuronal signal extraction. (a) Vascular segmentation from 2P imaging data.^694^ (b) Active neuron segmentation in 2P ${\mathrm{Ca}}^{2+}$ imaging.^696^ (c) Denoising 2P ${\mathrm{Ca}}^{2+}$ imaging data.^697^

The second type of challenges stem from the complexity of the large-scale multi-dimensional neuronal imaging data. To overcome this, many advanced computational algorithms have been developed to perform various signal extraction tasks, such as segmentation, enhancement of SNR, image deconvolution, and spike estimation. In the past few years, parametric and model-based techniques, such as principal component analysis (PCA), non-negative matrix factorization (NMF), and sparse deconvolution, have shown tremendous success. In recent years, data-driven machine/deep learning-based techniques have become popular and achieved the state-of-the-art performance in various tasks, such as vessel segmentation in deep brain^694^^,^^695^ [Fig. 40(a)], neuron segmentation^696^^,^^698^ [Fig. 40(b)], functional signal denoising^697^ [Fig. 40(c)], ${\mathrm{Ca}}^{2+}$ signal extraction,^699^^,^^700^ 3D deconvolution,^701^^,^^702^ and super-resolution reconstruction.^703^

In the future, we can expect that computational microscopy will continue to broadly impact both the system design and signal analysis in neuronal imaging. One promising area is to jointly optimize the optics, acquisition strategy, and computational algorithm in a holistic framework to further push the imaging performance limits. We also anticipate that more advanced deep learning methods beyond the widely used supervised learning methods will be developed, which will incorporate unsupervised learning, semi-supervised learning, physical model-based priors for more data efficient and reliable signal extraction.

## 10 Conclusions

In this Status Report, we provided a 30,000-foot view of a suite of neurophotonic methods—ranging from molecular sensors to imaging technologies that allow high-speed imaging with large fields of view—that have emerged from the BRAIN Initiative and related large-scale neuroscience efforts. For each domain, we outlined the current state-of-the-art of the respective technologies, identified the areas where innovation is needed, and provided an outlook for future directions. Our goal was not to offer a comprehensive list of all recently developed neurophotonic tools. Further, our choice was often biased toward technologies developed in our own laboratories. We hope, nevertheless, that the overall scope does justice to the breadth of the modern neurophotonics arena spanning across the spatiotemporal scales:^704^ from the nanometer scale of molecular sensors to the mesoscale of cortical columns and entire brain areas (hundreds of microns); from the nanosecond scale of fluorescent lifetime to slow changes in mood, executive function, and arousal unfolding over minutes and hours.

Most often, the invasive nature of the methods described in this report limits their direct translation to humans. Exceptions exist, including intraoperative brain imaging^705^^–^^707^ and/or other situations where the benefit overweighs the probability of the risk. For example, optogenetic methods are being tested in clinical trials aimed at (partial) restoration of vision.^708^ Maybe some of the methods presented here will evolve or inspire noninvasive techniques that can be employed in humans one day. But even in cases where the direct translation is prohibited, microscopic imaging in animals has led to better interpretation of noninvasive optical signals achievable in humans. In particular, physiological underpinning of fNIRS (as well as fMRI) signals requires an understanding of the relationship between neuronal network activity, blood flow, and $O_{2}$ consumption.^566^^,^^709^^,^^710^ Neurophotonic methods designed to noninvasively probe deep tissue and apply to humans will be the focus of our companion report.

Each of the technologies highlighted in this report has been conceived by a small group of experts, but not without input from the bigger neurophotonics community. In every case, a dialog with the community and parallel efforts elsewhere have been instrumental in driving innovation, providing a testbed for early developments, and fine-tuning the new technology to serve specific applications. Among the most significant measures of the success for these efforts are ever-expanding collaborative ties between the technology and biology experts and growing global science network across countries and continents.^3^^,^^711^

As impressive and significant as the recent progress has been, we are just getting started. Our next challenge as a community is to sustain the momentum beyond the lifetime of any individual program or initiative. Needless to say, this entails, among others, retaining and nurturing the best of our students. So, if you are a student reading these lines, we would like to conclude by speaking directly to you:

*Dear Student, thank you for making it to the end of this report. We appreciate your curiosity and persistence. At the risk of sounding self-congratulatory, we would like to tell you that each of the tools featured here is a story of ingenuity, perseverance, and tireless work. Each one is a personal story of trial and error, triumph and failure, a bold vision baked into a dry and meticulous scientific approach. These are technologies made by people. We are using light to illuminate the future, and it’s never been so bright! Come and join us in this endeavor!*
